# TL1A and IL-18 synergy promotes GM-CSF-dependent thymic granulopoiesis in mice

**DOI:** 10.1038/s41423-024-01180-8

**Published:** 2024-06-05

**Authors:** Mario Ruiz Pérez, Christian Maueröder, Wolf Steels, Bruno Verstraeten, Sahine Lameire, Wei Xie, Laura Wyckaert, Jelle Huysentruyt, Tatyana Divert, Ria Roelandt, Amanda Gonçalves, Riet De Rycke, Kodi Ravichandran, Bart N. Lambrecht, Tom Taghon, Georges Leclercq, Peter Vandenabeele, Peter Tougaard

**Affiliations:** 1 Molecular Signaling and Cell Death Unit, VIB-UGent Center for Inflammation Research, Flanders Institute for Biotechnology, Ghent, Belgium; 2 Department of Biomedical Molecular Biology, Ghent University, Ghent, Belgium; 3 Cell Clearance in Health and Disease Lab, VIB-UGent Center for Inflammation Research, Flanders Institute for Biotechnology, Ghent, Belgium; 4 Laboratory of Immunoregulation and Mucosal Immunology, VIB-UGent Center for Inflammation Research, Ghent, Belgium; 5 Department of Internal Medicine and Pediatrics, Ghent University, Ghent, Belgium; 6 VIB Single Cell Facility, Flanders Institute for Biotechnology, Ghent, Belgium; 7 VIB BioImaging Core, VIB-UGent Center for Inflammation Research, Technologiepark-Zwijnaarde 71, Ghent, 9052 Belgium; 8 Department of Microbiology, Immunology, and Cancer Biology, University of Virginia, Charlottesville, VA USA; 9 Division of Immunobiology, Department of Pathology and Immunology, Washington University School of Medicine, St. Louis, MO USA; 10 Department of Pulmonary Medicine, Erasmus MC, Rotterdam, The Netherlands; 11 Cancer Research Institute Ghent, Ghent, Belgium; 12 Department of Diagnostic Sciences, Ghent University, Ghent, Belgium

**Keywords:** Thymic Neutrophils, Emergency granulopoiesis, Thymus atrophy, Thymic GMP, Cytokine synergy, Myelopoiesis, Neutrophils

## Abstract

Acute systemic inflammation critically alters the function of the immune system, often promoting myelopoiesis at the expense of lymphopoiesis. In the thymus, systemic inflammation results in acute thymic atrophy and, consequently, impaired T-lymphopoiesis. The mechanism by which systemic inflammation impacts the thymus beyond suppressing T-cell development is still unclear. Here, we describe how the synergism between TL1A and IL-18 suppresses T-lymphopoiesis to promote thymic myelopoiesis. The protein levels of these two cytokines were elevated in the thymus during viral-induced thymus atrophy infection with murine cytomegalovirus (MCMV) or pneumonia virus of mice (PVM). In vivo administration of TL1A and IL-18 induced acute thymic atrophy, while thymic neutrophils expanded. Fate mapping with *Ms4a3*-Cre mice demonstrated that thymic neutrophils emerge from thymic granulocyte-monocyte progenitors (GMPs), while *Rag1*-Cre fate mapping revealed a common developmental path with lymphocytes. These effects could be modeled ex vivo using neonatal thymic organ cultures (NTOCs), where TL1A and IL-18 synergistically enhanced neutrophil production and egress. NOTCH blockade by the LY411575 inhibitor increased the number of neutrophils in the culture, indicating that NOTCH restricted steady-state thymic granulopoiesis. To promote myelopoiesis, TL1A, and IL-18 synergistically increased GM-CSF levels in the NTOC, which was mainly produced by thymic ILC1s. In support, TL1A- and IL-18-induced granulopoiesis was completely prevented in NTOCs derived from *Csf2rb*^-/-^ mice and by GM-CSFR antibody blockade, revealing that GM-CSF is the essential factor driving thymic granulopoiesis. Taken together, our findings reveal that TL1A and IL-18 synergism induce acute thymus atrophy while  promoting extramedullary thymic granulopoiesis in a NOTCH and GM-CSF-controlled manner.

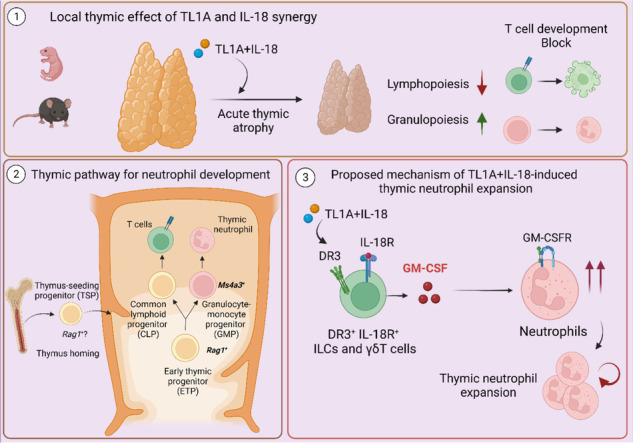

## Introduction

The thymus plays a central role in immune homeostasis, enabling the development of a repertoire of T lymphocytes directed against foreign antigens while avoiding self-reactivity [1, 2]. However, the thymus also harbors non-lymphoid cell subsets that support T-cell development [3] but may also serve other functions. Our understanding of their origin, developmental pathways, and functions is relatively limited compared with that of thymic T-lymphocyte development [4, 5]. Thymopoiesis relies on continuous seeding of hematopoietic progenitors, known as early thymic progenitors (ETPs), which migrate to the thymus in a CCR7-, and CCR9-dependent manner [6, 7]. The thymic microenvironment provides signals that direct ETPs toward T-cell development, restricting other potential fates in a NOTCH-controlled manner [8–13]. Nevertheless, ETPs comprise a heterogeneous group of non-committed progenitors that can adopt granulocyte- [14], monocyte/macrophage- [15, 16], dendritic- [17, 18], ILC- [19], B- [20], and NK- [21, 22] fates.

The thymus also contains a small fraction of myeloid cells [14, 23, 24]. Previously, it has been suggested that both the recruitment [25] and de novo development of monocytes/macrophages [15, 16] and neutrophils [14] can contribute to the thymic myeloid pool. During homeostasis, thymic neutrophils constitute ~0.05% of the total cellularity in the thymus but are increased during dexamethasone-induced thymus atrophy [26]. Fate mapping studies using *Rag1*-Cre reporter mice suggested that, unlike thymic macrophages [15, 24], thymic neutrophils arise from thymic progenitors [14]. Furthermore, IL-7Rα^-/-^ progenitors are outcompeted by IL-7Rα^+/+^ progenitors in mixed bone marrow chimeras to become thymic granulocytes [14]. These findings imply that unlike thymic macrophages [15] and bone marrow-derived neutrophils [27], thymic-derived neutrophils require IL-7 for their development.

Systemic inflammatory conditions, such as viral [28], bacterial [29], parasitic infections [30], or sepsis [31], affect thymus homeostasis, leading to acute thymic atrophy and resulting in defects in early lymphopoiesis. Interestingly, systemic inflammation can also tailor hematopoiesis “on demand” by promoting granulopoiesis at the expense of lymphopoiesis [32–34]. During development, or later in life during pathophysiological conditions, extramedullary granulopoiesis can occur in the liver, spleen, lungs, and lymph nodes [35, 36]. The potential contribution of the thymus as an extramedullary niche during inflammation has not been investigated. Severe inflammation can trigger *“emergency granulopoiesis”* [37–39], which involves increased neutrophil production, extended neutrophil lifespan, and the release of the *“rapid mobilizable pool”* into circulation [40]. Although the classical model of granulopoiesis has recently been refined [41], it is unclear whether this developmental model applies to extramedullary granulopoiesis.

Notably, IL-18 can play a role in enhancing granulopoiesis [34], while in the thymus, IL-18 can result in the expansion of ETPs [42] and exert localized effects on thymic homeostasis [43]. In fact, in vivo depletion of IL-18R^+^ innate lymphoid cells (ILCs) prior to radiation-induced damage markedly improved thymic regeneration, indicating that IL-18 inhibits thymic repair after acute injury [43]. In addition, death receptor 3 (DR3) was identified as an important regulator of negative selection during thymic development [44]. DR3 binds to TNF-like protein 1A (TL1A) and shapes adaptive and innate responses in lymphocytes and myeloid cells [45–47]. Notably, DR3 knockout mice exhibit normal levels of neutrophils in homeostasis but lower neutrophil counts after challenge with *Staphylococcus epidermidis* [48], revealing that DR3 is a potential regulator of neutrophil numbers during inflammation [48, 49]. Peripheral T cells coexpressing DR3 and IL-18Rα are potent cytokine producers [47, 50], and several studies have shown that TL1A and IL-18 signaling leads to enhanced cytokine production [51, 52]. Compelling evidence supports the independent roles of TL1A and IL-18 in thymus development; however, their synergistic effect and impact on thymic granulopoiesis remain unexplored. In this study, we examined the influence of TL1A and IL-18 on thymic development and thymic granulopoiesis. Collectively, our data revealed the synergistic roles of TL1A and IL-18 in thymic development, promoting thymic granulopoiesis while limiting T-lymphopoiesis.

## Results

### TL1A and IL-18 synergistically induce neonatal acute thymic atrophy and ex vivo neutrophil expansion

**Fig. 1:**
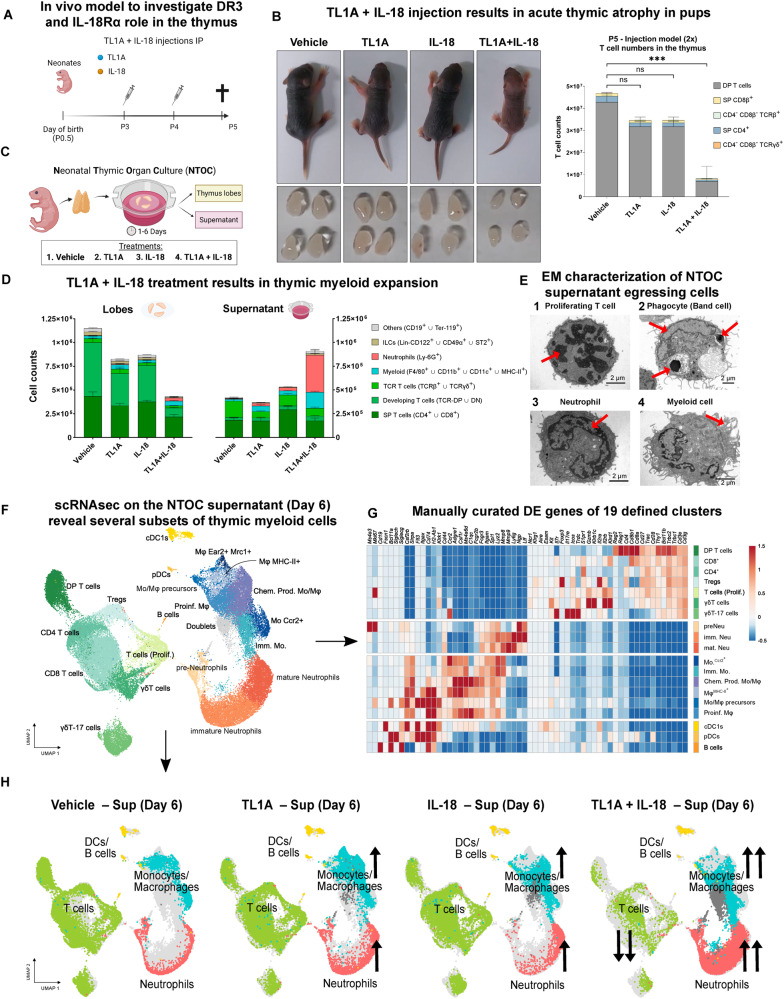
TL1A and IL-18 synergistically induce neonatal acute thymic atrophy and ex vivo neutrophil expansion. **A** Schematic of the cytokine injection model. P3 neonatal pups were IP injected with: (1) PBS (vehicle), (2) IL-18 [100 ng/ml], (3) TL1A [250 ng/ml], or (4) TL1A + IL-18 for two consecutive days in a volume of 20 µl. **B** Effect of TL1A and IL-18 on neonates (P3). Top-left panel: sizes of the neonates (P5) after two injections of the different cytokine treatments. Bottom-left panel: corresponding sizes of the neonatal thymuses. Right panel: quantification of thymic T-cell counts. (*n* = 3). The data are representative of one of at least five independent experiments. The error bars represent the SDs. **C** Schematic of neonatal thymic organ culture (NTOC). Thymuses from neonatal wild-type mice (P0.5) were isolated and cultured in the presence of (1) PBS (vehicle), (2) IL-18 [40 ng/ml], (3) TL1A [100 ng/ml], or (4) TL1A + IL-18. The cells remaining in the thymus lobes or egressing into the supernatant were analyzed by flow cytometry. **D** Flow cytometric analysis of neonatal thymuses and egressing cells after 6 days of culture. The cellular compositions of the thymic lobes (left panel) and supernatant (right panel) are shown. Lin^-^ was defined as CD4^-^CD8β^-^CD3ε^-^TCRβ^-^TCRδγ^-^CD19^-^Ter-119^-^CD11b^-^Ly-6G^-^CD11c^-^F4/80^-^MHC-II^-^. (*n* = 4). The data are representative of one of at least ten independent experiments. Error bars represent the SEM. **E** Transmission electron microscopy (TEM) analysis of cells sorted from the supernatants of NCOCs on Day 6. (**1**) Proliferating T cells (chromatin condensation is highlighted by arrows). (**2**) Mature neutrophil as characterized by its nuclear morphology. (**3**) Band cell phagocytosing two corpses (indicated by arrows). (**4)** Myeloid cell as defined by its size (8 µm) and its extensive cytoplasm and dendrite-like projections. Image (1) was collected from samples of the NTOC supernatant on Day 6 of treatment with PBS (vehicle), and images (2–4) were collected from samples of the NTOC supernatant on Day 6 treated with TL1A + IL-18. **F** Cellular indexing of transcriptomes and epitopes (CITE-Seq) of sorted cells from the NTOC supernatant on Day 6. UMAP representation of the cellular landscape of thymic-egressing cells in the NTOC supernatant on Day 6 subjected to the different treatments. Unsupervised clustering was performed by Seurat (“Findclusters” function, 1.8 resolution) and subsequently manually curated. T cells were divided into seven clusters and are shown in different shades of green. Monocytes/macrophages were subdivided into seven clusters and are depicted in different shades of blue. Neutrophils were divided into three clusters and are represented in shades of orange. Dendritic cells (DCs), plasmacytoid dendritic cells (pDCs), and B cells are depicted in shades of yellow. **G** Heatmap of the manually curated genes that define the cellular identity of the 20 clusters shown in (**F**). **H** Deconvoluted UMAPs of the four different conditions that compose the aggregate shown in (**G**). The 20 defined clusters were grouped into four metaclusters: T cells (green), monocytes/macrophages (blue), neutrophils (orange/red), and others (DCs and B cells, in yellow). From left to right, we show egressing cells in supernatants treated with vehicle, TL1A, IL-18 or TL1A + IL-18. The black arrows highlight significant changes in the metacluster proportions between treatments. *Statistics*: (**B**) One-way ANOVA, *p < 0.05, **p < 0.01, ***p < 0.001, ****p < 0.0001. scRNA-seq single-cell RNA sequencing, UMAP uniform manifold approximation and projection plot, DP T cells double-positive T cells (CD4 + CD8 + ), T_regs_ regulatory T cells, Prolif. T cells proliferative T cells, Imm. Mo immature monocytes, Mo Ccr2+ *Ccr2*^*+*^ Monocytes, Mϕ Macrophages, Proinf. Mϕ proinflammatory macrophages, Chem. Prod. Mϕ chemokine-producing macrophages, preNeutrophils preneutrophils, imm. Neu immature neutrophils, mat. Neu mature neutrophils, DCs dendritic cells, pDCs plasmacytoid dendritic cells, DE differentially expressed

To unravel the role of TL1A and IL-18 in thymic development, we first characterized the expression profiles of the receptors DR3 and IL-18Rα in the thymus of neonates (P0.5, day of birth) and adult mice (12 weeks old) by flow cytometry (Supplementary Fig. 1a, b, respectively). We also characterized the different subsets of thymocytes by flow cytometry, as shown in Supplementary Fig. 1c. While DR3 was widely expressed in both neonatal and adult thymuses (Supplementary Fig. 1a), IL-18Rα expression was mostly restricted to ILC1s, ILC2s, and γδT cells (Supplementary Fig. 1b). Consequently, we identified ILC1s, ILC2s and γδT cells as the primary subsets coexpressing DR3 and IL-18Rα, with ILC1s and γδT cells conforming to a greater proportion in the neonatal thymus than in the adult thymus (Supplementary Fig. 1d–e). Therefore, we decided to examine the impact of DR3 and IL-18Rα engagement in the thymus of neonatal mice. Combined administration of TL1A + IL-18 (Fig. 1A) resulted in acute thymic atrophy and growth retardation, compared to the individual treatments (Fig. 1B), suggesting a synergistic effect. The acute thymic atrophy was characterized by a reduced number of T cells in the combined treatment group (Fig. 1B, right panel).

To assess the localized effect of these cytokine combinations, we established neonatal thymic organ cultures (NTOCs), a three-dimensional ex vivo model system for studying thymus development (schematized in Fig. 1C). After 6 days of TL1A + IL-18 treatment, we analyzed the remaining cellular subsets in the thymic lobes (Fig. 1D, left panel) and egressing cells in the supernatant (Fig. 1D, right panel) by flow cytometry. In accordance with the in vivo results, the effect of single treatments with TL1A and IL-18 was limited, while the combination of TL1A + IL-18 resulted in a profound loss of T cells, reflecting thymic atrophy. Conversely, TL1A + IL-18 treatment induced the expansion of myeloid cells (light blue, CD11b^+^ or F4/80^+^ or CD11c^+^ or MHC-II^+^) and neutrophils (light red, Ly-6G^+^) (Fig. 1D) in the supernatant. The identity of the thymic-egressing cells from the NTOC supernatant was further validated by TEM (Fig. 1E). Additionally, when we examined the NTOC lobes using transmission electron microscopy (TEM), we observed marked morphological differences in the medulla corresponding to the TL1A + IL-18-induced thymic atrophy described by flow cytometry. In the control group, the medulla displayed a greater number of thymocytes, which were smaller in size and closely packed. In contrast, the TL1A + IL-18-treated lobes exhibited larger thymocytes undergoing cell death, accompanied by alterations in the extracellular matrix (Supplementary Fig. 2a).

Thymic neutrophils are largely unexplored compared to their peripheral counterparts. The low frequency of these cells within the thymus, coupled with their short half-life and the limitations of tissue digestion methods for neutrophil retrieval, pose challenges for studying tissue-resident neutrophils using scRNAseq. Consequently, taking advantage of the ex vivo system, we performed CITE-Seq on the NTOC supernatant cells on Day 6 to further characterize the identity, development, and predicted functions of thymic neutrophils. Unsupervised clustering and subsequent manual curation revealed 20 distinct cell populations represented in a UMAP (Fig. 1F). We identified seven clusters of T cells, seven clusters of monocytes/macrophages, three different clusters of neutrophils, and cDC1s, pDCs and B cells. T cells were identified by their expression levels of *Cd3g*, *Cd3e*, *Cd4*, *Cd8b1*, *Trac* and *Trdc*. Monocytes/macrophages were defined by their expression levels of *Spi1*, *Adgre1*, *Fcgr2b*, *Itgam*, *Ccr2*, *Csf1r*, and *H2-Ab1*. Neutrophil identity was determined by their expression levels of Ly6g, *Ltf*, *Ngp*, and *S100a8*. Clustering and annotation were validated by manually curated DE genes (Fig. 1G) and antibody-derived tags (ADTs) (Supplementary Fig. 3a–b). Ultimately, the deconvoluted UMAPs of the thymic egressing cells in each treatment revealed that the combination of TL1A and IL-18 resulted in greater proportions of monocytes/macrophages and neutrophils compared to T cells (Fig. 1H). Collectively, our findings revealed a synergistic effect between TL1A and IL-18 in triggering acute thymic atrophy in vivo and thymic neutrophil expansion ex vivo.

### In vivo administration of TL1A + IL-18 results in acute thymic atrophy and increased thymic neutrophil numbers in neonatal and adult mice

**Fig. 2:**
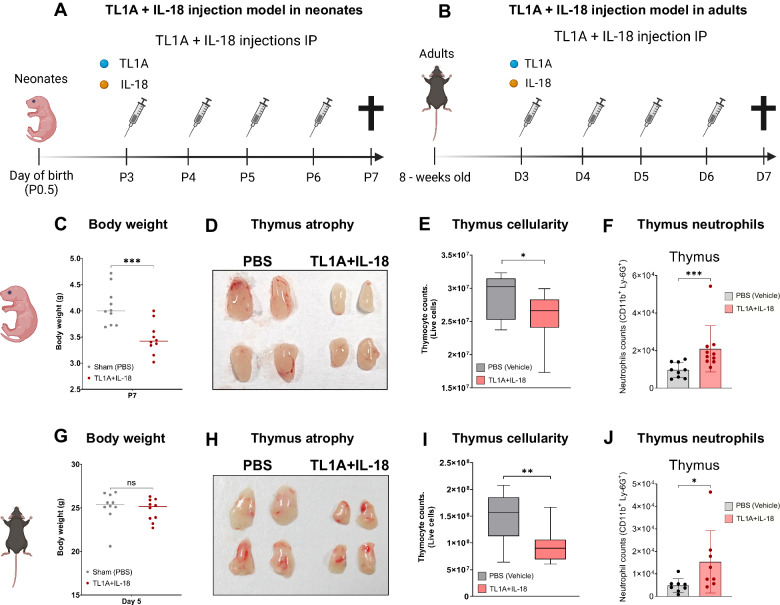
In vivo administration of TL1A + IL-18 results in acute thymic atrophy and increased thymic neutrophil numbers in neonatal and adult mice. **A** Schematic of the experimental model of TL1A + IL-18 injection in neonatal mice. Wild-type (WT) neonatal mice (P3) were IP injected with either PBS (vehicle) or TL1A [250 ng/day] + IL-18 [100 ng/day] for 4 consecutive days in a final volume of 20 µl. **B** Schematic of the experimental model of TL1A + IL-18 injection in adult mice (8 weeks old). WT mice were IP injected with either vehicle (PBS) or TL1A [1 µg/day] + IL-18 [750 ng/day] for 4 consecutive days in a final volume of 200 µl. **C** Body weights of neonates (P7) injected with either PBS (vehicle) or TL1A + IL-18 (*n* = 10). Data representative of one of at least five independent experiments are shown. **D** Pictures of thymuses from neonates (P7) injected with either PBS (vehicle) or TL1A + IL-18. **E** Thymic cellularity of neonates (P7) injected with either PBS (vehicle) or TL1A + IL-18. (*n* = 10). Data representative of one of at least five independent experiments are shown. **F** Quantification of neutrophil numbers in neonatal mice (P7) injected with PBS (gray) or TL1A + IL-18 (blue). Neutrophils were defined as Lin^-^CD11b^+^Ly-6G^+^ cells. The number of neutrophils in the complete thymus was quantified (*n* = 10). Data are representative of one of at least five independent experiments. **G** Body weights of adult model mice on Day 5 (D5) that were injected with either PBS (vehicle) or TL1A + IL-18 (*n* = 10). Data representative of one of at least five independent experiments are shown. **H** Pictures of thymuses from adults (D5) injected with either PBS (vehicle) or TL1A + IL-18. **I** Thymic cellularity of adult thymuses (D5) injected with either PBS (vehicle) or TL1A + IL-18 (*n* = 10). Data representative of one of at least five independent experiments are shown. **J** Quantification of the neutrophil numbers in adult mice (D5) injected with PBS (gray) or TL1A + IL-18 (blue). Neutrophils were defined as Lin^-^CD11b^+^Ly-6G^+^ cells. Neutrophil numbers were quantified from complete thymuses (*n* = 8). Data representative of one of at least five independent experiments are shown. *Statistics*: (**C**, **E**, **G**, **I**) Unpaired t test with Welch’s correction. **E**–**J** The error bars represent the SDs. **F**–**J** The Mann–Whitney U test was performed, as the standard deviations were different between treatment groups. * *p* < 0.05, ** *p* < 0.01, *** *p* < 0.001, **** *p* < 0.0001

Subsequently, we wondered whether the combined influence of TL1A and IL-18 would lead to the expansion of thymic neutrophils in vivo and whether this phenomenon would also occur in adult mice. Therefore, we  designed injection schedules of TL1A + IL-18 into both neonatal (Fig. 2A), and adult mice (Fig. 2B). The mice received injections on four consecutive days to maintain systemic inflammation and replicate the timing of our ex vivo model. The individual administration of TL1A and/or IL-18 did not have major effects on body weight, thymus size, or cellularity in neonatal mice (Supplementary Fig. 4A–C). Nevertheless, neonates injected with TL1A + IL-18 exhibited significant growth retardation (Fig. 2C), while the body weight of adults injected with TL1A + IL-18 was unaffected (Fig. 2G). Both neonates and adults injected with TL1A + IL-18 displayed acute thymic atrophy (Fig. 2D, H), characterized by decreased thymic cellularity (Fig. 2E, I) and a blockade of T-cell development at the DN4 (neonate) and DP (adult) stages (Supplementary Fig. 4h, i). Moreover, the number of thymic neutrophils increased in the TL1A + IL-18-injected neonates and adults (Fig. 2F, J). Additionally, both adult and neonatal mice exhibited splenomegaly (Supplementary Fig. 4d–g), indicating that TL1A + IL-18 treatment induced systemic inflammation. Furthermore, adult mice displayed acute thymus atrophy (Fig. 2H–I), but their body weights remained unchanged (Fig. 2G). This finding underscores that acute thymus atrophy is a specific event rather than a reflection of weight loss. In addition, we explored the effects of TL1A + IL-18 in peripheral organs. Our data convey that these cytokines can induce emergency granulopoiesis, as indicated by the increased neutrophil numbers in the bone marrow, blood, spleen, and lungs (Supplementary Fig. 5a–h). Taken together, these findings revealed that TL1A and IL-18 synergize in vivo and are able to trigger thymic atrophy, increased thymic neutrophil numbers, and systemic neutrophilia in neonatal and adult mice, supporting our ex vivo findings.

### Thymic neutrophils develop and mature in situ in the neonatal thymic organ culture, and NOTCH restricts their development

**Fig. 3:**
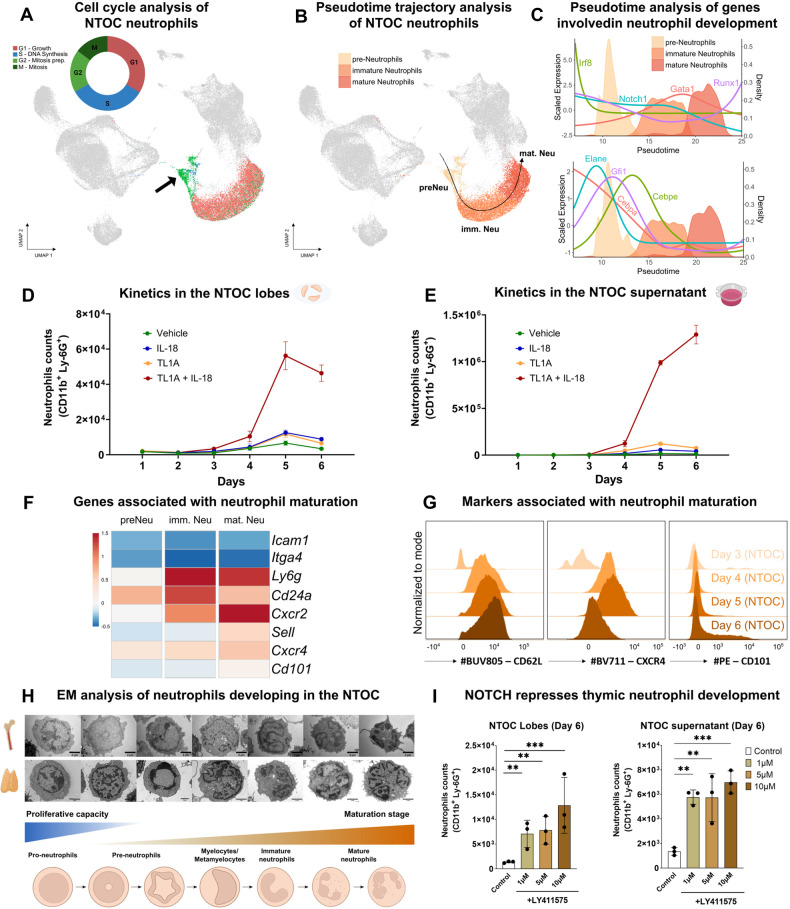
Thymic neutrophils develop and mature in situ in the neonatal thymic organ culture, and NOTCH restricts their development. **A** UMAP representation of scRNAseq cell cycle analysis of the three neutrophil clusters. Phase G2/M cells are shown in green. Phase G1 cells are shown in red. Phase S cells are shown in blue. The black arrow highlights neutrophils in phase G2/M (undergoing mitosis), defined as “pre-Neutrophils” (preNeu) in Fig. 1F. **B** Slingshot trajectory analysis of thymic neutrophils in organ culture. A trajectory from *preNeu → imm. Neu → mat. Neu* was identified. **C** Pseudotime analysis (“Slingshot” package) of genes and transcription factors involved in thymic neutrophil development. Kinetics of neutrophil numbers in the NTOC lobes (**D**) and supernatant (**E**) during the 6 days of culture with different cytokine treatments. (*n* = 3). Data representative of one of at least three independent experiments are shown. Error bars represent the SEM. **F** Heatmap of genes associated with neutrophil maturation. **G** Flow cytometry characterization of the expression of CD62L, CXCR4, and CD101 in newly generated neutrophils in the NTOC lobes from Day 3 to Day 6 after treatment with TL1A + IL-18. Histograms were set in modal mode. (*n* = 3). Data representative of one of at least three independent experiments are shown. **H** EM images from magnetically isolated neonatal bone marrow neutrophils and sorted Ly-6G^+^ cell NTOC supernatant treated with TL1A + IL-18 on Day 6. The different nuclear morphologies reveal distinct maturation stages of neutrophil development. **I** NOTCH negatively regulates neutrophil development in the thymus. NTOCs were treated with (1) control (PBS), (2) 1 µM, (3) 5 µM, or (4) 10 µM of the γ-secretase inhibitor LY411575, which prevents NOTCH signaling activation. (*n* = 3). Data representative of one out of three independent experiments are shown. The error bars represent the SDs. *Statistics*: (**I**) One-way ANOVA, * *p* < 0.05, ** *p* < 0.01, *** *p* < 0.001, **** *p* < 0.0001. TEM transmission electron microscopy

The NTOC assay provides an isolated system to study thymic development, excluding the influx of circulating immune cells and bone marrow progenitors. The expansion of thymic neutrophils under such conditions prompted us to further investigate thymic neutrophil development in an NTOC setting. The three clusters of NTOC-derived neutrophils were annotated as preneutrophils (preNeu), immature neutrophils (imm.Neu), and mature neutrophils (mat.Neu) based on a similar gene profile annotation as shown by Edvard et al. [53]. Cell cycle analysis by scRNAseq illustrated a group of preneutrophils in the G2/M phase (undergoing mitosis) (Fig. 3A), suggesting in situ proliferation of thymic preneutrophils. Unbiased trajectory inference analysis (slingshot) projected a developmental trajectory from preneutrophils (preNeu) to immature neutrophils (imm.Neu) and finally to mature neutrophils (mat.Neu) (Fig. 3B). Furthermore, we identified the DE genes between the three different developmental stages of thymic neutrophils (Supplementary Fig. 6a) as well as the changes in gene expression between the control group (vehicle) and the TL1A + IL-18 treatment group within the neutrophil subsets (Supplementary Fig. 6b). We plotted the expression profiles of transcription factors involved in neutrophil development [53] as a function of trajectory-based pseudotime (Fig. 3C and Supplementary Fig. 6c). The expression levels of *Cebpa* and *Irf8*, which are known to play a role in neutrophil versus monocyte fate decisions [54, 55], were decreased during the early stages of thymic neutrophil development. Additionally, *Elane*, *Gfi1*, *Ms4a3* and *Cepbe* were expressed in preneutrophils, and their expression levels decreased gradually in immature neutrophils. *Notch1* expression decreased as the neutrophils matured, whereas *Gata1* and *Runx1* were upregulated at the mature neutrophil stage. In addition, our scRNAseq analysis revealed that TL1A + IL-18 treatment led to the downregulation of *Bcl11b* and *Tcf7* in developing T cells while upregulating *Spi1* and *Cepba* in myeloid cells, reflecting a shift toward enhanced myeloid production (Supplementary Fig. 6d). We then examined the dynamics of neutrophil expansion in the NTOC culture over the entire duration of the experiment (Days 1–6). In the thymic lobes, the neutrophil count reached its peak on Day 5 of culture (5.6 × 10^4^ cells), with expansion starting on Day 3 (Fig. 3D). In the supernatant, the release and expansion of neutrophils started on approximately Days 3–4, reaching 1.3 × 10^6^ neutrophils on Day 6 (Fig. 3E). Notably, we observed a 23-fold increase in neutrophil counts between the supernatant and the thymic lobes, suggesting that further expansion occurred after egression. Figure 3F displays a heatmap of the expression levels of genes related to neutrophil maturation. *Cxcr2*, *Sell*, *Cxcr4* and *Cd101* expression levels increased as neutrophils matured, in line with previous reports [56, 57]. We validated our scRNseq data at the protein level by flow cytometry (Fig. 3G). On Day 3, the majority of the neutrophils were CD11b^+^Ly-6G^+^CXCR4^-^CD62L^-^. On Day 4, they became CXCR4^+^CD62L^+^, later acquiring a CD11b^+^Ly-6G^+^CXCR4^high^CD62L^high^CD101^-^ phenotype by Day 5. Finally, on Day 6, the expression of CD62L and CXCR4 decreased, and part of the population became positive for CD101, a marker of neutrophil maturation [53]. Electron microscopy analysis of bone marrow-, and NTOC-derived neutrophils revealed an array of different developmental stages of neutrophils according to nuclear morphology, starting from pro-neutrophils, preneutrophils, myelocyte-like neutrophils, immature neutrophils, and finally mature neutrophils, which correspond with the recently proposed neutrophil differentiation pathway [58] (Fig. 3H). This finding reinforced our initial hypothesis that neutrophils can develop and mature locally in the thymus. In view of the decreased expression of *Notch1* during neutrophil maturation (Fig. 3C), we investigated the role of NOTCH in thymic neutrophil development by blocking a γ-secretase required for activation of downstream signaling of NOTCH using the inhibitor LY411575. Neutrophil counts were increased upon treatment with LY411575, indicating that NOTCH signaling represses neutrophil development (Fig. 3I), which is in line with studies suggesting that NOTCH inhibits myeloid fate [10, 59, 60].

### *Rag1*-Cre and *Ms4a3*-Cre fate mapping reveals that thymic neutrophils emerge from *Rag1*^+^ thymic GMPs

**Fig. 4:**
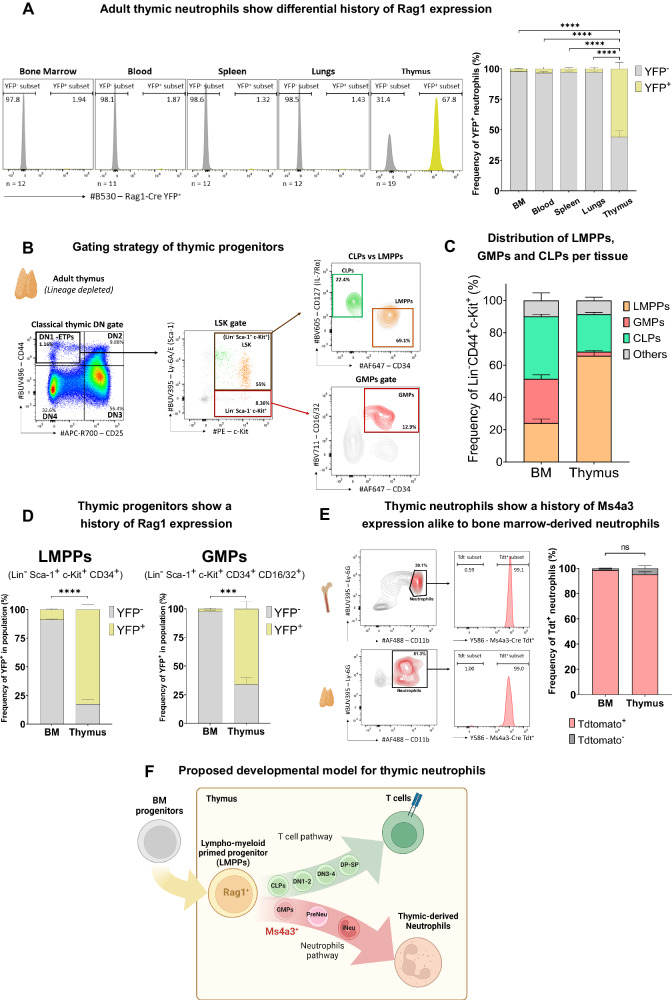
*Rag1*-Cre and *Ms4a3*-Cre fate mapping reveals that thymic neutrophils emerge from *Rag1*^**+**^ thymic GMPs. **A** Characterization of the history of *Rag1* expression in adult (8-week-old) neutrophils (defined as Ly-6G^+^ CD11b^+^) in the bone marrow, blood, spleen, lung, and thymus using the *Rag1*-Cre genetic model (Supplementary Fig. 7a). The YFP^-^ fraction is shown in gray, and the YFP^+^ fraction is shown in yellow. The quantification of the percentage of *Rag1*-Cre YFP^+^ neutrophils across organs is shown on the right (n, indicated in the figure. *n* = 19 for thymus, *n* = 12 for bone marrow, spleen and lungs, *n* = 11 for blood). The data were pooled from five independent experiments. **B** Flow cytometric characterization of the different progenitor populations found in the adult thymus. Thymuses from 8-week-old *Rag*1-Cre^+/tg^ Rosa26YFP^+/tg^ mice were isolated and subjected to negative bead depletion to enrich Lin^-^ cells. The gating of double-negative thymocytes (DNs) is shown on the left. DN1 (Lin^-^CD44^+^CD25^-^), DN2 (Lin^-^CD44^+^CD25^+^), DN3 (Lin^-^CD44^-^CD25^+^), and DN4 (Lin^-^CD44^-^CD25^-^). Thereafter, DN1s were further dissected, and by using Ly-6A/E (Sca-1) and c-Kit (CD117), we defined the progenitors deeper into the LSK gate (Lin^-^ Sca-1^+^ and c-Kit^+^, in brown) and the Lin^-^c-kit^+^Sca-1^-^ progenitors. LSK cells were divided into common lymphoid progenitors (CLPs), defined as Lin^-^CD44^+^Sca-1^+^c-Kit^+^IL-7Rα^+^CD34^-^ (in green), and lympho-myeloid primed progenitors (LMPPs), defined as Lin^-^CD44^+^Sca-1^+^c-Kit^+^IL-7Rα^low/-^CD34^+^ (in orange). Granulocyte–monocyte progenitors (GMPs) were defined as Lin^-^Sca-1^-^c-Kit^+^CD16/32^+^CD34^+^ (in red), as described previously [55]. Progenitors in the bone marrow were defined with the same gating strategy, as indicated in Supplementary Fig. 7c. Lin^-^ is defined as CD19^-^Ter-119^-^CD11c^-^CD11b^-^MHC-II^-^F4/80^-^Ly-6G^-^CD4^-^CD8b^-^TCRβ^-^TCRγδ^-^CD25^-^. **C** Frequencies of LMPPs, CLPs, and GMPs within the Lin^-^c-Kit^+^ fraction in the thymus and bone marrow of adults. (*n* = 3). Data representative of one of three independent experiments are shown. **D** Characterization of the history of *Rag*1 expression in adult thymic LMPPs (left plot) and GMPs (right plot). Lin^-^ was defined as CD19^-^Ter-119^-^CD11c^-^CD11b^-^MHC-II^-^F4/80^-^Ly-6G^-^CD4^-^CD8b^-^TCRβ^-^TCRγδ^-^CD25^-^. (*n* = 3). Data representative of one of three independent experiments are shown. **E** Fate-mapping characterization of the expression of *Ms4a3*-Cre in adult thymic and bone marrow neutrophils. Neutrophils (shown in red) were gated as Ly-6G^+^CD11b^+^ cells. (*n* = 8). Data representative of one of two independent experiments are shown. **F** Schematic of the proposed developmental model for thymic neutrophils. *Statistics*: (**A**, **C**, **D**, **E**) Error bars represent SEM. **A** One-way ANOVA; **D** unpaired t test with Welch correction. * *p* < 0.05, ** *p* < 0.01, *** *p* < 0.001, **** *p* < 0.0001. BM bone marrow, CLPs common lymphoid progenitors, LMPPs lymphoid-primed progenitors, GMPs granulocyte-monocyte progenitors

Thymic neutrophils can originate from thymic precursors [14]. Nonetheless, the exact identity of these thymic precursors and the specific thymic extramedullary developmental pathway compared to that of bone marrow-derived neutrophils have not yet been fully elucidated. Therefore, we addressed these questions with state-of-the-art fate-mapping tools by using *Rag1*-Cre Rosa26-YFP (Supplementary Fig. 7a) and *Ms4a3*-Cre Rosa26-TdtTomato [61] mice, in which both lymphoid and myeloid fates are traced. First, we investigated the ability of *Rag1*-Cre to label neutrophils in the bone marrow, blood, spleen, lungs, and thymus of adult mice (Fig. 4A). Our data revealed differential *Rag1* expression in the thymus (~60% of thymic neutrophils YFP^+^, ****, *p* < 0.0001, *n* = 19) compared to the expression levels in the bone marrow (2.5%), blood (3.5%), spleen (3%), and lungs (2.9%). These data indicate that a large fraction of thymic neutrophils originate from thymic progenitors. In contrast, thymic macrophages were mostly unlabeled *(*5.4%), suggesting a different developmental pathway from that of thymic neutrophils (Supplementary Fig. 7b). This finding confirms previous reports suggesting distinct origins for thymic macrophages and T cells based on *Il7r*-Cre [15] and *Cx3cr1*-Cre-ER^T2^ [24] fate mapping models. Second, we examined the different progenitor subsets in the thymus and bone marrow. Three subsets of progenitors were classified as common lymphoid progenitors, CLPs (in light green, defined as Lin^-^CD44^+^CD25^-^Sca-1^+^c-Kit^+^IL-7Rα^+^CD34^-^), lymphoid-myeloid primed progenitors, LMPPs (in orange, defined as Lin^-^ CD44^+^ CD25^-^ Sca-1^+^ c-Kit^+^ IL-7Rα^low/-^ CD34^+^) and granulocyte-monocyte progenitors, GMPs (in red, defined as Lin^-^ CD44^+^ CD25^-^ Sca-1^-^c-Kit^+^ CD16/32^+^ CD34^+^) (Fig. 4B), using a gating strategy similar to that previously described [19, 55]. The same populations were defined in the bone marrow using identical gating methods (Supplementary Fig. 7c). Next, we quantified the proportions of these different progenitors within the Lin^-^ CD44^+^ c-Kit^+^ fraction in both the thymus and bone marrow (Fig. 4C). While nearly one-third of the Lin^-^ CD44^+^ c-Kit^+^ bone marrow progenitors were GMPs (27.3%), only 2.4% of the Lin^-^ CD44^+^ c-Kit^+^ thymic progenitors were identified as GMPs. We then examined the expression history of *Rag1* in both bone marrow and thymic progenitors to discern the differences between medullary and thymic neutrophil development. We reported that 82.7% of thymic LMPPs showed a history of *Rag1* expression, while only 9% of bone marrow LMPPs were labeled with *Rag1*-Cre. Remarkably, ~66.7% of thymic GMPs exhibited *Rag1*-Cre labeling, in contrast to bone marrow GMPs, where the *Rag*1 expression history was minimal (2.2%) (Fig. 4D). Although *Rag1* expression is linked to VDJ rearrangement and subsequent TCR assembly, some studies have reported the expression of *Rag1* [62] and VDJ rearrangements [16] in ETPs prior to T-cell commitment. To confirm these findings, we analyzed publicly available transposase-accessible chromatin with sequencing (ATAC-Seq) data on ETPs [63]. Our analysis revealed an open chromatin region at the transcription start site (TSS) (chromosome 2, position 101, 470, 448) in the *Rag1* locus (Supplementary Fig. 6d), confirming that *Rag1* can be expressed prior to T-cell commitment in ETPs. Finally, we used the *Ms4a3*-Cre fate-mapping model, which labels cells that adopt a myeloid fate from the GMP state onward [61]. We confirmed that *Ms4a3*-Cre expression was restricted exclusively to myeloid cells in the thymus, as expected (Supplementary Fig. 7e). We did not observe significant differences in *Ms4a3*-cre labeling between thymic (98.6% labeled) and bone marrow (99.9% labeled) neutrophils (Fig. 4E). Hence, thymic neutrophils likely arose from thymic GMP progenitors that were lymphoid-primed and had a history of *Rag1* expression prior to *Ms4a3* expression (schematized in Fig. 4f).

### Thymic neutrophils can phagocytose, produce ROS, migrate, and form NETs, similar to benchmark peritoneal neutrophils

**Fig. 5:**
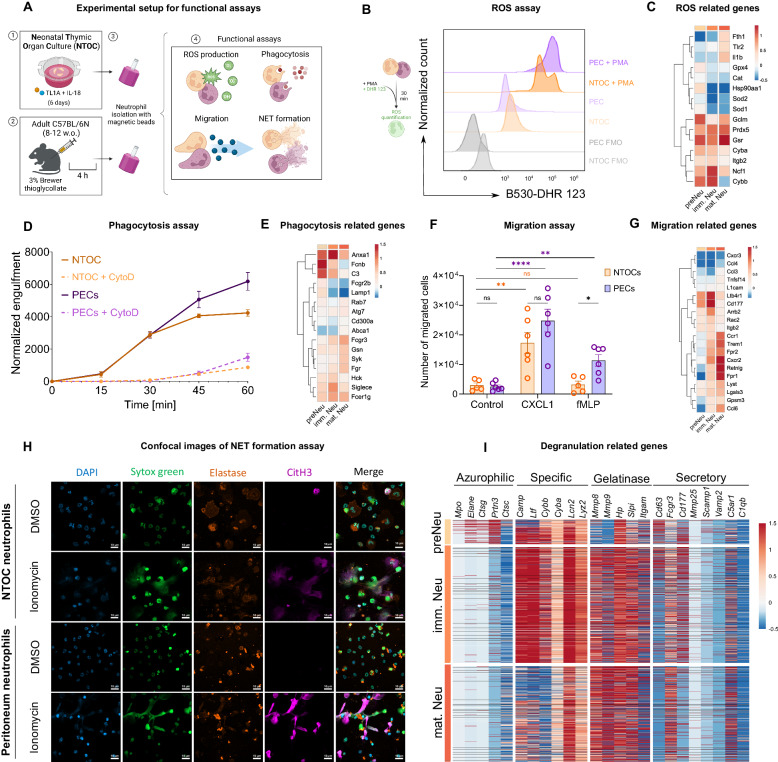
Thymic neutrophils can phagocytose, produce ROS, migrate, and form NETs similar to benchmark peritoneal neutrophils. **A** Schematic of the experimental design used to assess the ROS production capacity, phagocytosis, migration, and NET formation of thymic neutrophils compared to those of peritoneal neutrophils. Thymic neutrophils were isolated from NTOC supernatants treated for 6 days with TL1A + IL-18. Adult peritoneal neutrophils were isolated 4 h after IP injection of 1 ml of 3% Brewer thioglycolate. Neutrophils were purified using an EasySep™ Mouse Neutrophil Enrichment Kit. Isolated peritoneal and thymic neutrophils were treated with PBS, eBioscience™ Cell Stimulation Cocktail (1:500), DHR 123 [5 µM], pHrodo particles from *S. aureus* [1 mg/mL], cytochalastin D [2 µM], DMSO, LPS [4 µg/ml] from *Klebsiella pneumoniae*, and ionomycin [2.5 µg/ml]. **B** ROS production assay. Both TL1A- and IL-18-induced NTOC-derived and peritoneal neutrophils were treated with DHR 123 [5 µM] at 37 °C and 5% CO_2_ for 30 min. The fluorescence intensities were measured in channel B530 and are displayed as histograms. The Y-axes are normalized to the mode. Data representative of one of two experiments are shown. **C** Heatmap of manually curated neutrophil ROS-related genes. **D** Phagocytosis assay. TL1A + IL-18-induced, NTOC-derived, and peritoneal neutrophils were incubated with PE-conjugated pHrodo particles from *S. aureus* for 60 min and treated with cytochalastin D [2 µM] to inhibit phagocytosis. The number of engulfment events was normalized to the cell count. Data representative of one of two experiments are shown. **E** Heatmap of manually curated neutrophil phagocytosis genes. **F** Migration assay. TL1A + IL-18-induced, NTOC-derived, and peritoneal neutrophils were seeded at 150,000 cells/well on top of a 12-well Transwell plate with 5.0 µm pores and incubated with the neutrophil chemoattractants CXCL1 [50 ng/mL] and fMLP [10 µM] at 37 °C and 5% CO_2_ for 90 min. The migrated cells were counted in a BD FACSVerse™ Cell Analyzer. Data representative of one of two experiments are shown. **G** Heatmap of manually curated neutrophil migration-related genes. **H** Confocal images of thymic neutrophils isolated from the supernatants of TL1A + IL-18-stimulated NCTs (Day 6) and treated with DMSO (vehicle) or ionomycin for 4 h. The cells were stained with DAPI, SYTOX Green, and antibodies for detecting neutrophil elastase and citrullinated histone 3 (citH3). The scale bar represents 10 µM. (*n* = 3). Data representative of one of three independent experiments are shown. **I** Heatmap representation of the genes associated with primary/azurophilic, secondary/specific, tertiary/gelatinase or secretory granule release by NTOC-derived neutrophils. *Statistics*: (**F**) One-way ANOVA, * *p* < 0.05, ** *p* < 0.01, *** *p* < 0.001, **** *p* < 0.0001. **C**, **E**, **G** The Y-axes were subjected to hierarchical clustering. NET Neutrophil extracellular trap

As the most abundant leukocytes in humans and mice, neutrophils migrate to inflammatory sites, where they engage in phagocytosis, secrete antimicrobial products and reactive oxygen species (ROS), form neutrophil extracellular traps (NETs), and play a crucial role in tissue remodeling [41, 64]. We compared the functionalities of newly generated thymic neutrophils to those of isolated benchmark peritoneal neutrophils, as shown in the schematic in Fig. 5a. Flow cytometric staining with dihydrorhodamine 123 (DHR 123) revealed that NTOC-derived and peritoneal-derived neutrophils can similarly mount an oxidative burst, as they both produce high amounts of ROS with and without PMA stimulation (Fig. 5B). Interestingly, the NTOC population displayed a biphasic DHR 123 histogram in response to PMA, with one of the two peaks at the same level as that of peritoneal neutrophils and the other revealing a lower capacity for ROS production. The observed biphasic histogram might reflect the heterogeneity of the NTOC population at different stages of neutrophil development. Next, we analyzed the expression profiles of ROS-related genes in the three clusters of NTOC-derived neutrophils (Fig. 5C). We observed high expression of ferritin (*Fth1*), peroxiredoxin (*Prdx5*), glutathione reductase (*Gsr*) and neutrophil cytosolic factor (*Ncf1*) (among others), which are known to be key in metabolic processes linked to ROS production and are critical for microbicidal activity. Additionally, NTOC-derived neutrophils exhibited a similar capacity to phagocytose pHrodo particles from *S. aureus* (Fig. 5D and Supplementary Fig. 8a). scRNAseq analysis confirmed the high expression of phagocytosis-related genes, such as *Syk*, *Fcgr3*, and *Gsn* (Fig. 5E).

We then evaluated the migratory capacities of NTOC-derived and peritoneal neutrophils. Our data showed that both NTOCs and peritoneal neutrophils responded similarly to CXCL1, while only peritoneal neutrophils were chemoattracted by fMLP at this concentration (Fig. 5F). At the gene level, thymus neutrophils highly expressed essential chemokine receptors and integrins that orchestrate neutrophil migration, such as *Cxcr2* (receptor for CXCL1), *Fpr1*, *Fpr2* (receptors for fMLP), *Ltb4ar1*, and *Ccr1* (Fig. 5G). Furthermore, the confocal images shown in Fig. 5h illustrate the similar NET formation capacities of NTOCs and peritoneum neutrophils, as quantified in Supplementary Fig. 8b, c. Finally, we further analyzed the expression profiles of genes associated with the release of azurophilic-, specific-, gelatinase-, and secreted protein-containing granules (Fig. 5I). Preneutrophils showed gene expression associated with azurophilic components such as cathepsins, while immature neutrophils expressed genes linked to higher contents of secondary/specific granules such as lactotransferrin (*Ltf*). Mature neutrophils expressed genes associated with tertiary/gelatinase granules with high levels of metalloproteases (Fig. 5A and Supplementary Fig. 8d, e), which are known to be involved in tissue remodeling [62]. Taken together, our results demonstrate that TL1A + IL-18-induced thymic neutrophils behave similarly to peritoneal neutrophils and can perform functions such as ROS production, phagocytosis, migration, and NET formation.

### GM-CSF-dependent thymic neutrophil expansion

**Fig. 6:**
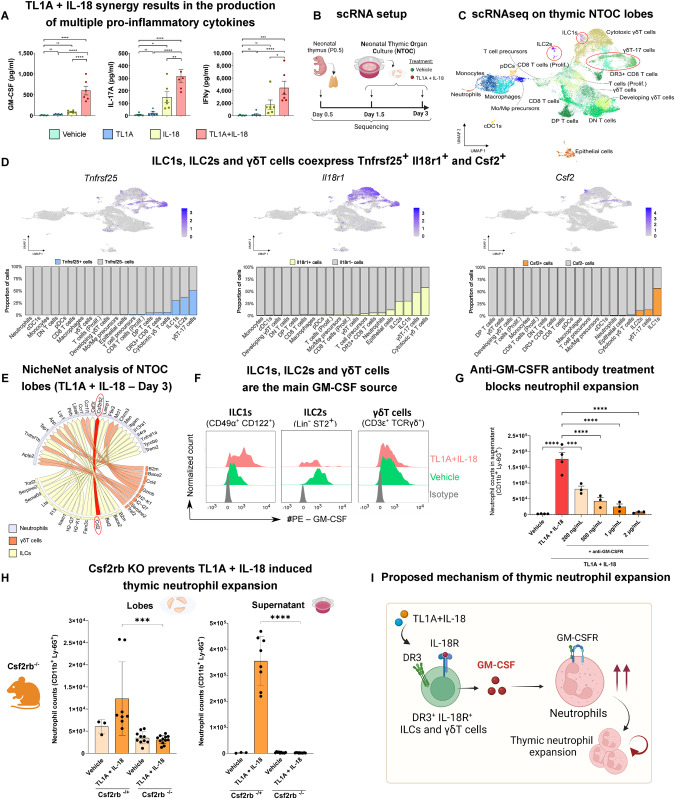
Thymic neutrophil expansion is dependent on GM-CSF. **A** Multiplex cytokine levels in NTOC supernatants of thymic lobes on Day 6 after treatment with (1) vehicle (green), (2) TL1A (blue), (3) IL-18 (yellow), or (4) TL1A + IL-18 (red). From left to right, we measured GM-CSF, IL-17A and IFN-γ in NTOC supernatants (*n* = 6). The results shown are combined from three independent experiments. Error bars represent the SEM. **B** Schematic of the experimental design used for scRNA-seq analysis of the neonatal thymic lobes during the early phase of culture (Days 0–3). We collected (1) neonatal thymuses from wild-type mice on the day of birth (P0.5), (2) thymic lobes cultured in the NTOC for 1.5 days treated with PBS (vehicle), (3) thymic lobes cultured in the NTOC for 1.5 days treated with TL1A + IL-18, (4) thymic lobes cultured in the NTOC for 3 days treated with PBS (vehicle), and (6) thymic lobes cultured in the NTOC for 3 days treated with TL1A + IL-18 (see Supplementary Fig. 9b). The samples were further processed as described in the Supplementary methods. **C** Convoluted UMAP of the aggregates of all the samples: (1) neonatal thymus (P0.5), (2) NTOC vehicle - Day 1.5, (3) NTOC TL1A + IL-18 - Day 1.5, (4) NTOC vehicle - Day 3, and (5) NTOC TL1A + IL-18 - Day 3. Unsupervised clustering was performed by Seurat (“Findclusters” function, 1.8 resolution) and subsequently manually curated and grouped into 19 clusters. **D** Feature plots of the gene expression levels of *Tnfrsf25* (encoding DR3), *Il18r1* (encoding IL-18Rα), and *Csf2* (encoding GM-CSF) and corresponding stacked-bar plots with the quantified percentages of *Tnfrsf25-*, *Il18r1-*, *and Csf2-*expressing cells across clusters. **E** NicheNet analysis of the predicted ligand‒receptor pair interactions between γδ T cells and both ILCs (sender cells) and neutrophils (receivers). The predicted interaction between *Csf2* (from ILCs) and *Csf2rb* (neutrophils), one of the top hits, is highlighted in red. **F** Intracellular flow cytometry was performed to identify GM-CSF-producing subsets in the NTOC. Thymic lobes were cultured for 3 days and treated with either (1) vehicle or (2) TL1A + IL-18. We show the GM-CSF production capacity of ILC1s [defined as Lin^-^CD122^+^CD49α^+^], ILC2s [defined as Lin^-^ST2^+^], γδT cells [defined as CD3ε^+^γδTCR^+^] (all populations DR3^+^IL-18Rα^+^, as previously shown) and monocytes/macrophages [defined as CD11b^+^ F4/80^+^] via histograms. (*n* = 4). Data representative of one of seven independent experiments are shown. **G** GM-CSFR antibody blockade in the NTOC supernatant on Day 6 of culture. NTOCs were treated with (1) vehicle (PBS), (2) TL1A + IL-18 (as previously described), (3) TL1A + IL-18+α-GM-CSFR [200 ng/ml], (4) TL1A + IL-18+α-GM-CSFR [500 ng/ml], (5) TL1A + IL-18+α-GM-CSFR [1 µg/ml], or (6) TL1A + IL-18+α-GM-CSFR [2 µg/ml] (*n* = 3). The results shown represent one out of three independent experiments. **H** NTOC culture of thymic lobes from *Csf2rb*^-/-^ mice. The number of expanded neutrophils in response to TL1A + IL-18 treatment versus vehicle (control) treatment was quantified in *Csf2rb*^-/-^ versus *Csf2rb*^-/+^ littermates. (*Csf2rb*^+/-^ mice treated with vehicle, *n* = 3). *Csf2rb*^+/-^ mice treated with TL1A + IL-18, *n* = 8. *Csf2rb*^-/-^ mice treated with vehicle, *n* = 10. *Csf2rb*^+/-^ mice treated with TL1A + IL-18, *n* = 12. The results shown are a combination of two independent experiments. The error bars represent the SDs. **I** Proposed cellular mechanism of thymic neutrophil expansion in response to the synergistic effect of TL1A and IL-18. *Statistics*: (**A**, **G**, **H**) One-way ANOVA, * *p* < 0.05, ** *p* < 0.01, *** *p* < 0.001, **** *p* < 0.0001. Mo/Macs Monocytes/Macrophages

Finally, we wanted to elucidate the underlying mechanism triggered by TL1A + IL-18 treatment that sustains the development and expansion of thymic neutrophils. Measurements of the cytokines released into the NTOC supernatant revealed significant upregulation of GM-CSF, IL-17A, IFNγ (Fig. 6A), IL-5, IL-22 and TNFα and downregulation of IL-10 (Supplementary Fig. 9a) in response to synergistic treatment with TL1A + IL-18. GM-CSF, IL-17A, and IFNγ are known key regulators of reactive granulopoiesis during inflammation [65–67], and IL-22 is known to play a role in thymus regeneration after injury [68]. Our results emphasize the synergistic effect of TL1A with IL-18 in boosting the production of proinflammatory cytokines, similar to previous reports [69]. To gain further insights into the cellular mechanism that supports neutrophil production in response to TL1A and IL-18, we performed scRNA-seq on the thymic lobes at early time points of thymic culture (Fig. 6B). Our results further validated the scRNA-seq data shown in Fig. 1g, confirming increased myelopoiesis in the thymus lobes in culture in response to TL1A and IL-18 treatment (Supplementary Fig. 9b). Unsupervised Seurat clustering revealed 19 different clusters, primarily composed of T cells, myeloid cells, epithelial cells, ILCs and γδT cells (Fig. 6C, and Supplementary Fig. 9b). Their cell identity was further confirmed by plotting the manually curated differentially expressed genes, as shown in Supplementary Fig. 10a. Interestingly, *Tnfrsf25* (encoding DR3), *Il18r1* (encoding IL-18Rα) and *Csf2* (encoding GM-CSF) were mainly restricted and coexpressed in ILCs and γδT cells (Fig. 6D). These results further confirmed our in vivo steady-state characterization of DR3 and IL-18Rα expression, as shown in Supplementary Fig. 1a, b. This expression pattern suggested that γδT cells, ILC1s, and ILC2s may act as possible sentinels of inflammation by producing GM-CSF (*Csf2*) in response to TL1A + IL-18 and consequently enhancing thymic granulopoiesis. Non-biased analysis of the potential ligand‒receptor pair interactions by NicheNet confirmed that *Csf2* (from *Tnfrsf25*^+^ *Il18r1*^+^ ILCs) to *Csf2rb* (neutrophils) was one of the top predicted interactions (Fig. 6E). Additionally, our NicheNet data revealed a shift in the primary predicted interactions between ILCs/γδT cells (sender cells) and neutrophils (receiver cells). In the control condition, ligand*-Notch* interactions were among the top predicted hits. Conversely,  among the top predicted interactions following the treatment with TL1A + IL-18 were *Ifng-Ifngr2* (Days 1 and 5) and *Il13-Il13ra1/Il4ra* (Day 3), supporting the observed shift toward enhanced myelopoiesis (Supplementary Fig. 11a). These findings indicate that TL1A + IL-18 treatment alters the thymic microenvironment, favoring thymic neutrophil development at the expense of T-cell development. This outcome aligns with our cytokine measurements, as presented in Fig. 6a and Supplementary Fig. 9a. Subsequently, to validate the cellular source of enhanced GM-CSF production, we performed intracellular flow cytometry (Supplementary Fig. 12a), where we primarily observed DR3^+^ IL-18Rα^+^ co-expressing ILC1s producing high amounts of GM-CSF in response to treatment with TL1A + IL-18 compared to the control (Fig. 6F). Antibody blockade of GM-CSFR in the NTOC resulted in significant reductions in neutrophil counts in the supernatant (Fig. 6G) and thymic lobes (Supplementary Fig. 12b). To investigate the contribution of γδ-T cells to GM-CSF production, we performed NTOCs with *Tcrd*^-/-^ thymic lobes. However, this did not result in a reduced number of thymic neutrophils in response to TL1A + IL-18 compared to that in the WT lobes (Supplementary Fig. 12c). These results suggested that ILC1s were the major source of GM-CSF in response to TL1A + IL-18. Finally, using thymic lobes from *Csf2rb*^-/-^ mice, we demonstrated that the absence of *Csf2rb* (GM-CSFR) completely blocks TL1A + IL-18-induced thymic neutrophil expansion (Fig. 6H). Thus, GM-CSF is the key factor driving the reprogramming toward thymic emergency granulopoiesis (as schematically shown in Fig. 6I).

### PVM and MCMV infection leads to acute thymus atrophy, enhanced TL1A and IL-18 levels, and an increased proportion of thymic neutrophils

**Fig. 7:**
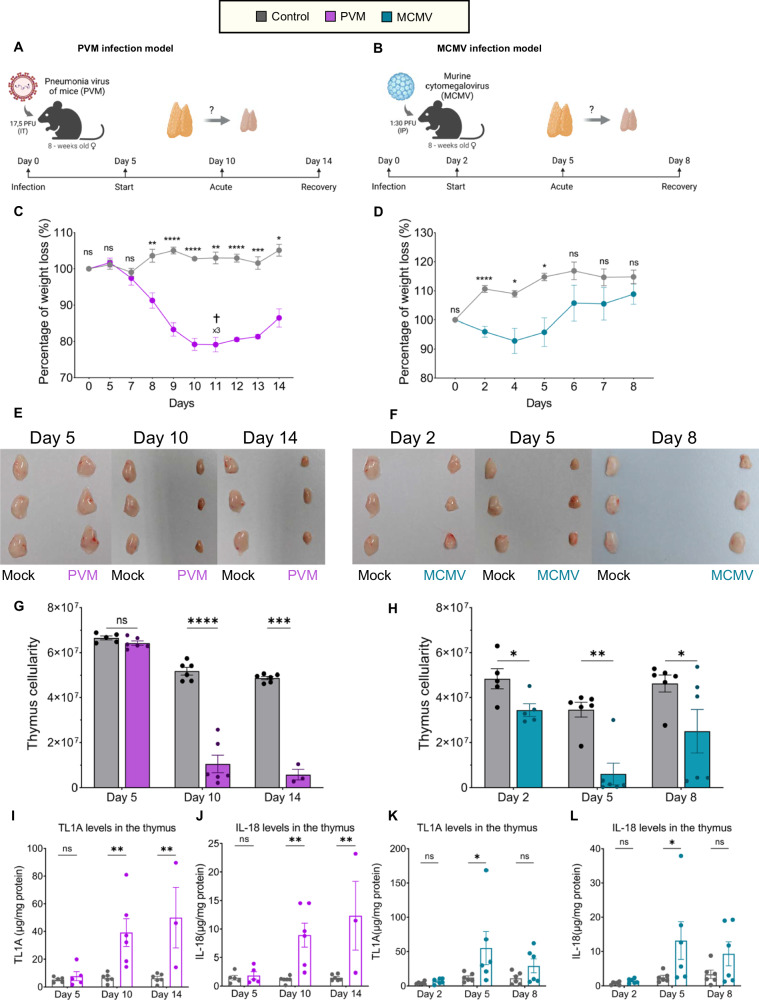
PVM and MCMV infection leads to acute thymus atrophy, TL1A and IL-18 upregulation, and an increased proportion of thymic neutrophils. **A** Schematic of the PVM infection model in adult-, wild-type-, female mice. WT mice were administered with either vehicle (PBS) or 17.5 PFU of PVM intratracheally (IT) in a final volume of 200 µl. Thymus size, cellularity, the expression levels of TL1A and IL-18, and the number of neutrophils were evaluated on Days 5, 10, and 14 after infection. **B** Schematic of the MCMV infection model in adult, wild-type, female mice. WT mice were intraperitoneally (IP) injected with either vehicle (PBS) or 1:30 PFU of MCMV in a final volume of 200 µl. Thymus size, cellularity, the levels of TL1A and IL-18, and the number of neutrophils were evaluated on Days 2, 5, and 8 after infection. **C** Percentages of body weight loss in mock (gray)- and PVM (purple)-infected mice during the course of the experiment. **D** Percentages of body weight loss in mock (gray)- and MCMV (blue)-infected mice during the course of the experiment. **E** Pictures of thymuses from mock-infected (gray) or PVM-infected (purple) mice on Days 5, 10, and 14 after infection. **F** Pictures of thymuses from mock-infected (gray) or MCMV-infected (blue) mice on Days 2, 5, and 8 of infection. **G** Cellularity of thymuses from mock-infected (gray) or PVM-infected (purple) mice on Days 5, 10, and 14 after infection. **H** Cellularity of thymuses from mock-infected (gray) or MCMV-infected (blue) mice on Days 2, 5, and 8 after infection. **I** Quantification of the levels of TL1A normalized to the mg of protein in the thymuses of mock-infected (gray) or PVM-infected (purple) mice at Days 5, 10, and 14 of infection. **J** Quantification of the levels of IL-18 normalized to the mg of protein in the thymuses of mock-infected (gray) or PVM-infected (purple) mice at Days 5, 10, and 14 of infection. **K** Quantification of the levels of TL1A normalized to the mg of protein in the thymuses of mock-infected (gray) or MCMV-infected (blue) mice on Days 2, 5, and 8 of infection. **(L)** Quantification of the levels of IL-18 normalized to the mg of protein in the thymuses of mock-infected (gray) or MCMV-infected (blue) mice on Days 2, 5, and 8 of infection. *Statistics*: (**C**, **D**) Unpaired t test with Welch’s correction. **G**–**L** Two-way ANOVA. * *p* < 0.05, ** *p* < 0.01, *** *p* < 0.001, **** *p* < 0.0001. Data representative of one of two experiments are shown. Error bars represent the SEM

We then infected mice with murine cytomegalovirus (MCMV) and pneumonia virus (PVM) to investigate changes in thymus size, cellularity, neutrophil numbers, and in situ levels of TL1A and IL-18. Here we examined the state of the thymus during the incubation phase (PVM Day 5), or early stages of infection (MCMV Day 2), acute infection (PVM Day 10, MCMV Day 5), and the recovery phase (PVM Day 14, MCMV Day 8), as schematized in Fig. 7A, B. Based on the bodyweight, PVM-infected mice started showing signs of infection after Day 7, which worsened until Day 10, the peak of the infection. The surviving mice (3/6 mice succumbed to the infection on Day 11) slowly recovered afterward (Fig. 7C). The kinetics of MCMV infection were faster than those of PVM infection, as the mice displayed weight loss from Day 2 post infection, disease progression until Days 4–5, and recovery afterward, with no difference in weight loss by Day 8 (Fig. 7D).

Next, we analyzed the state of the thymus at different time points. Compared with control mice, PVM-infected mice exhibited no difference in thymus size or cellularity on Day 5 while showing acute thymus atrophy on Day 10 (infection peak) and Day 14 (recovery phase), as illustrated in Fig. 7e and quantified in Fig. 7g. MCMV-infected mice manifested acute thymus atrophy on Day 2 of infection, which was aggravated on Day 5, while half of the mice showed recoveries in thymus size and cellularity by Day 8 (Fig. 7F, H). We then examined the levels of TL1A and IL-18 in the thymus at different stages of PVM and MCMV infection. We observed reduced protein levels in both PVM- (Days 10 and 14) and MCMV-infected mice (Day 5) during acute thymus atrophy (Supplementary Fig. 13a–b), as expected. PVM-infected mice showed increased relative levels of TL1A and IL-18 during acute thymus atrophy at Days 10 and 14 (Fig. 7I–J), while MCVM-infected mice displayed increased TL1A and IL-18 only during acute thymus atrophy on Day 5, but not during the early infection stage (Day 2), or in the recovery phase (Day 8) (Fig. 7K–L). These results show that viral-induced thymus atrophy results in enhanced availability of TL1A and IL-18 in the thymus.

Finally, we explored the changes in the thymus neutrophil compartment during PVM and MCMV infection. We observed marked increases in the neutrophil proportion in the thymus during acute thymus atrophy in both PVM-infected (Day 10, Supplementary Fig. 13c) and MCMV-infected (Day 5, Supplementary Fig. 13e) mice, while the absolute neutrophil numbers were unchanged or reduced (Supplementary Fig. 13d, f). Notably, we also observed neutrophilia in the blood of PVM- and MCMV-infected mice during the acute phase of infection (Supplementary Fig. 13g–j). Taken together, our results show that PVM and MCMV viral infection results in thymic atrophy, accompanied by increased availability of TL1A and IL-18 and alterations in the thymic neutrophil compartment.

## Discussion

Acute thymic atrophy is a transient state in which T-cell development is impaired [70]. Although defects in lymphopoiesis have been associated with thymic atrophy, the impact of systemic inflammation on thymic granulopoiesis has yet to be explored. In this report, we document that TL1A and IL-18 synergistically induce acute thymic atrophy in vivo and ex vivo, accompanied by thymic neutrophil expansion. TL1A [71] and IL-18 [72] can both be induced upon TLR activation and are elevated in the serum of patients with inflammatory pathologies, such as rheumatoid arthritis (RA) [73, 74] and asthma [74, 75]. In the serum of RA patients, TL1A can reach 40 ng/ml [76], and IL-18 can reach 50 ng/ml [77]. Thus, we used these data as a reference to optimize the cytokine doses used to treat mice, considering that local TL1A and IL-18 concentrations may exceed systemic levels. We further investigated the levels of TL1A and IL-18 in thymus lysates from mice acutely infected with either PVM or MCMV. Our results revealed elevated levels of both TL1A and IL-18 during PVM- and MCMV-induced acute thymus atrophy, suggesting that they might play a role in triggering acute thymus atrophy during viral infections, similar to what was observed following the in vivo administration of these two cytokines. Interestingly, the proportion of thymic neutrophils was increased in the atrophied thymuses of infected mice, although the absolute neutrophil counts were either unchanged or decreased in both viral models. Thus, it is tempting to speculate that during acute and systemic inflammation, thymus neutrophils might egress to the periphery as the *“marginated pool”* of neutrophils in peripheral organs that is mobilized into circulation following infection, as reported for other organs during emergency myelopoiesis [33, 35, 37].

As the NTOC system excludes the influx of bone marrow progenitors, we hypothesized that thymus neutrophils could originate from thymus precursors. Our scRNAseq, flow cytometry, and EM data revealed different stages of neutrophil differentiation, confirming our hypothesis that in situ development of thymus neutrophils occurs in the NTOC system. To further explore the ontogeny of thymic neutrophils and thymus progenitors, we employed state-of-the-art fate-mapping tools. Our data revealed that a significant portion of thymic neutrophils were *Rag1*-labeled, implying a shared origin with T cells in the thymus and indicating that the thymic neutrophil compartment may comprise both bone marrow-derived neutrophils (YFP^-^) and thymus-derived neutrophils (YFP^+^). To delve deeper into our findings, we opted to investigate *Rag1* labeling in both thymic-, and bone marrow progenitor subsets. Based on previous reports, we designed a gating strategy to distinguish different subsets of thymus progenitors [19, 55]. Our results confirmed the presence of GMPs in the murine thymus, as previously reported only for the human thymus [2, 78]. In this report, the use of the *Rag1*-Cre and *Ms4a3*-Cre fate mapping models suggested that a substantial fraction of thymic neutrophils arise from thymic *Rag1*^+^ GMP progenitors and develop in situ rather than from external sources [14]. Approximately 2% of bone marrow GMPs showed a history of *Rag1* expression, compared to ~67% of thymic GMPs. These proportions are remarkably similar to the proportions of neutrophils with a history of *Rag1* expression in the bone marrow (~2.5%) and thymus (~60%). Interestingly, the strikingly similar frequencies of neutrophils and GMPs with a history of *Rag1* expression in both the thymus and bone marrow suggest a direct association between GMPs and neutrophil labeling in these organs. Although the majority of thymic-derived neutrophils have a history of *Rag1* expression, the fact that a small fraction of bone marrow GMPs also show a history of *Rag1* expression makes the *Rag1* fate-mapping model unsuitable for tracking thymus-derived neutrophils in the periphery in vivo.

Collectively, our results verified the potential of TSPs to adopt a myeloid fate, as recently described [2, 78]. Our data could indicate that the thymic microenvironment induces *Rag1* expression for priming toward T-cell fate, but some cells escape this signaling pathway and develop into neutrophils. Alternatively, *Rag1* expression on bone marrow progenitors might reflect lymphoid priming prior to thymus colonization [79, 80]. Notably, VDJ rearrangements have been reported in thymic NK/ILCs [81] and DCs [2, 78], indicating that they are of thymic origin. Murine thymic granulocytes exhibit Dβ1-Jβ1 rearrangements, which is a hallmark of their thymic origin [16]. Moreover, a small fraction of human neutrophils express RAG1/2 recombinases and components of the TCRαβ complex [82]. Taken together, these reports support our findings and suggest that the neutrophil developmental pathway in the thymus is distinct from that in the bone marrow.

To determine the impact of the proinflammatory cytokines TL1A and IL-18 on thymic development and the mechanism driving reprogramming toward the production of thymic neutrophils, we performed scRNA-seq and flow cytometry. Our data revealed that thymic ILC1s, ILC2s, and γδT cells coexpressed *Tnfsrs25* (DR3), *Il18r1* (IL-18Rα) and *Csf2* (GM-CSF). We hypothesized that these cells were the first responders to TL1A + IL-18 treatment and produced GM-CSF, thereby promoting the expansion of thymic neutrophils. Unbiased NicheNet analysis further supported our hypothesis of a *Csf2*-*Csf2rb* ligand‒receptor interaction between ILC1s and thymic neutrophils. We confirmed this mechanism by performing intracellular flow cytometry, which revealed ILC1s as  one of the main sources of GM-CSF in the thymus. By using congenic *Csf2rb*^-/-^ mice and antibody blockade, we showed that thymic granulopoiesis can be blocked in a GM-CSF-dependent manner. Thus, we confirmed a direct link between TL1A and GM-CSF production in γδT cells and ILCs, in line with previous reports [49, 83].

Additionally, our NicheNet analysis indicated that treatment with TL1A and IL-18 might shift the ligand-pair interaction between ILCs/γδT cells and neutrophils, suggesting the downregulation of *Notch*. Through the inhibition of NOTCH downstream signaling using the γ-secretase inhibitor LY411575, our results showed that NOTCH plays a role in limiting the development of myeloid cells in the thymus. This finding is consistent with those of previous studies [10, 59, 60]. Treatment with TL1A and IL-18 could disrupt NOTCH signaling, leading to NOTCH downregulation, thereby opening a window for the development of non-lymphoid lineages. While we show that thymic neutrophil development is controlled by NOTCH and GM-CSF, it remains unclear whether the thymic atrophy process itself is required for thymic neutrophil development or whether these events are uncoupled. Alternatively, thymus granulopoiesis could be a symptom of more optimal conditions for thymus neutrophil development due to the impact of treatment on the thymic milieu. TL1A + IL-18 treatment reduced the expression levels of the key transcription factors involved in T-cell development, *Tcf7* and *Bcl11b* [81, 84], and increased the expression levels of *PU.1 and Cebpe*, which are known to be important for myeloid development [85]. The underlying genetic network that determines T-cell versus myeloid fate in the thymus is dominated by *Notch*, which controls the T-cell specification of non-committed progenitors by regulating *PU.1* and *Cepba* [13]. Interestingly, ETPs expressing the IL-4Ra/IL-13Ra1 receptor adopt a myeloid fate upon IL-13 binding via STAT6 signaling, resulting in *Notch1* downregulation and subsequent *Cebpa* upregulation, leading to myeloid development [86]. Relatedly, TL1A has been shown to induce IL-13 production by ILCs [87] and T cells [88]. Therefore, TL1A + IL-18 administration might promote thymic neutrophil development beyond GM-CSF production [89]. Moreover, in the NTOC setup, we observed increased levels of IL-22 in response to synergistic treatment with TL1A + IL-18. IL-22 production by ILCs in the thymus reportedly contributes to tissue repair after damage by stimulating the survival and proliferation of thymic epithelial cells [68]. Our findings, combined with the literature, suggest that thymic ILCs play a pivotal role as sentinels, instructing thymus progenitor fate decisions by providing cytokine signals, playing a role in thymus regeneration, and driving neutrophil production in response to TL1A + IL-18.

Thymic neutrophils have been shown to manifest immunomodulatory properties and distinct transcriptional features [78] compared to peripheral neutrophils [76]. Local neutrophil development may be associated with organ repair or other tissue-specific functions in the thymus [90, 91]. In fact, recent studies have challenged the classical notion of neutrophils as homogeneous, short-lived, and circulating immune cells, emphasizing that neutrophils can be tissue-resident and longer-lived and can adopt features tailored to the needs of specific tissues [92]. Due to the unknown nature of thymic neutrophils, we performed array functional assays to assess their functionalities compared to those of peritoneal neutrophils. In the TL1A + IL-18-treated group, NTOC-derived neutrophils produced high amounts of ROS, displayed high phagocytic capacity, and exhibited chemotactic responsiveness to CXCL1. Notably, some of the thymus-exiting neutrophils in the culture had an immature “band-like” nuclear morphology, primarily observed in blood circulation during severe inflammation [93]. Interestingly, thymic neutrophils express high levels of metalloproteases (*Mmp8* and *Mmp9*) [94, 95], which are involved in collagen degradation and matrix remodeling [92], possibly contributing to their egress into the supernatant. Therefore, thymic neutrophils might be implicated in thymic remodeling following damage; however, further research is needed to assess the local functions of thymic neutrophils. In the NTOC culture, alterations in the thymus extracellular matrix may lead to the release of progenitor cells into the supernatant, supporting our EM analysis showing early stages of neutrophil differentiation in the supernatant. These cells in the supernatant could potentially escape NOTCH signaling,  which directs thymic progenitors toward adopting a T-cell fate. This could explain the 23-fold increase in neutrophil counts between the NTOC supernatant and thymic lobes.

Ultimately, thymic atrophy can be therapeutically targeted to strengthen thymic output with beneficial effects on the health of the individual. Our data suggest that TL1A and IL-18 are possible targets for preventing thymic atrophy and restoring normal thymic function. As increased levels of TL1A have been associated with various pathologies, such as psoriasis [96], rheumatoid arthritis (RA) [71], asthma [84] and airway fibrosis [97], in which neutrophils can have deleterious effects, TL1A blockade could be a promising alternative to alleviate some of the negative impacts of neutrophils under these conditions.

## Methods

### Animals

All transgene mice and their littermate controls (when indicated) were on a C57BL/6J background. All wild-type mice except for the transgene littermate controls were C57BL/6N. Time-mated pregnant females and adult C57BL/6N mice were purchased from Janvier. Transgenic *Rag1*-Cre^tg/+^ mice were obtained from Yu Zang and Terry Rabbits [98] and crossed with Rosa26-flox-stop-flox-YFP mice (Gt(ROSA)26Sor^tm1(EYFP)Cos^) to generate *Rag1*-Cre Rosa26-YFP mice. *Ms4a3*-Cre mice (C57BL/6J-*Ms4a3*^*em2(cre)Fgnx*^/J) were generously provided by Florent Ghinoux [61] and crossed with ROSA26–flox-stop-flox-tdTomato mice (B6.Cg-*Gt(ROSA)26Sor*^*tm14(CAG-tdTomato)Hze*^/J) to create *Ms4a3*-Cre Rosa26Tdtomato. *Rag2*^−/−^OTI (B6.129S6-Rag2tm1Fwa Tg(TcraTcrb)1100Mjb) and *Csf2rb* knockout (B6.129S1-*Csf2rb*^*tm1Cgb*^/J) mice were kindly provided by Bart Lambrecht. TCRd knockout mice (B6.129P2-Tcrd^tm1Mom^/J) were purchased from Jackson. All transgenic mice were bred and kept in-house at the VIB Center for Inflammation Research under specific pathogen-free (SPF) conditions. All mouse experiments were conducted according to institutional, national, and European animal regulations. The Ethics Committee of Ghent University approved all the experimental animal procedures (EC2020-001, EC2022-110, and EC2023-100). Experiments were conducted in agreement with the European Parliament’s Directive 2010/63/EU and the 22-09-2010 Council on the protection of animals used for scientific purposes.

### Neonatal thymic organ culture (NTOC)

Neonatal thymuses from neonates (P0.5) were surgically removed and placed in 12-well plates with Isopore membrane filters (Merck Millipore, #ATTP01300) in complete medium: D-MEM (GIBCO, #31330-038) supplemented with 20% FCS (Greiner, #71133), 20 μM L-glutamine, 50 μM 2-mercaptoethanol, 0.8% penicillin-streptomycin (Sigma‒Aldrich, #P4333), 0.4 mM Na-pyruvate (Sigma‒Aldrich, #S-8636), and 1 × nonessential amino acids (Sigma‒Aldrich, #M7145). The plates were incubated at 37 °C and 5% CO_2_ for up to 6 days. Cytokines were used at the following concentrations: TL1A [100 ng/ml] (Biotechne - R & D Syst. Eur., #1896-TL-010), and IL-18 [40 ng/ml] (Prepotech, #B002-5).

### Transmission electron microscopy (TEM)

Thymic CD4^+^ T cells (TCRb^+^CD4^+^CD8b^-^), neutrophils (Ly-6G^+^CD4^-^CD8b^-^TCRβ^-^TCRγδ^-^CD19^-^MHCII^-^CD11c^-^) and monocytes/macrophages (MHCII^+^CD4^-^CD8b^-^TCRβ^-^TCRγδ^-^CD19^-^CD11c^-^) were sorted from the supernatant of the NTOC on Day 6 and treated with either vehicle or TL1A + IL-18. Bone marrow cells from neonatal mice were isolated by grinding the tibia and fibula with a mortar and pestle. The cells were then recovered in FACS buffer and filtered through a 70 μm filter. The resulting single-cell suspensions were subjected to neutrophil enrichment using the EasySep™ Mouse Neutrophil Enrichment Kit (StemCell Technologies, #19762). Thymic sorted cells and bone marrow-enriched neutrophils were fixed and processed as described in the Supplementary Information. Images were acquired from grids with a JEM 1400plus transmission electron microscope (JEOL, Tokyo, Japan) operating at 80 kV. The scale bar was set to 2 µm, and images were taken at 5000× magnification.

### Injection models

Adult (8 weeks old) or neonatal mice (P3) were randomly assigned to groups. All mice received four intraperitoneal (IP) injections of cytokines or vehicle at an interval of 24 h. P3 neonates were injected IP with cytokines diluted in PBS containing 0.1% BSA in a total volume of 20 µl using a 29 G needle. Cytokine doses: TL1A [250 ng/day]; IL-18 [100 ng/day]. Adults were IP injected with cytokines diluted in PBS containing 0.1% PBS in a total volume of 200 µl using a 29 G needle. Cytokine doses: TL1A [1000 ng/day]; IL-18 [750 ng/day]. Weight, temperature, and vital signs were monitored throughout the entire duration of the experiment for both neonates and adults.

### Tissue preparation for flow cytometry and cell sorting

Thymic lobes, NTOC thymic lobes, and spleens were processed into single-cell suspensions by pressing the organs through 70 μm cell strainers (VWR International, #BDAA352350), while the NTOC supernatant cells were harvested by pipetting off the media in the wells and subsequently collected into Eppendorf tubes. The spleens were further processed with ACK lysis buffer (Westburg b.v., #10-548E) for 3 min at room temperature (RT) to remove red blood cells. Blood was collected from adult mice (150 µl) via the saphenous vein, and blood samples were mixed with ACK lysis buffer and prepared for staining. Bone marrow cells were acquired by isolating the tibia and fibula of both legs. Adult tibias/fibulas were placed open in a microcentrifuge tube inside an Eppendorf tube and spun down at 1900 × *g* for 1 min at RT. Lungs were dissected and minced with scissors and transferred to 15 ml Falcon tubes containing 2 ml of digestion fluid consisting of RPMI, 200 µg/ml Liberase TM (Roche, #5401119001), and 10 ng/ml DNase I (Roche, #10104159001). Lung samples were incubated in a 37 °C water bath for 45 min, after which 5 ml of FACS-EDTA buffer was added, and the cells were transferred to new 15 ml Falcon tubes through 70 µm cell strainers. Finally, lysis buffer was added, and the samples were spun down at 400 × *g* at 4 °C for 5 min.

Subsequent flow cytometry or FACS analysis of all the samples was performed on ice. Antibody staining was performed for 30 min in the dark. For intracellular staining, the cells were fixed after extracellular staining using the eBioscience™ Foxp3/Transcription Factor Staining Buffer Set (Thermo Fisher, #00-5523-00). For intracellular cytokine measurements, cells were first extracted from each tissue as mentioned above and incubated with brefeldin A [1.5 µM] (Enzo Life Sciences, #BML-G405-0025) and monensin [2 µM] (BioLegend, #420701) for 4 h, followed by extracellular staining, fixation, and permeabilization using BD Cytofix/Cytoperm™ (BD Biosciences, #554714). Fixable Viability Dye eFluor™ 780 (Thermo Fisher, #65-0865-14) was used to exclude dead cells in all flow cytometry experiments. For counting purposes, we added 10 µl/sample of e123count eBeads (Thermo Fisher, #01-1234-42), or samples were counted in a BD FACSVerse™ Cell Analyzer. Single-cell suspensions were analyzed on a BD FACSymphony™ A5, BD FACSymphony™ A3 or BD LSRFortessa™ or purified using a BD Symphony S6, BD FACSAria II or III. All antibodies used for flow cytometry and cell sorting and commercial kits are shown in Supplementary Tables 1 and 2.

For flow cytometric analysis of thymic and bone marrow progenitors, thymuses from 8-weeks-old *Rag1*-Cre^+/tg^ Rosa26YFP^+/tg^ mice were isolated as described above and subsequently subjected to negative bead depletion with CD4-biotin (1:50) (thymuses) or CD19-biotin (1:100), CD11b-biotin (1:100), Ter-119-biotin (1:200) or CD3-biotin (1:200) (bone marrow) in a total volume of 0.5 ml. After 20 min of incubation at RT, 100 µl/10^7^ cells of MagniSort™ Streptavidin Negative Selection Beads (eBioscience, #MSNB-6002) were added to each tube. Single-cell suspensions were transferred to 5 ml polystyrene round-bottom tubes with a cell-strainer cap (VWR International, #734-0001) and incubated for 15 min at RT. The tubes were placed in a MagniSort™ Magnet (eBioscience, #MAG-4902-10) and incubated for 5 min for magnetic separation. Purified samples were recovered in clean tubes and spun down at 500 × *g* at 4 °C for 5 min for antibody staining.

### CITE-seq of the NTOC supernatant day 6

*CITE-seq* (Fig. 1F, G, H, 3A–D and Supplementary Fig. 3a, b): All viable cells (DAPI^-^) from *Day 6 NTOC supernatant* (Fig. 1) treated with (1) vehicle, (2) TL1A, (3) IL-18, or (4) TL1A + IL-18 were FACS-purified (Aria III, BD Biosciences) and pelleted by centrifugation at 400 × *g* for 5 min at 4 °C. Prior to the CITE-seq experiment, the cells were incubated with a CD16/CD32 mAb (BD Bioscience, #553142) for 20 min at 4 °C to avoid non-specific Fc receptor binding of CITE-seq antibodies. The cells were washed in excess PBS supplemented with 2% FCS (International Medicine, TICO, #8580166, Lot number: 90439) and used directly for downstream CITE-seq analysis. The NTOC supernatant of live cells (DAPI^-^) was purified by FACS-sorting with a BD FACSAria III. Following sorting, the NTOC supernatant cells and thymic lobe cells were stained with mouse cell surface protein TotalSeq-A antibody panels containing 9 isotype controls and 77 (NTOC) or 174 (tissue) oligo-conjugated antibodies (TotalSeq-A, BioLegend) (see Supplementary Table 3). The sorted single-cell suspensions were resuspended at final estimated concentrations of 1500 and 1100 cells/µl for the NTOC and tissues, respectively. In accordance with the manufacturer’s instructions for the 10x Genomics platform, 15,000 cells per group were FACS-purified and sent for sequencing.

### scRNAseq of the neonatal thymus and NTOC lobes at Days 1.5 & 3

Thymic lobes were enzymatically digested for 30–40 min in a 37 °C water bath with the following digestion mix: RPMI (Thermo Fisher, #11875093), 2% FCS, 2 mM EDTA (Invitrogen, #15575020), 0.125 mg/ml Liberase^™^ (Roche, #5401119001), 1 mg/ml Dispase II (Roche, #04942078001) and 10 ng/ml DNase I (Roche, #10104159001). After digestion, the remaining pieces were filtered through a 70 μm strainer and washed with 5–10 ml of MACS buffer (1 × PBS with 5 mM EDTA and 2% FCS). The cells were centrifuged at 400 × g and 4 °C for 5 min. Non-lymphoid cell subsets were enriched by using biotinylated antibodies as follows: (1) biotin-conjugated anti-CD4 [1:100], (2) biotin-conjugated anti-CD8α [1:100], (3) biotin-conjugated anti-CD19 [1:200] and (4) biotin-conjugated anti-Ter-119 [1:200]. After a 15 min incubation, the cells were centrifuged at 400 × *g* and 4 °C for 5 min to remove excess nonbound antibodies, at which point 150 µl/sample of MagniSort™ Streptavidin Negative Selection Beads (eBioscience, #MSNB-6002) was added, and the mixture was incubated for 10 min at RT. The samples were placed in a MagniSort™ Magnet (eBioscience, #MAG-4902-10) for 5 min at RT, and the purified enriched cells were recovered in clean round bottom polystyrene tubes ready for sorting. In accordance with the manufacturer’s instructions for the 10x Genomics platform, 20,000 cells per group were isolated and sent for sequencing. Additional information about the antibodies (Supplementary Table 1) and downstream analysis can be found in the Supplementary Information.

### Multiplex ELISA

A customizable multiplex U-PLEX ELISA (MSD, Rockville, MD) was used to measure the concentrations of cytokines in the NTOC culture supernatant. The following murine cytokines were measured: *GM-CSF, IL-17A, IL-5, IFNγ, IL-10, TNFα, IL-22*, and *IL-6*. NTOC supernatants were centrifuged at 10,000 × g to remove cells and cellular debris from the samples before assay measurements.

### NET formation assay and imaging

Adult C57BL/6N mice (8 weeks old) were IP injected with 1 ml of 3% Brewer thioglycolate (Sigma‒Aldrich, #B-2551) for 4 h prior to the isolation of peritoneum exudate cavity cells (PECs). The cells were recovered in 15 ml Falcon tubes in PBS, 2% FCS, and 1 mM EDTA buffer. NTOC neutrophils were isolated from the supernatants of thymic lobes treated with TL1A + IL-18 for 6 days. Both peritoneal cells and thymocytes were subjected to further enrichment with the EasySep™ Mouse Neutrophil Enrichment Kit (STEMCELL Technologies, #19762). Isolated peritoneal and thymic neutrophils were treated with PBS, DMSO (Sigma‒Aldrich, #D-2650), LPS [4 µg/ml] from *Klebsiella pneumoniae* (Sigma‒Aldrich, #L-4268) or ionomycin [2.5 µg/ml] (VWR International, #CALB407952). The percentage of NETotic events was calculated based on the area of dying cells (size exclusion: <30 µm^3^ from SYTOX Green and <25 µm^3^ from DAPI). Neutrophils isolated from the thymus and peritoneum were treated with DMSO (control) or ionomycin as indicated above and stained with DAPI (Sigma‒Aldrich, #D9542), SYTOX Green (Life Technologies Europe B.V., #S-7020), primary antibodies against elastase (Santa Cruz, #SC-9521, 1:500) and citrullinated histone H3 (Abcam, #ab5103, 1:500) and secondary antibodies against goat-anti-rabbit DyLight633 (Thermo Fisher, #35563, 1:2000) and donkey-anti-goat Alexa Fluor 568 (Thermo Fisher, #A11057, 1:2000), as previously described [99].

### Reactive oxygen species (ROS) production assay

NTOC supernatant cells were recovered as described previously. Peritoneal cells were recovered 4 h after thioglycolate injection as described above and further enriched with the EasySep™ Mouse Neutrophil Enrichment Kit (StemCell Technologies, #19762). Both NTOC-, and PEC-derived cells were subjected to extracellular flow cytometry as described previously. After staining, the cells were incubated in medium (RPMI, 20% FCS) for 30 min with 5 µM DHR 123 (Cayman Chemical, #85100) and either with or without eBioscience™ Cell Stimulation Cocktail (500X) (Thermo Fisher, #00-4970-93).

### Phagocytosis assay

On Day 6, NTOC supernatants from cells treated with TL1A + IL-18 and peritoneal neutrophils were obtained as described above. NTOC-derived neutrophils and peritoneal neutrophils were seeded in a 96-well plate at 100,000 cells/well and incubated with pHrodo™ Red *S. aureus* BioParticles [1 mg/ml] (Thermo Fisher, #A10010) supplemented with 2 µM cytochalasin D (Sigma, C8273) at 37 °C and 5% CO_2_ for 60 min. Phagocytosis was monitored with an IncuCyte S3 live cell imaging device (Essen Bioscience, Germany). Phagocytic events were normalized by cell counts.

### Migration assay

Neutrophils were isolated from Day 6 NTOC supernatant cells treated with TL1A + IL-18, and the peritoneal cavity was seeded at 150,000 cells/well in a 12-well Transwell plate of 6.5 mm with 5.0 µm pores in a polycarbonate membrane insert (Corning, #CLS3421-48EA) containing 600 µL of HBSS-2% FCS and incubated with either vehicle (PBS), 50 ng/mL CXCL1 (BioLegend, #573704), or 10 µM N-formyl-methionyl-leucyl-phenylalanine (fMLP) at 37 °C and 5% CO_2_ for 1.5 h. Migration events were counted in a BD FACSVerse™ Cell Analyzer.

### Murine cytomegalovirus (MCMV) and pneumonia virus (PVM) infection in mice

Eight-week-old wild-type females (C57BL/6 N) were IP injected with a 1:30 dose of MCMV or infected with 17.5 pore-forming units (PFUs) of PVM by instillation (IT) in a final volume of 200 µL. Weight and vital signs were monitored throughout the duration of the experiment. MCMV-infected mice and controls were sacrificed at 2 (early infection), 5 (acute infection), and 8 (recovery) days postinfection (dpi). PVM-infected mice and controls were sacrificed at 5 (incubation phase), 10 (acute infection), and 14 (recovery) dpi. The thymus and blood were collected for downstream flow cytometric and ELISA analyses.

### ELISAs for TL1A and IL-18 in thymus lysates

The left thymic lobes of the PVM, MCMV and control mice were collected and stored at −20 °C. Thymus lysates were prepared by TissueLyser II (Qiagen) with RIPA lysis buffer. The homogenates were centrifuged at 12,000 × *g* at 4 °C for 10 min. The supernatants were collected for further analysis. The protein concentration was determined with a BCA protein assay kit (Thermo Fisher, #23225). TL1A (R&D Systems, #DY1896-05) and IL-18 (Thermo Fisher #88-50618-88) ELISAs were performed according to the manufacturer’s instructions.

### Data analyses and statistics

In all mouse experiments, the mice were randomly assigned to groups. No statistical methods were used to predetermine sample sizes. Differences in variances between treatment groups were tested by the F test. When the variances were different, statistics were performed on log2-transformed values or a nonparametric test was used as indicated. Statistical analyses were carried out using GraphPad Prism version 9.5.1 (GraphPad Software Inc., La Jolla, CA).

Expanded methods and additional information about the mice, genotyping, and antibodies and reagents used can be found in the Supplementary Information.

## Supplementary information

Supplementary information

Source data from Figure 1

Source data from Figure 2

Source data from Figure 3

Source data from Figure 4

Source data from Figure 6

Source data from Figure 7

Supplementary Figure 1

Supplementary Figure 2

Supplementary Figure 3

Supplementary Figure 4

Supplementary Figure 5

Supplementary Figure 6

Supplementary Figure 7

Supplementary Figure 8

Supplementary Figure 9

Supplementary Figure 10

Supplementary Figure 11

Supplementary Figure 12

Supplementary Figure 13

## Data Availability

The single-cell sequencing data are deposited in the Gene Expression Omnibus as series *GSE244172*. Two CITE-seq samples (Vehicle and IL-18) had been uploaded previously as *GSM7169532* and *GSM7169534* of series GSE229632. All data needed to obtain the conclusions in this work are present in the manuscript or in the Supplementary Figures and Supplementary Tables. All mouse lines, reagents, and software used are listed in the manuscript or in the Supplementary Methods.
