# Supplementary material for: TL1A and IL-18 synergy promotes GM-CSF-dependent thymic granulopoiesis in mice
Source: Cell Mol Immunol. 2024 Jun 5;21(8):807–25. doi: 10.1038/s41423-024-01180-8 (PMC11291760; doi:10.1038/s41423-024-01180-8)
Supplement: Supplementary file 1 — Supplementary information [file 41423_2024_1180_MOESM1_ESM.docx]

**Supplemental Methods**

***Genotyping***

***Rag1*-Cre Rosa26-YFP** mice were genotyped using the following PCR primers: *Rag1*-Cre Forward primer *GCCTGCATTACCGGTCGATGCAACGA*, Reverse primer *GTGGCAGATGGCGCGGCAACACCATT*, Rosa26-YFP Forward-1 primer *AAAGTCGCTCTGAGTTGTTAT*, Forward-2 *GCGAAGAGTTTGTCCTCAACC* and Reverse *GGAGCGGGAGAAATGGATATG* Yielding a 160 bp wild-type DNA band at 160 bp and a transgene DNA band of 245bp. ***Csf2rb*-KO** mice were genotyped using the PCR primers: Forward-1 *ATATTGCTGAAGAGCTTGGCGGC,* Forward-2 *GTGTAGACACTGGCCCCCG* and Reverse *GAACCTTCAATGCTTCTTTGATGGGAT,* yielding a 300 bp wild-type DNA fragment and a 648 bp knockout DNA fragment. ***Ms4a3*-Cre Rosa26Tdtomato** mice were genotyped using the following PCR primers: *Ms4a3*-Cre Forward-1 primer *GATGGTCTCTGGTCCACATGC, forward-2* ACCCTGTTACGTATAGCCGA, Reverse-1 primer *CAGACCGTGCTCGTGGATTT*, wild-type DNA band at 396 bp and a transgene DNA band of 664 bp. Rosa26Tdtomato wild-type forward primer *CAGCTTCTTGTAATCGGGGA*, wild-type reverse primer *GCGAGGAGGTCATCAAAGAG* Forward-1 primer *AAAGTCGCTCTGAGTTGTTAT*. ***Rag2*^-/-^ OT-I mice** were genotyped using the PCR primers: Rag2^−/−^, CAGGGTCGCTCGGTGTTC (forward-1), CTTGCCAGGAGGAATCTCTG (forward-2), and GTTTCCCATGTTGCTTCCA (reverse), yielding a 200-bp wild-type DNA fragment and a 388-bp knockout DNA fragment. OTI: OTI TCR, CAGCAGCAGGTGAGACAAAGT (forward) and GGCTTTATAATTAGCTTGGTCC (reverse); OT Int. Ctrl, CAAATGTTGCTTGTCTGGTG (forward) and GTCAGTCGAGTGCACAGTTT (reverse), yielding a 200-bp wild-type DNA band and a 300-bp transgene DNA band. ***TCRd*-KO** mice were genotyped using the PCR primers: Mutant Forward *CTTGGGTGGAGAGGCTATTC,* Mutant Reverse *AGGTGAGATGACAGGAGATC,* Internal Positive Control Forward *CAAATGTTGCTTGTCTGGTG* and Internal Positive Control Reverse *GTCAGTCGAGTGCACAGTTT,* yielding a 206 bp wild-type DNA fragment and a 280 bp knockout DNA fragment.

***Transmission electron microscopy (TEM)***

Thymic CD4^+^ T cells (TCRb^+^CD4^+^CD8b^-^), Neutrophils (CD11b^+^Ly-6G^+^CD4^-^CD8b^-^TCRβ^-^TCRγδ^-^CD19^-^MHCII^-^CD11c^-^) and Monocytes/Macrophages (MHCII^+^CD4^-^CD8b^-^TCRβ^-^TCRγδ^-^CD19^-^ CD11c^-^) were sorted from NTOC supernatant Day 6 treated with vehicle and TL1A+IL-18 respectively. Sorted thymus cells from the NTOC, thymus lobes, and isolated bone marrow neutrophils were fixed in 4% PFA and 2.5% glutaraldehyde in 0.1 M NaCacodylate buffer, pH 7.2 and spun down at 1500 rpm. Low melting point agarose (1%) was used to keep the cells concentrated for further processing. The solidified agarose was cut in small pieces. Cells were washed three times in NaCacodylate buffer. After washing in buffer, they were post-fixed in 1% OsO4 with 1.5% K3Fe(CN)6 in 0.1 M NaCacodylate buffer at room temperature for 1 hour. After washing, cells were subsequently dehydrated through a graded ethanol series, including a bulk staining with 1% uranyl acetate at the 50% ethanol step followed by embedding in Spurr’s resin. Ultrathin sections of a gold interference color were cut using an ultramicrotome (Leica EM UC6), followed by a post-staining in a Leica EM AC20 for 40 min in uranyl acetate at 20°C, and thereafter 10 min in lead stain at 20°C. Sections were collected on Formvar-coated copper slot grids. Grids were viewed with a JEM 1400plus transmission electron microscope (JEOL, Tokyo, Japan) operating at 80 kV. Scale bar was set at 2 µM and all images were taken at 5000X magnification.

***PVM batch preparation***

To freshly prepare a viral batch, 20 BALB/c female mice of 7-weeks old were infected by instillation with a sublethal dose of PVM. Mice were sacrificed five days post infection and lungs were dissected. Lungs were homogenized in 2 ml Eppendorf tubes containing 1 ml HBSS, 20% of sucrose, 1% penicillin/streptomycin and homogenizing beads. Shortly after, lungs were homogenized in the tissue homogenizer by sonication. The samples were spun down for 10 min, at 3000 rpm and 4°C. After centrifugation, the interphase layer containing the virus was transferred to clean Eppendorf tubes and divided in 20 μl per tube and stored at -80°C.

***MCMV batch Preparation***

MCMV Smith strain was serially passaged in BALB/c animals once more upon arrival of the P2 virus batch provided by Sophie Janssens lab. Animal’s salivary glands were collected two weeks later and viral stocks were prepared by Dounce homogenation in DMEM (Gibco) supplemented with 3% FCS (Bodinco). Aliquots were stored at minus 80C.

***Cellular Indexing of Transcriptomes and Epitopes by Sequencing (CITE-seq) on NTOC supernatant day 6 and Neonate P0.5 Thymus***

**CITE-seq (Figures 1G, F, H, 3A-D and Supplemental figures 1F an G):** All viable cells (DAPI^-^) from **Day 6 NTOC** **supernatant** (**Figure 1**) treated with: (1) Vehicle, (2) TL1A, (3) IL-18, and (4) TL1A+IL-18 were FACS-purified (Aria III, BD Biosciences) and pelleted by centrifugation at 400 g for 5 min at 4°C. Prior CITE-seq performance, cells were incubated with CD16/CD32 mAb (BD Bioscience, #553142) for 20 min at 4°C to avoid non-specific Fc receptor binding of CITE-seq antibodies. Cells were washed in excess PBS with 2% FCS (International medical, TICO, #8580166, Lot number: 90439) and used directly in downstream CITE-seq analysis. Following sorting, both the NTOC supernatant cells and *ex vivo* tissue comparison cells were stained with mouse cell surface protein TotalSeq-A antibodies panels containing 9 isotype controls and 77 (NTOC) or 174 (tissue) oligo-conjugated antibodies (TotalSeq-A, Biolegend) (see **Supplementary Table 3**). The sorted single-cell suspensions were resuspended at a final estimated conc. of 1500 and 1100 cells/µl for NTOC and tissues respectively. The cells were loaded on a Chromium GemCode Single Cell Instrument (10x Genomics) to create single-cell gel beads-in-emulsion (GEM). The scRNA-Seq libraries were prepared using GemCode Single Cell 3’ Gel Bead and Library kit, version 3 (NTOC supernatant) and version NextGEM 3.1 (Thymus lobes & NTOC lobes) (10x Genomics) according to the manufacturer’s instructions with the addition of amplification primer (3 nM, *5’CCTTGGCACCCGAGAATT*C*C*) during cDNA amplification to enrich the TotalSeq-A cell surface protein oligos. Size selection with SPRIselect Reagent Kit (Beckman Coulter, #B23318) was applied to separate the amplified cDNA molecules for 3’ gene expression and cell surface protein construction. TotalSeq-A protein library construction including sample index PCR using Illumina’s Truseq Small RNA primer sets and SPRIselect size selection was performed according to the manufacturer’s instructions. The cDNA content of pre-fragmentation and post-sample index PCR samples was Analysed using 2100 BioAnalyzer (Agilent). Sequencing libraries were loaded on a HiSeq4000 flow cell (NTOC) and an Illumina NovaSeq flow cell (tissue) at VIB Nucleomics core with sequencing settings according to the recommendations of 10x Genomics, pooled in a 90:10 ratio for the combined 3´ gene expression and cell surface protein samples respectively. The Cell Ranger pipeline (10x Genomics, version 3.1.0) was used to perform sample demultiplexing and to generate FASTQ files for read 1, read 2 and the i7 sample index for the gene expression and cell surface protein libraries. Read 2 of the gene expression libraries was mapped to the mouse reference genome (GRCm38.99). Subsequent barcode processing, unique molecular identifiers filtering and gene counting was performed using the Cell Ranger suite. CITE-seq reads were quantified using the feature-barcoding functionality.

***Single-cell RNA seq on Neonate thymus steady state & NTOC lobes days 1.5 & 3***

**scRNAseq** (**Figures 6b-c, Supplementary figures 9b, 9c and 9d**): Samples were split up in **3 groups:** (1) *non-preprocessed samples*, (2) *T cell-depleted samples* and (3) *Thymus epithelial cells (TECs)-enriched sample*. For each condition/treatment 20 neonatal thymuses were pooled together. We had the following conditions: (1) Neonatal thymuses (P0.5), (2) NTOC vehicle - day 1.5, (3) NTOC TL1A+IL-18 - day 1.5, (4) NTOC vehicle - day 3, and (5) NTOC TL1A+IL-18 - day 3. For the non-processed sample, single-cell suspensions were obtained as described previously. T cell-depleted sample was obtained by negative beads enrichment using biotinylated antibodies as follows: (1) Biotin-anti-CD4 [1:100], (2) Biotin-anti-CD8α [1:100], (3) Biotin-anti-CD19 [1:200] and (4) Biotin-anti-Ter-119 [1:200]. Additional information about the antibodies is shown in **Supplemental Table 1**. Samples were incubated for 15 min at RT. After 15 min incubation, the cells were centrifuged at 400 g, 4ºC for 5 min to remove the excess of non-bound antibodies. 150 µl of MagniSort™ Streptavidin Negative Selection Beads (eBioscience, #MSNB-6002) were added per sample and incubated for 10 min at RT. The samples were placed in a MagniSort™ Magnet (eBioscience, #MAG-4902-10) for 5 min at RT and purified-enriched cells were recovered in clean round bottom polystyrene tubes ready for sorting. Thymus epithelial cells (TECs)-enriched samples were obtained by enzymatic digestion with the following digestion mix: RPMI (Thermofischer, #11875093), 2% FCS, 2 mM EDTA (Invitrogen, #15575020), 0.125 mg/ml Liberase^™^ TM (Roche, #5401119001), 1 mg/ml Dispase II (Roche, #04942078001) and 10 ng/ml DNase I (Roche, #10104159001). Thymic lobes were incubated for 30–40 min in a 37°C water bath in the enzymatic solution. After digestion, the remaining pieces were filtered through a 70 μm mesh filter and washed with 5–10 ml MACS buffer (1× PBS with 5 mM EDTA and 2% FCS). Cells were centrifuged at 400 g, 4ºC for 5 min. Lastly, cells were subjected to negative bead enrichment with the following biotinylated antibodies: (1) Biotin-anti-CD4 [1:100], (2) Biotin-anti-CD8β [1:100], (3) Biotin-anti-CD19 [1:200], (4) Biotin-anti-CD11b [1:300], and (4) Biotin-anti-Ter-119 [1:200]. Additional information about the antibodies is summarized in **Supplementary table 1**. For FACS and hashtag labelling, progenitors and bulk populations were counted, isolated and spun down. The cell pellet was resuspended and incubated for 30 min on ice with staining mix in PBS containing 0.04% BSA, FACS antibodies (detailed in **Supplementary table 1**) , TruStain FcX Block (BioLegend, #101320) and a unique mouse TotalSeq-C cell hashing antibody (Biolegend) diluted 1:1200. Bulk population and progenitors were multiplexed per lane using TotalSeq-C cell hashing antibodies. FACS-sorted cells were pooled in a **(1)20:(2)60:(3)20 ratio**. Sorted single-cell suspensions were centrifuged and resuspended at an estimated final concentration of 500 cells/µl and loaded on a Chromium GemCode Single Cell Instrument (10x Genomics) to generate single-cell GEM. The scRNA-Seq libraries were prepared using the GemCode Single Cell 5’ Gel Bead and Library kit, version 2 (10x Genomics) according to the manufacturer’s instructions. Size selection with SPRIselect Reagent Kit (Beckman Coulter, #B23318) was used to separate amplified cDNA molecules for 5’ gene expression and cell surface protein construction. TotalSeq-C protein library construction including sample index PCR using Illumina’s Truseq Small RNA primer sets and SPRIselect size selection was performed according to the manufacturer’s instructions. The cDNA content of pre-fragmentation and post-sample index PCR samples was analysed using the 2100 BioAnalyzer (Agilent). Sequencing libraries were loaded on an Illumina NovaSeq flow cell at VIB Nucleomics core with sequencing settings according to the recommendations of 10x Genomics, pooled in a 90:10 ratio for the combined 5’ gene expression and hashing protein samples, respectively. The Cell Ranger pipeline (10x Genomics, version 6.0.0) was used to perform sample demultiplexing and to generate FASTQ files for read 1, read 2 and the i7 sample index for the gene expression and cell surface protein libraries. Read 2 of the gene expression libraries was mapped to the mouse reference genome (GRCm38.99). Subsequent barcode processing, unique molecular identifiers filtering and gene counting was performed using the Cell Ranger suite.

***scRNAseq analyses***

Both CITE-seq samples and scRNAseq samples were analysed with Seurat^1^ (v4.3.0.1), including the creation of UMAPs, violin plots, dotplots, featureplots, determination of marker genes and cluster annotation. Heatmaps were made with pheatmap (v1.0.12).

CITE-seq samples were merged into one aggregate. Slingshot^2^ (v2.8.0) was used for trajectory interference on the neutrophil clusters with pNeutrophils set as the start cluster. Slingshot information was used as input for TradeSeq^3^ (v1.14.0) to model gene expression in function of pseudotime across the trajectory. The gene ontology analysis on the neutrophils was performed with gprofiler2^4^ (v0.2.2).

For creating **Supplemental Figure 7d**, we analysed ATAC-seq from **Ferreira *et al.* (2021)**^5^. Bedfiles containing ATAC-seq reads were downloaded from the GEO website (GSE146745), specifically from the ETP samples (GSM4405359, GSM4685291  and GSM4685292). Reads were normalized for library size for visualization which was done using the *Gviz* R package (v 1.44.2)^6^. The TSS location was inferred from Ensembl (mouse genome version GRCm38.p6).

scRNAseq samples of Neonate thymus steady state & NTOC lobes days 1.5 & 3 were merged with neonate thymus sample (Sample [*GSM7169541*](https://www.ncbi.nlm.nih.gov/geo/query/acc.cgi?acc=GSM7169541) of the [*GSE229632*](https://www.ncbi.nlm.nih.gov/geo/query/acc.cgi?acc=GSE229632) series on Gene expression Omnibus), then ran through the default seurat processing pipeline using default parameters except for ScaleData function where all features were used for scaling rather than just the variable features, and the RunPCA function where npcs was set to 180. Integration was performed with Harmony^7^ (v0.1.1) using batch correction. Nichenet^8^ (v2.0.0) analysis was done by executing the differential NicheNet pipeline, using the default NicheNet resources for the ligand-receptor network and ligand-target matrix. “ILC1s” and “ILC2s” clusters were grouped as ILCs. “Developing γδT cells”, “γδT cells”, “Cytotoxic γδ T cells” and “γδT-17 cells” clusters were grouped as γδT cells. NicheNet niches were created per timepoint and per treatment. This means that in each niche, either ILCs or γδT cells of a particular timepoint and treatment were set as “sender cells” and paired with neutrophils of the same timepoint and treatment set as “receiver cells”.

**Supplemental Tables**

**Supplemental Table 1**: Flow cytometry, cell sorting and imaging antibodies

| Antigen | Fluorochrome | Clone | Source | Cat# | Dilution (1:X) | Notes |
| --- | --- | --- | --- | --- | --- | --- |
| Anti-Histone 3 (citrulline R2+R8+R17) |  |  | Abcam | ab5103 | 1:500 | Confocal images |
| Anti-Neutrophil Elastase |  |  | Santa Cruz Biotechnology | SC-9521 | 1:500 | Confocal images |
| CD101 | PE | Moushi101 | Thermofisher | 12-1011-82 | 1:100 |  |
| CD115 (CSF-1R) | BV650 | AFS98 | BD Biosciences | 750891 | 1:200 |  |
| CD115 (CSF-1R) | PE | AFS98 | Thermofisher | 12-1152-82 | 1:200 |  |
| CD115 (CSF-1R) | PE-Cy7 | AFS98 | Biolegend | 135524 | 1:200 |  |
| CD115 (CSF-1R) | Unconjugated | QA20A50 | Biolegend | 100002 | 1:100 |  |
| CD117 (c-Kit) | BB515 | 2B8 | BD Biosciences | 564481 | 1:100 |  |
| CD117 (c-Kit) | FITC | 2B8 | BD Biosciences | 564481 | 1:100 |  |
| CD117 (c-Kit) | APC | 2B8 | Biolegend | 17-1171-83 | 1:100 |  |
| CD117 (c-Kit) | PE | 2B8 | Biolegend | 105807 | 1:100 |  |
| CD11b | FITC | M1/70 | Thermofisher | 11-0112-82 | 1:400 |  |
| CD11b | BV421 | M1/70 | Biolegend | 101251 | 1:400 |  |
| CD11b | BV510 | M1/70 | BD Horizon | 562950 | 1:400 |  |
| CD11b | BV605 | M1/70 | BD Horizon | 563015 | 1:400 |  |
| CD11b | BV785 | M1/70 | Biolegend | 101243 | 1:400 |  |
| CD11b | AF647 | M1/70 | Biolegend | 101218 | 1:400 |  |
| CD11b | PE-Cy5 | M1/70 | Thermofisher | 15-0112-82 | 1:400 |  |
| CD11b - Biotin |  | M1/70 | Biolegend | 101203 | 1:200 | Negative beads enrichment |
| CD11c | AF488 | N418 | Biolegend | 117311 | 1:400 |  |
| CD11c | BV650 | N418 | Biolegend | 117339 | 1:400 |  |
| CD11c | BV785 | N418 | Biolegend | 117336 | 1:400 |  |
| CD11c | PE-eFluor610 | N418 | Thermofisher | 61-0114-82 | 1:400 |  |
| CD11c | PE-Cy5 | N418 | Biolegend | 117316 | 1:400 |  |
| CD11c | PE-Cy7 | N418 | Affymetrix | 25-0114 | 1:400 |  |
| CD122 | PerCP-eFluor710 | TM-β1 | Thermofisher | 46-1222-82 | 1:100 |  |
| CD122 | BV421 | TM-β1 | BD Horizon | 562960 | 1:200 |  |
| CD122 | PE | TM-β1 | Biolegend | 123210 | 1:200 |  |
| CD122 - Biotin |  | TM-β1 | Thermofisher | 13-1222-82 | 1:100 | Negative beads enrichment |
| CD123 (IL-3Ra) | Purified | 5B11 | Biolegend | 106002 |  |  |
| CD123 (IL-3Ra) | PE | 5B11 | Biolegend | 106005 | 1:100 |  |
| CD125 (IL-5Ra) | Purified | DIH37 | Biolegend | 153402 |  |  |
| CD125 (IL-5Ra) | PE-Cy7 | DIH37 | Biolegend | 153407 | 1:100 |  |
| CD127 (IL-7Rα) | BV605 | A7R34 | Biolegend | 135041 | 1:100 |  |
| CD127 (IL-7Rα) | BUV737 | SB/199 | BD Horizon | 564399 | 1:100 |  |
| CD131 (CSF2RB) | PE | JORO50 | BD Pharmigen | 559920 | 1:100 |  |
| CD135 (Flt3) | BV421 | A2F10 | Biolegend | 135315 | 1:100 |  |
| CD135 (Flt3) | PE-Cy7 | A2F10.1 | BD Pharmigen | 567594 | 1:100 |  |
| CD150 | PE | TC15-12F12.2 | Biolegend | 115904 | 1:100 |  |
| CD150 | PE-Cy7 | TC15-12F12.2 | Biolegend | 115914 | 1:100 |  |
| CD16/32 | BV711 | 93 | Biolegend | 101337 | 1:50 |  |
| CD184 (CXCR4) | BV711 | L276F12 | Biolegend | 146517 | 1:100 |  |
| CD19 | BV785 | B4 | Biolegend | 115543 | 1:200 |  |
| CD19 | PE-Cy5 | 1D3 | Thermofisher | 15-0193-82 | 1:200 |  |
| CD19 | BUV805 | 1D3 | BD Horizon | 56287 | 1:200 |  |
| CD19 - Biotin |  | 1D3 | Thermofisher | 13-0193-82 | 1:200 | Negative beads enrichment |
| CD197 (CCR7) | BV650 | 4B12 | BD Horizon | 564356 | 1:100 |  |
| CD199 (CCR9) | PE | CW-1.2 | BD Horizon | 565576 | 1:100 |  |
| CD24 | BUV737 | M1/69 | BD Horizon | 565308 | 1:200 |  |
| CD25 | PerCP-Cy5.5 | PC61 | BD Pharmigen | 551071 | 1:200 |  |
| CD25 | BV510 | PC61 | Biolegend | 102042 | 1:200 |  |
| CD25 | BV605 | PC61 | BD Horizon | 563061 | 1:200 |  |
| CD25 | PE | PC61 | BD Pharmigen | 553866 | 1:200 |  |
| CD25 | BUV395 | PC61 | BD Horizon | 564022 | 1:200 |  |
| CD27 | PE-Cy7 | LG.7F9 | Thermofisher | 25-0271-82 | 1:200 |  |
| CD27 | BUV737 | LG.3A10 | BD Horizon | 565307 | 1:200 |  |
| CD274 (PD-L1) | BV605 | MIH6 | Biolegend | 153606 | 1:100 |  |
| CD274 (PD-L1) | Purified | MIH5 | Thermofisher | 16-5982-81 | 1:100 |  |
| CD28 |  | 37.51 | BD Pharmigen | 553294 | 1:500 |  |
| CD28 | PE-Cy7 | E18 | Biolegend | 122014 | 1:200 |  |
| CD335 (NKp46) | BUV737 | 29A1.4 | BD Horizon | 565085 | 1:200 |  |
| CD335 (NKp46) | PE | 29A1.4 | Biolegend | 137603 | 1:200 |  |
| CD34 | eFluor450 | RAM34 | Thermofisher | 56-0341-82 | 1:100 |  |
| CD34 | AF647 | SA376A4 | Biolegend | 152205 | 1:100 |  |
| CD34 | AF700 | RAM34 | Thermofisher | 56-0341-82 | 1:100 |  |
| CD36 | PE | CRF D-2712 | BD Pharmigen | 562702 | 1:100 |  |
| CD36 | PE | CRF D-2712 | BD Pharmigen | 562702 | 1:100 |  |
| CD3ε | AF488 | 145-2C11 | BD Pharmigen | 557666 | 1:50 |  |
| CD3ε | BV711 | 17A2 | Biolegend | 100241 | 1:50 |  |
| CD3ε | AF647 | 17A2 | Biolegend | 100209 | 1:50 |  |
| CD3ε | PE | 145-2C11 | Affymetrix | 12-0031 | 1:50 |  |
| CD3ε | BUV395 | 145-2C11 | BD Horizon | 563565 | 1:50 |  |
| CD3ε | BUV563 | 500A2 | BD Horizon | 741235 | 1:50 |  |
| CD3ε |  | 145-2C11 | Biolegend | 100302 |  |  |
| CD4 | eFluor450 | RM4-5 | Affymetrix | 48-0042 | 1:400 |  |
| CD4 | BV510 | RM4-5 | Biolegend | 100559 | 1:400 |  |
| CD4 | BV605 | RM4-5 | BD Horizon | 563151 | 1:400 |  |
| CD4 | BV785 | GK1.5 | Biolegend | 100453 | 1:400 |  |
| CD4 | AF647 | GK1.5 | Biolegend | 100424 | 1:400 |  |
| CD4 | BUV737 | GK1.5 | BD Horizon | 564298 | 1:400 |  |
| CD44 | PerCP-CY5.5 | IM7 | Biolegend | 103031 | 1:400 |  |
| CD44 | V450 | IM7 | BD Horizon | 560451 | 1:400 |  |
| CD44 | AF700 | IM7 | Biolegend | 103026 | 1:400 |  |
| CD44 | PE | IM7 | BD Pharmigen | 553134 | 1:400 |  |
| CD44 | PE-Cy7 | IM7 | Thermofisher | 25-0441-82 | 1:400 |  |
| CD44 | BUV496 | IM7 | BD Horizon | 741057 | 1:400 |  |
| CD45 | V450 | 30-F11 | BD Horizon | 560501 | 1:400 |  |
| CD45 | BV605 | 30-F11 | BD Horizon | 563053 | 1:400 |  |
| CD45 | AF700 | 30-F11 | Affymetrix | 56-0451 | 1:400 |  |
| CD45 | PE | 30-F11 | Biolegend | 103106 | 1:400 |  |
| CD45 | PE-Cy7 | 30-F11 | Thermofisher | 25-0451-82 | 1:400 |  |
| CD45 | BUV395 | 30-F11 | BD Horizon | 565967 | 1:400 |  |
| CD45.1 | FITC | A20 | Biolegend | 110705 | 1:200 |  |
| CD45.1 | PE | A20 | BD Pharmigen | 553776 | 1:200 |  |
| CD45.2 | BV421 | 104 | Biolegend | 109832 | 1:100 |  |
| CD45.2 | BV785 | 104 | Biolegend | 109839 | 1:100 |  |
| CD45.2 | PE | 104 | Biolegend | 109808 | 1:100 |  |
| CD45.2 | BV395 | 104 | BD Horizon | 564616 | 1:100 |  |
| CD48 | BUV737 | HM48-1 | BD Horizon | 749666 | 1:100 |  |
| CD49α | BV711 | Ha31/8 | BD Horizon | 564863 | 1:100 |  |
| CD4-Biotin |  | GK1.5 | Thermofisher | 13-0041-85 | 1:100 | Negative beads enrichment |
| CD5 | BUV737 | 53-7.3 | BD Horizon | 565273 | 1:200 |  |
| CD5 - Biotin |  | 53-7.3 | Thermofisher | 13-0051-82 | 1:100 | Negative beads enrichment |
| CD62L | FITC | MEL-14 | Thermofisher | 11-0621-82 | 1:400 |  |
| CD62L | BUV805 | MEL-14 | BD Horizon | 741924 | 1:400 |  |
| CD64 | BV421 | X54-5/7.1 | Biolegend | 139309 | 1:100 |  |
| CD64 | BV711 | X54-5/7.1 | Biolegend | 139311 | 1:100 |  |
| CD64 | AF647 | X54-5/7.1 | BD Horizon | 558539 | 1:100 |  |
| CD68 | APC | FA-11 | Biolegend | 137007 | 1:100 |  |
| CD88 (C5ar1) | PE-Cy7 | 20/70 | Biolegend | 135810 | 1:100 |  |
| CD8α | AF700 | 53-6.7 | Biolegend | 100729 | 1:400 |  |
| CD8α | PE-Cy5 | 53-6.7 | BD Horizon | 553034 | 1:400 |  |
| CD8α - Biotin |  | 53-6.7 | Thermofisher | 13-0081-86 | 1:100 | Negative beads enrichment |
| CD8β | FITC | YTS156.7.7 | Biolegend | 126606 | 1:400 |  |
| CD8β | AF488 | YTS156.7.7 | Biolegend | 126628 | 1:400 |  |
| CD8β | BV510 | YTS156.7.7 | Biolegend | 126631 | 1:400 |  |
| CD8β | BV750 | H35-17.2 | BD Horizon | 747505 | 1:400 |  |
| CD8β | AF647 | YTS156.7.7 | Biolegend | 126612 | 1:400 |  |
| CD8β | PE-Cy7 | YTS156.7.7 | Biolegend | 126616 | 1:400 |  |
| CD90.1 (Thy1.1) | BUV737 | OX-7 | BD Horizon | 612837 | 1:200 |  |
| CD90.1 (Thy1.1) - Biotin |  | 5,00E+10 | Thermofisher | 13-0909-82 | 1:100 | Negative beads enrichment |
| CX3CR1 | FITC | SA011F11 | Biolegend | 149020 | 1:100 |  |
| CX3CR1 | PerCP-Cy5.5 | SA011F11 | Biolegend | 149009 | 1:100 |  |
| CX3CR1 | APC | SA011F11 | Biolegend | 149007 | 1:100 |  |
| CX3CR1 | PE | SA011F11 | Biolegend | 149005 | 1:100 |  |
| Dectin-2 (Clec4n) |  | D2.11E4 | Biorad | MCA2415T |  |  |
| DR3 (TNFRSF25) | PE | 4C12 | Biolegend | 144406 | 1:50 |  |
| EpCAM | AF488 | G8.8 | Biolegend | 118210 | 1:100 |  |
| EpCAM | APC | HEA-125 | Miltenyi | 130-113-260 | 1:100 |  |
| EpCAM | PE-Cy7 | G8.8 | Biolegend | 118216 | 1:100 |  |
| F4/80 | eFluor450 | BM8 | Thermofisher | 48-4801-82 | 1:200 |  |
| F4/80 | BV650 | BM8 | Biolegend | 123149 | 1:200 |  |
| F4/80 | BV785 | BM8 | Biolegend | 123141 | 1:200 |  |
| F4/80 |  | CI:A3-1 | Thermofisher | MA5-16630 | 1:100 |  |
| F4/80 | BV785 | BM8 | Biolegend | 123141 | 1:200 |  |
| F4/80 | BV650 | BM8 | Biolegend | 123149 | 1:200 |  |
| F4/80 | eFluor450 | BM8 | Thermofisher | 48-4801-82 | 1:200 |  |
| FcBlock (CD16/32) |  | 2.4G2 | BD Pharmigen | 553142 | 1:100 |  |
| GM-CSF | Purified | MP1-22E9 | Biolegend | 505401 |  |  |
| GM-CSF | APC | MP1-22E9 | Thermofisher | 17-7331-82 | 1:100 | Intracellular flow cytometry |
| GM-CSF | PE | MP1-22E9 | Biolegend | 505405 | 1:100 | Intracellular flow cytometry |
| Gr-1 | FITC | RB6-8C5 | BD Pharmigen | 553126 | 1:100 |  |
| Gr-1 | APC | RB6-8C5 | Biolegend | 108412 | 1:100 |  |
| Gr-1 | Uncojugated | RB6-8C5 | Thermofisher | MA1-10401 | 1:100 |  |
| IgG Isotype control | PE | eBio299Arm | Affymetrix | 12-4888 |  |  |
| IgG Isotype control | PE | HTK888 | Biolegend | 400907 |  |  |
| IgG1 k isotype control | PE | P3.6.2.8.1 | Affymetrix | 12-4714 |  |  |
| IgG1 k isotype control | APC | P3.6.2.8.1 | Thermofisher | 17-4714-82 |  |  |
| IgG2a isotype control | PE | 54447 | R&D systems | IC006P |  |  |
| IgG2a isotype control | APC | R35-95 | BD Pharmigen | 553932 |  |  |
| IgG2a k isotype control | APC | RTK2758 | Biolegend | 400511 |  |  |
| IgG2a k isotype control | Purified | MOPC-173 | Biolegend | 400201 |  |  |
| IL-18Rα (CD128) | AF647 | BG/IL18RA | Biolegend | 132903 | 1:100 |  |
| KLRG1 | eFluor450 | 2F1 | Thermofisher | 48-5893-82 | 1:100 |  |
| KLRG1 | BV605 | 2F1 | BD Horizon | 564013 | 1:100 |  |
| KLRG1 | APC | 2F1 | Thermofisher | 17-5893-82 | 1:100 |  |
| KLRG1 | AF561 | 2F1 | Thermofisher | 505-5893-82 | 1:100 |  |
| KLRG1 | AF647 | 2F1 | Thermofisher | 51-5893-82 | 1:100 |  |
| KLRG1 | PE-Cy5 | 2F1 | Thermofisher | 15-5893-82 | 1:100 |  |
| Ly-6A/E (Sca-1) | PerCP-Cy5.5 | D7 | Thermofisher | 45-5981-82 | 1:100 |  |
| Ly-6A/E (Sca-1) | PE | D7 | Thermofisher | 12-5981-82 | 1:100 |  |
| Ly-6A/E (Sca-1) | BUV395 | D7 | BD Horizon | 563990 | 1:100 |  |
| Ly-6C | AF488 | HK1.4 | Affymetrix | 53-5932 | 1:400 |  |
| Ly-6C | eFluor450 | HK1.4 | Affymetrix | 48-5932 | 1:400 |  |
| Ly-6C | BV605 | HK1.4 | Biolegend | 128035 | 1:400 |  |
| Ly-6C | AF700 | HK1.4 | Biolegend | 128024 | 1:400 |  |
| Ly-6D | eFluor450 | 49-H4 | Thermofisher | 12-5981-82 | 1:100 |  |
| Ly-6G | FITC | 1A8 | BD Pharmigen | 551460 | 1:200 |  |
| Ly-6G | BV785 | 1A8 | Biolegend | 127645 | 1:200 |  |
| Ly-6G | APC | 1A8 | BD Pharmigen | 560599 | 1:200 |  |
| Ly-6G | BUV395 | 1A8 | BD Horizon | 563978 | 1:200 |  |
| Ly-6G | BUV563 | 1A8 | BD Horizon | 612921 | 1:200 |  |
| MERTK | PE-Cy7 | 2B10C42 | Biolegend | 151521 | 1:100 |  |
| MHC-II | FITC | M5/114.15.2 | Affymetrix | 11-5321 | 1:600 |  |
| MHC-II | PerCP-eFluor710 | M5/114.15.2 | Affymetrix | 46-5321 | 1:600 |  |
| MHC-II | BV785 | M5/114.15.2 | BD Horizon | 743875 | 1:600 |  |
| MHC-II | AF700 | M5/114.15.2 | Thermofisher | 56-5321-82 | 1:600 |  |
| MHC-II | PE | M5/114.15.2 | Affymetrix | 12-5321 | 1:600 |  |
| MHC-II | Purified | M5/114.15.2 | Biolegend | 107601 | 1:100 |  |
| MHC-II - Biotin |  | M5/114.15.2 | Thermofisher | 107603 | 1:200 | Negative beads enrichment |
| NK1.1 | PerCP-Cy5.5 | PK136 | BD Pharmigen | 551114 | 1:100 |  |
| NK1.1 | BV605 | PK136 | BD Horizon | 563220 | 1:100 |  |
| NK1.1 | BV650 | PK136 | BD Horizon | 564143 | 1:100 |  |
| NK1.1 | APC | PK136 | BD Pharmigen | 550627 | 1:100 |  |
| NK1.1 | PE | PK136 | Biolegend | 108707 | 1:100 |  |
| NK1.1 | PE-Cy7 | PK136 | BD Pharmigen | 552878 | 1:100 |  |
| NK1.1 | BUV395 | PK136 | BD Horizon | 564144 | 1:100 |  |
| NK1.1 | BUV615 | PK136 | BD Horizon | 751111 | 1:100 |  |
| NKp46 (CD335) | BUV737 | 29A1.4 | BD Horizon | 565085 | 1:100 |  |
| ST2 (IL-33Ra) | PE-Cy7 | DIH9 | Biolegend | 145315 | 1:100 |  |
| TCRβ | BV711 | H57-597 | BD Horizon | 563135 | 1:100 |  |
| TCRβ | APC | QA18A18 | Biolegend | 159708 | 1:100 |  |
| TCRβ | PE | H57-597 | Thermofisher | 12-5961-82 | 1:100 |  |
| TCRβ | PE-CF594 | H57-597 | BD Horizon | 562841 | 1:100 |  |
| TCRβ - Biotin |  | H57-597 | Thermofisher | MA5-17550 | 1:100 | Negative beads enrichment |
| TCRδ | FITC | GL3 | BD Pharmigen | 553177 | 1:200 |  |

**Supplemental Table 2:** Complete list of reagents, key commercial kits and software used for this study.

| Chemicals, peptides and recombinant proteins | Source | Cat number |
| --- | --- | --- |
| 10x DPBS | GIBCO | 14200-083 |
| 123count eBeads | Life technologies | 01-1234-42 |
| 29G BD MicrofineTM+ Insulin syringes | BD Biosciences | 324824 |
| 5 ml Tubes Polystyrene Round-Bottom with Cell-Strainer Cap | VWR International | 734-0001 |
| ACK lysis buffer | Westburg b.v. | 10-548E |
| Azide | Sigma-Aldrich | 58032 |
| Bags, zip seal with write field 150×100 mm | VWR International | 129-0313 |
| Brefeldin A | Biolegend | 423304 |
| BSA - endotoxin free | Merck Chemicals N.V. | 126579-100GM |
| cell strainers 70µm white | VWR International | BDAA352350 |
| Collagenase A | Roche | 10103578001 |
| Collagenase D | Sigma-Aldrich | 11088866001 |
| DAPI | Life Technologies | D3571 |
| Dispase II | Sigma-Aldrich | D4693 |
| DMEM | GIBCO | 31330-038 |
| DNAse I | Sigma-Aldrich | 10104159001 |
| Dumont #5SF forceps | Fine Science Tools GmbH | 11252-00 |
| Fixable viability dye - eFluor780 | Life Technologies | 65-0865-18 |
| Fixation/Permeabilization Solution Kit | BD Pharmigen | 554714 |
| FoxP3 Transcription factor staining buffer kit | Thermofisher | 00-5523-00 |
| FTY720 | Sigma - Aldrich N.V. | SML0700 |
| gentleMACS 25 C tubes | Miltenyi Biotec | 130-093-237 |
| GM-CSF | VIB protein core |  |
| HEPES | Sigma-Aldrich | H-4034 |
| Isopore Membrane filter, PC, 0.8 µM, 13 mm | Sigma - Aldrich N.V. | ATTP01300 |
| L-Glutamine | Westburg b.v. | BE17-605F |
| Liberase™ TL Research Grade | Roche | 05401020001 |
| L-Lysine | Sigma-Aldrich | L5626 |
| LPS from Klebsiella pneumoniae | Sigma Aldrich | L-4268 |
| MagniSort™ Streptavidin Negative Selection Beads | Life technologies | MSNB-6002-74 |
| Methanol | Merck Chemicals N.V. | 13680502 |
| Monensin | Biolegend | 420701 |
| MSU crystals | Invivogen | tlrl-msu |
| Na pyruvate | Sigma-Aldrich | S-8636 |
| Non-essential aminoacids | Sigma-Aldrich | M7145 |
| nuclease free water | Life Technologies | AM9937 |
| Paraformaldehyde (4%) | VIB bioimaging core |  |
| penicillin-streptomycin | Sigma-Aldrich | P4333 |
| Percoll | VWR International | 17-0891-01 |
| Recombinant mouse TL1A/TNFSF15 protein | Biotechne - R & D Syst. Eur. | 1896-TL-010 |
| Recombinant murine IL-18 | Prepotech | B002-5 |
| Recombinant murine IL-2 | VIB protein core |  |
| Recombinant murine IL-7 | Prepotech | 217-17 |
| RPMI 1640 | Sigma-Aldrich | R1780-500ML |
| Serum | TICO International Medical | #8580166 |
| UltraComp eBeads | Life technologies | 01-2222-42 |
| UltraPure™ 0.5M EDTA, pH 8.0 | Life Technologies | 15575-020 |
| Vybrant® DiD Cell-Labeling Solution | Life Technologies | V-22887 |
| α-MEM | GIBCO | 22571-020 |
| β-mercaptoethanol | Life Technologies Europe B.V. | 31350-010 |
| Key commercial kits | Source | Cat number |
| EasySep™ Mouse Neutrophil Enrichment Kit | StemCell Technologies | 19762 |
| Magnisort mouse NK cell enrichment kit | Life technologies | 8804-6828-74 |
| CD4 (L3T4) MicroBeads mouse | Miltenyi Biotec | 130-049-201 |
| BD Cytofix/Cytoperm™ Fixation/Permeabilization Solution Kit | BD Pharmigen | 554714 |
| Direct Lineage Cell Depletion Kit | Miltenyi Biotec | 130-110-470 |
| Software/website | Source | |
| Adobe Illustrator CC EDU | Adobe (<https://www.adobe.com/>) | |
| Adobe Photoshop CC | Adobe (<https://www.adobe.com/>) | |
| Biorender | <https://www.biorender.com/> | |
| FlowJo v10.8.1 | Flowjo (<https://www.flowjo.com/>) | |
| Graphpad prism 9.4.1 | Graphpad Software Inc., La Jolla, CA (<https://www.graphpad.com/>) | |
| ImageJ | <https://imagej.nih.gov/ij/download.html> | |
| Inkscape | <https://inkscape.org/> | |
| R | <https://www.r-project.org/> | |
| R studio | <https://www.r-studio.com/> | |
| Zen Black | ZEISS Microscopy (<https://www.zeiss.com/microscopy/en/products/software/zeiss-zen-lite.html>) | |

**Supplemental Table 3:** Total-seq antibodies for CITE-seq (**figure 1f**)

| **TotalSeq** | **TotalSeq-A ID** | **Target** | **Clone** | **Isotype** |
| --- | --- | --- | --- | --- |
| A | 0001 | CD4 | RM4-5 | Rat IgG2a, κ |
| A | 0002 | CD8a | 53-6.7 | Rat IgG2a, κ |
| A | 0003 | CD366 | RMT3-23 | Rat IgG2a, κ |
| A | 0004 | CD279 | RMP1-30 | Rat IgG2b, κ |
| A | 0012 | CD117 | 2B8 | Rat IgG2b, κ |
| A | 0013 | Ly-6C | HK1.4 | Rat IgG2c, κ |
| A | 0014 | CD11b | M1/70 | Rat IgG2b, κ |
| A | 0015 | Ly-6G | 1A8 | Rat IgG2a, κ |
| A | 0070 | CD49f | GoH3 | Rat IgG2a, κ |
| A | 0073 | CD44 | IM7 | Rat IgG2a, κ |
| A | 0074 | CD54 | YN1/1.7.4 | Rat IgG2b, κ |
| A | 0076 | CD15 | MC-480 | Mouse IgM, κ |
| A | 0077 | CD73 | TY/11.8 | Rat IgG1, κ |
| A | 0078 | CD49d | R1-2 | Rat IgG2b, κ |
| A | 0079 | CD200 | OX-90 | Rat IgG2a, κ |
| A | 0090 | IgG1, κ Isotype Ctrl | MOPC-21 | Mouse (BALB/c) IgG1, Œ∫ |
| A | 0091 | IgG2a, κ Isotype Ctrl | MOPC-173 | Mouse IgG2a, Œ∫ |
| A | 0092 | IgG2b, κ Isotype Ctrl | MPC-11 | Mouse IgG2b, Œ∫ |
| A | 0095 | IgG2b, κ Isotype Ctrl | RTK4530 | Rat IgG2b, Œ∫ |
| A | 0097 | CD25 | PC61 | Rat IgG1, λ |
| A | 0098 | CD135 | A2F10 | Rat IgG2a, κ |
| A | 0104 | CD102 | 3C4 (MIC2/4) | Rat IgG2a, κ |
| A | 0106 | CD11c | N418 | Armenian Hamster IgG |
| A | 0107 | CD21,CD35 | 7E9 | Rat IgG2a, κ |
| A | 0108 | CD23 | B3B4 | Rat IgG2a, κ |
| A | 0110 | CD43 | S11 | Rat IgG2b |
| A | 0112 | CD62L | MEL-14 | Rat IgG2a, κ |
| A | 0114 | F4/80 | BM8 | Rat IgG2a, κ |
| A | 0115 | FcεRIα | MAR-1 | Armenian Hamster IgG |
| A | 0117 | I-A/I-E | M5/114.15.2 | Rat IgG2b, κ |
| A | 0118 | NK-1.1 | PK136 | Mouse IgG2a, κ |
| A | 0119 | Siglec H | 551 | Rat IgG1, κ |
| A | 0120 | TCR β chain | H57-597 | Armenian Hamster IgG |
| A | 0121 | TCR γ/δ | GL3 | Armenian Hamster IgG |
| A | 0122 | TER-119 | TER-119 | Rat IgG2b, κ |
| A | 0130 | Ly-6A/E | D7 | Rat IgG2a, κ |
| A | 0171 | CD278 | C398.4A | Armenian Hamster IgG |
| A | 0184 | CD335 | 29A1.4 | Rat IgG2a, κ |
| A | 0190 | CD274 | MIH6 | Rat IgG2a, κ |
| A | 0191 | CD27 | LG.3A10 | Armenian Hamster IgG |
| A | 0192 | CD20 | SA275A11 | Rat IgG2b, κ |
| A | 0193 | CD357 | DTA-1 | Rat IgG2b, λ |
| A | 0194 | CD137 | 17B5 | Syrian Hamster IgG |
| A | 0195 | CD134 | OX-86 | Rat IgG1, κ |
| A | 0197 | CD69 | H1.2F3 | Armenian Hamster IgG |
| A | 0198 | CD127 | A7R34 | Rat IgG2a, κ |
| A | 0200 | CD86 | GL-1 | Rat IgG2a, κ |
| A | 0201 | CD103 | 2E7 | Armenian Hamster IgG |
| A | 0203 | CD150 | TC15-12F12.2 | Rat IgG2a, λ |
| A | 0204 | CD28 | 37.51 | Syrian Hamster IgG |
| A | 0209 | TCR Vγ1.1 | 2.11 | Armenian Hamster IgG |
| A | 0210 | TCR Vγ3 | 536 | Syrian Hamster IgG |
| A | 0211 | TCR Vγ2 | UC3-10A6 | Armenian Hamster IgG |
| A | 0212 | CD24 | M1/69 | Rat IgG2b, κ |
| A | 0214 | Integrin β7 | FIB504 | Rat IgG2a, κ |
| A | 0225 | CD196 | 29-2L17 | Armenian Hamster IgG |
| A | 0226 | CD106 | 429 (MVCAM.A) | Rat IgG2a, κ |
| A | 0227 | CD122 | 5H4 | Rat IgG2a, κ |
| A | 0228 | CD183 | CXCR3-173 | Armenian Hamster IgG |
| A | 0229 | CD62P | RMP-1 | Mouse IgG2a, κ |
| A | 0230 | CD8b | YTS156.7.7 | Rat IgG2b, κ |
| A | 0232 | MAdCAM-1 | MECA-367 | Rat IgG2a, κ |
| A | 0235 | TCR Vβ8.1,8.2 | KJ16-133.18 | Rat IgG2a, κ |
| A | 0236 | IgG1, κ Isotype Ctrl | RTK2071 | Rat IgG1, Œ∫ |
| A | 0237 | IgG1, λ Isotype Ctrl | G0114F7 | Rat IgG1, Œª |
| A | 0238 | IgG2a, κ Isotype Ctrl | RTK2758 | Rat IgG2a, Œ∫ |
| A | 0240 | Rat IgG2c, κ Isotype Ctrl | RTK4174 | Rat IgG2c, Œ∫ |
| A | 0241 | IgG Isotype Ctrl | HTK888 | Armenian Hamster IgG |
| A | 0250 | KLRG1 | 2F1/KLRG1 | Syrian Hamster IgG |
| A | 0354 | TCR Vβ5.1, 5.2 | MR9-4 | Mouse IgG1, κ |
| A | 0376 | CD195 | HM-CCR5 | Armenian Hamster IgG |
| A | 0377 | CD197 | 4B12 | Rat IgG2a, κ |
| A | 0378 | CD223 | C9B7W | Rat IgG1, κ |
| A | 0379 | CD62E | RME-1/CD62E | Mouse IgG1, κ |
| A | 0381 | Pan-endothelial Cell Antigen | MECA-32 | Rat IgG2a, κ |
| A | 0388 | CD152 | UC10-4B9 | Armenian Hamster IgG |
| A | 0554 | CD309 | 89B3A5 | Rat IgG2a, κ |

**Supplemental Table 4**: Source data from **Supplemental Figure 1d**. Frequency of ILC1s (CD49α^+^ CD122^+^) in live cells in adult and neonatal thymus.

|  | Adults | Neonates |
| --- | --- | --- |
| Frequency of ILC1s (CD49a^+^ CD122^+^) in live cells | 0,052 | 0,088 |
|  | 0,039 | 0,110 |
|  | 0,035 | 0,098 |
|  | 0,066 | 0,110 |
|  | 0,051 | 0,110 |
|  | 0,110 | 0,120 |
|  | 0,079 | 0,120 |

**Supplemental Table 5**: Source data from **Supplemental Figure 1e**. Frequency of γδ T cells (CD3ε^+^ TCRγδ^+^) in live cells in adult and neonatal thymus.

|  | Adults | Neonates |
| --- | --- | --- |
| Frequency of ILC1s (CD49a^+^ CD122^+^) in live cells | 0,24 | 1,57 |
|  | 0,26 | 2,12 |
|  | 0,22 | 1,83 |
|  | 0,39 | 1,95 |
|  | 0,52 | 2,31 |
|  | 0,54 | 2,06 |
|  | 0,74 | 2,23 |

**Supplemental Table 6**: Source data from **Supplemental Figure 4b**. Body weight of neonatal mice (P7) injected with either PBS (Vehicle) or TL1A+IL-18.

| Groups | Body weight | | | | |
| --- | --- | --- | --- | --- | --- |
|  | 1^st^ injection [P3] | 2^nd^ injection [P4] | 3^rd^ injection [P5] | 4^th^ injection [P6] | Sacrifice day [P7] |
| Vehicle | 2,120 | 2,578 | 2,897 | 4,093 | 3,69 |
|  | 2,241 | 2,322 | 2,709 | 4,12 | 4,13 |
|  | 1,790 | 2,341 | 2,601 | 3,11 | 3,95 |
|  | 2,161 | 2,52 | 2,234 | 3,464 | 4,717 |
|  | 2,146 | 2,458 | 3,383 | 3,317 | 3,722 |
|  | 1,821 | 2,255 | 2,867 | 3,614 | 3,727 |
|  | 2,107 | 2,636 | 3,151 | 3,25 | 4,262 |
|  | 1,826 | 2,606 | 3,454 | 3,89 | 3,989 |
|  | 2,231 | 2,828 | 2,741 | 3,453 | 4,009 |
|  | 1,801 | 2,241 | 2,968 | 3,204 | 4,609 |
| TL1A | 2,032 | 2,617 | 3,384 | 3,807 | 4,717 |
|  | 2,317 | 2,8 | 3,414 | 3,976 | 4,532 |
|  | 2,376 | 2,645 | 3,217 | 3,646 | 4,212 |
|  | 2,087 | 2,824 | 3,125 | 3,872 | 4,274 |
| IL-18 | 2,326 | 2,545 | 3,436 | 3,888 | 4,776 |
|  | 2,171 | 2,677 | 3,437 | 3,707 | 4,676 |
|  | 2,296 | 2,728 | 3,25 | 3,935 | 4,471 |
| TL1A+IL-18 | 2,036 | 2,412 | 3,179 | 3,368 | 3,339 |
|  | 2,304 | 2,531 | 2,874 | 3,316 | 4,001 |
|  | 2,122 | 2,754 | 3,214 | 3,518 | 3,403 |
|  | 2,304 | 2,716 | 3,182 | 3,69 | 3,437 |
|  | 2,396 | 2,695 | 2,788 | 2,983 | 3,019 |
|  | 2,104 | 2,235 | 2,963 | 3,56 | 3,501 |
|  | 2,135 | 2,349 | 2,744 | 3,106 | 3,372 |
|  | 2,085 | 2,411 | 2,742 | 3,26 | 3,606 |
|  | 2,141 | 2,284 | 2,829 | 2,968 | 3,152 |
|  | 2,153 | 2,291 | 2,703 | 2,918 | 3,901 |

**Supplemental Table 7**: Source data from **Supplemental Figure 4c**. Thymic cellularity of neonatal mice (P7) injected with either PBS (Vehicle) or TL1A+IL-18.

| Samples | Beads | Live CD45^+^ cells counts | Calculated CD45^+^ cells (beads) |
| --- | --- | --- | --- |
| Thymus_Vehicle_2.fcs | 1679 | 1730000 | 16608077,16 |
| Thymus_Vehicle_3.fcs | 1861 | 1540000 | 13338232,84 |
| Thymus_Vehicle_4.fcs | 1618 | 2100000 | 20920146,63 |
| Thymus_Vehicle_5.fcs | 1523 | 1880000 | 19896738,53 |
| Thymus_Vehicle_6.fcs | 1595 | 1420000 | 14349990,18 |
| Thymus_Vehicle_7.fcs | 1267 | 1580000 | 20100386,99 |
| Thymus_Vehicle_8.fcs | 1788 | 2120000 | 19111390,8 |
| Thymus_Vehicle_9.fcs | 1600 | 1410000 | 14204405,99 |
| Thymus_Vehicle_10.fcs | 1148 | 1000000 | 14040483,35 |
| Thymus_TL1A+IL-18_1.fcs | 1644 | 1610000 | 15785124,43 |
| Thymus_TL1A+IL-18_2.fcs | 1443 | 1410000 | 15749861,11 |
| Thymus_TL1A+IL-18_3.fcs | 1776 | 1560000 | 14158119,83 |
| Thymus_TL1A+IL-18_4.fcs | 1758 | 1340000 | 12285981,99 |
| Thymus_TL1A+IL-18_5.fcs | 1830 | 1290000 | 11362203,6 |
| Thymus_TL1A+IL-18_6.fcs | 2663 | 931000 | 5635110,82 |
| Thymus_TL1A+IL-18_7.fcs | 1608 | 846000 | 8480242,382 |
| Thymus_TL1A+IL-18_8.fcs | 1743 | 1520000 | 14056271,84 |
| Thymus_TL1A+IL-18_9.fcs | 1704 | 1780000 | 16837374 |
| Thymus_TL1A+IL-18_10.fcs | 1754 | 1590000 | 14611388,29 |
| Thymus_IL-18_1.fcs | 1563 | 1650000 | 17015664,46 |
| Thymus_IL-18_2.fcs | 1614 | 1840000 | 18375460,83 |
| Thymus_IL-18_3.fcs | 1447 | 1970000 | 21944295,45 |
| Thymus_TL1A_1.fcs | 1530 | 1820000 | 19173610,64 |
| Thymus_TL1A_2.fcs | 1447 | 2000000 | 22278472,54 |
| Thymus_TL1A_3.fcs | 1177 | 1420000 | 19446248,37 |
| Thymus_TL1A_4.fcs | 1398 | 1720000 | 19831027,75 |

**Supplemental Table 8**: Source data from **Supplemental Figure 4e**. Quantification of the spleen weight of neonatal mice (P7) injected with either PBS (vehicle) or TL1A+IL-18.

| Spleen weight (mg) | |
| --- | --- |
| PBS (Vehicle) | TL1A+IL-18 |
| 24,5 | 34,7 |
| 22,2 | 40 |
| 26,4 | 36,2 |
| 35,9 | 45,5 |
| 34,2 | 36,1 |
| 31,3 | 33,3 |
| 24,8 | 33,8 |
| 24,6 | 33,5 |
| 30,9 | 36,1 |
| 34,3 | 48,7 |

**Supplemental Table 9**: Source data from **Supplemental Figure 4g**. Quantification of the spleen weight of adult mice (D5) injected with either PBS (vehicle) or TL1A+IL-18.

| Spleen weight (mg) | |
| --- | --- |
| PBS (Vehicle) | TL1A+IL-18 |
| 84,8 | 110,4 |
| 76,4 | 139,7 |
| 71,1 | 140,4 |
| 71,8 | 113,3 |
| 73 | 124,2 |
| 68,6 | 118,8 |
| 82,2 | 112,9 |
| 71,3 | 126,1 |

**Supplemental Table 10**: Source data from **Supplemental Figure 4h**. Quantification of the developing T cells subsets counts of the thymus of neonatal mice (P7) injected with either PBS (vehicle) or TL1A+IL-18.

|  | Neonates | | | | |
| --- | --- | --- | --- | --- | --- |
|  | DN1  (Lin^-^CD25^-^ CD44^+^) | DN2  (Lin^-^CD25^+^ CD44^+^) | DN3  (Lin^-^CD25^+^ CD44^-^) | DN4  (Lin^-^CD25^-^ CD44^-^) | DP  (CD4^+^CD8^+^) |
| Thymus_Vehicle_2.fcs | 4589,48 | 40993,91 | 854739,20 | 2853625,37 | 16110551,15 |
| Thymus_Vehicle_3.fcs | 3908,57 | 32880,83 | 557398,52 | 223142,62 | 15512131,61 |
| Thymus_Vehicle_4.fcs | 6709,52 | 48584,21 | 926855,56 | 1147960,11 | 23349676,31 |
| Thymus_Vehicle_5.fcs | 5537,17 | 44357,09 | 916678,34 | 2034560,56 | 21193494,62 |
| Thymus_Vehicle_6.fcs | 4289,63 | 26165,32 | 471759,66 | 429661,42 | 16388955,87 |
| Thymus_Vehicle_7.fcs | 7481,24 | 43434,25 | 1113861,51 | 4362101,00 | 18478841,60 |
| Thymus_Vehicle_8.fcs | 6076,79 | 50648,43 | 981631,07 | 2790799,43 | 19196566,86 |
| Thymus_Vehicle_9.fcs | 4247,81 | 39466,30 | 1172026,88 | 2676420,22 | 13297500,00 |
| Thymus_Vehicle_10.fcs | 5563,89 | 19701,32 | 645490,49 | 1680809,70 | 11028779,15 |
| Thymus_IL-18_1.fcs | 7872,82 | 28027,82 | 747641,49 | 5937358,42 | 12529572,54 |
| Thymus_IL-18_2.fcs | 10999,21 | 57629,68 | 956706,25 | 3416403,63 | 16618530,17 |
| Thymus_IL-18_3.fcs | 9833,77 | 43812,10 | 993980,25 | 2963042,95 | 21992451,23 |
| Thymus_TL1A_1.fcs | 8037,48 | 41182,81 | 949893,59 | 643592,76 | 21245098,04 |
| Thymus_TL1A_2.fcs | 6534,90 | 49891,45 | 992833,50 | 703679,89 | 24505874,22 |
| Thymus_TL1A_3.fcs | 8941,67 | 47759,72 | 809269,46 | 1572980,82 | 19891837,13 |
| Thymus_TL1A_4.fcs | 10975,16 | 47624,05 | 871996,53 | 1543350,94 | 19673984,81 |
| Thymus_TL1A+IL-18_1.fcs | 6304,88 | 32934,73 | 687135,67 | 1065898,86 | 17006597,42 |
| Thymus_TL1A+IL-18_2.fcs | 7277,63 | 33269,15 | 704291,54 | 815125,70 | 17170158,32 |
| Thymus_TL1A+IL-18_3.fcs | 8472,84 | 25085,76 | 670711,28 | 3857217,00 | 10008705,82 |
| Thymus_TL1A+IL-18_4.fcs | 4812,66 | 19612,89 | 405906,59 | 479648,99 | 13713497,66 |
| Thymus_TL1A+IL-18_5.fcs | 6223,01 | 21935,81 | 516907,63 | 848565,70 | 11812547,29 |
| Thymus_TL1A+IL-18_6.fcs | 3200,92 | 12137,87 | 313775,09 | 140584,22 | 6162829,66 |
| Thymus_TL1A+IL-18_7.fcs | 1865,96 | 16821,90 | 391102,11 | 272288,61 | 9216704,94 |
| Thymus_TL1A+IL-18_8.fcs | 4603,54 | 29968,62 | 946228,58 | 814069,31 | 15258175,56 |
| Thymus_TL1A+IL-18_9.fcs | 7136,71 | 33229,08 | 801686,48 | 1614791,71 | 17741738,89 |
| Thymus_TL1A+IL-18_10.fcs | 5342,31 | 28449,12 | 600193,45 | 779148,01 | 15819474,31 |

**Supplemental Table 11**: Source data from **Supplemental Figure 4i**. Quantification of the developing T cells subsets counts of the thymus of adult mice (D5) injected with either PBS (vehicle) or TL1A+IL-18.

|  | Adults | | | | |
| --- | --- | --- | --- | --- | --- |
|  | DN1  (Lin^-^CD25^-^ CD44^+^) | DN2  (Lin^-^CD25^+^ CD44^+^) | DN3  (Lin^-^CD25^+^ CD44^-^) | DN4  (Lin^-^CD25^-^ CD44^-^) | DP  (CD4^+^CD8^+^) |
| Thymus_Vehicle_2.fcs | 5614,00 | 50526,00 | 1024555,00 | 3424540,00 | 19340230,00 |
| Thymus_Vehicle_3.fcs | 5122,39 | 41466,98 | 722013,26 | 287829,61 | 20050503,48 |
| Thymus_Vehicle_4.fcs | 7438,20 | 51744,00 | 1012242,00 | 1254792,00 | 25483920,00 |
| Thymus_Vehicle_5.fcs | 6594,00 | 52752,00 | 1074822,00 | 2387028,00 | 24826410,00 |
| Thymus_Vehicle_6.fcs | 5608,40 | 34718,68 | 619594,98 | 566181,62 | 21578997,44 |
| Thymus_Vehicle_7.fcs | 8434,76 | 48662,07 | 1274946,21 | 4995972,41 | 21216662,07 |
| Thymus_Vehicle_8.fcs | 7164,50 | 59185,00 | 1133860,00 | 3208450,00 | 22147650,00 |
| Thymus_Vehicle_9.fcs | 5417,08 | 51591,26 | 1506464,79 | 3430818,79 | 17102502,69 |
| Thymus_Vehicle_10.fcs | 8695,27 | 30744,00 | 1012378,18 | 2633425,45 | 17297381,82 |
| Thymus_IL-18_1.fcs | 10171,45 | 36987,10 | 964746,77 | 7644000,00 | 16151032,26 |
| Thymus_IL-18_2.fcs | 14237,30 | 72842,00 | 1228381,00 | 4370520,00 | 21389060,00 |
| Thymus_IL-18_3.fcs | 10253,42 | 44858,71 | 1028546,13 | 3066413,23 | 22685690,32 |
| Thymus_TL1A_1.fcs | 8616,77 | 43083,87 | 1008162,58 | 683597,42 | 22575948,39 |
| Thymus_TL1A_2.fcs | 5236,19 | 39894,80 | 790415,67 | 558527,16 | 19423779,49 |
| Thymus_TL1A_3.fcs | 8169,13 | 42083,40 | 732746,22 | 1423409,05 | 17972086,44 |
| Thymus_TL1A_4.fcs | 8475,32 | 36943,71 | 680198,97 | 1201757,29 | 15299043,97 |
| Thymus_TL1A+IL-18_1.fcs | 7741,00 | 41469,65 | 851510,04 | 1321499,35 | 21094226,09 |
| Thymus_TL1A+IL-18_2.fcs | 9540,30 | 43365,00 | 919338,00 | 1063888,00 | 22463070,00 |
| Thymus_TL1A+IL-18_3.fcs | 12142,20 | 37583,00 | 968485,00 | 5579630,00 | 14455000,00 |
| Thymus_TL1A+IL-18_4.fcs | 7324,91 | 28776,44 | 614769,40 | 724643,08 | 20849838,80 |
| Thymus_TL1A+IL-18_5.fcs | 9926,23 | 35632,60 | 819549,89 | 1343858,21 | 18732568,96 |
| Thymus_TL1A+IL-18_6.fcs | 8904,25 | 33390,95 | 879294,89 | 394013,15 | 17274248,88 |
| Thymus_TL1A+IL-18_7.fcs | 2872,73 | 25136,43 | 587115,21 | 409364,73 | 13842991,63 |
| Thymus_TL1A+IL-18_8.fcs | 6292,27 | 41036,52 | 1304961,34 | 1124400,65 | 21010698,24 |
| Thymus_TL1A+IL-18_9.fcs | 9114,00 | 42532,00 | 1023806,00 | 2062802,00 | 22754620,00 |
| Thymus_TL1A+IL-18_10.fcs | 7051,99 | 37972,26 | 791992,77 | 1027963,22 | 20938986,88 |

**Supplemental table 12:** Source data from **Supplemental Figures 5a-h**. Counts of neutrophils (defined a Ly6G^+^ CD11b^+^) in the bone marrow, blood, spleen, and lungs, in neonates and adult mice injected with either PBS or TL1A + IL-18.

|  | Neonates | | | | Adults | | | |
| --- | --- | --- | --- | --- | --- | --- | --- | --- |
|  | Bone Marrow | Blood | Spleen | Lungs | Bone Marrow | Blood | Spleen | Lungs |
| Vehicle |  | 3,0E+04 | 8,0E+05 | 3,8E+04 | 1,6E+07 | 3,7E+03 | 5,1E+05 | 3,3E+05 |
|  | 3,4E+05 | 4,0E+04 | 6,7E+05 | 2,7E+04 | 1,6E+07 | 1,3E+03 | 6,1E+05 | 1,7E+05 |
|  | 2,1E+05 | 1,1E+04 | 8,3E+05 | 2,3E+04 | 1,5E+07 | 3,9E+03 | 5,6E+05 | 2,5E+05 |
|  | 2,7E+05 | 2,1E+04 | 1,1E+06 | 3,3E+04 | 1,7E+07 | 1,1E+03 | 4,8E+05 | 2,0E+05 |
|  | 5,4E+05 | 2,0E+04 | 1,3E+06 | 3,3E+04 | 1,3E+07 | 2,4E+03 | 1,3E+06 | 2,8E+05 |
|  | 4,0E+05 | 5,2E+04 | 1,4E+06 | 1,2E+05 | 1,2E+07 |  | 7,9E+05 | 3,8E+05 |
|  | 4,1E+05 | 5,3E+04 | 9,7E+05 | 7,4E+04 | 1,5E+07 |  | 1,0E+06 | 3,2E+05 |
|  | 3,6E+05 | 4,0E+04 | 1,2E+06 | 7,4E+04 | 9,3E+06 |  | 6,6E+05 | 2,7E+05 |
|  | 4,7E+05 | 1,7E+04 | 8,8E+05 | 3,7E+04 |  |  |  |  |
|  | 3,6E+05 | 3,0E+04 | 1,0E+06 | 5,2E+04 |  |  |  |  |
| TL1A + IL-18 | 6,7E+05 | 9,7E+04 | 2,2E+06 | 2,6E+05 | 9,9E+06 | 1,1E+04 | 2,7E+06 | 2,3E+05 |
|  | 1,6E+06 |  | 1,7E+06 | 2,1E+05 | 9,5E+06 | 1,6E+04 | 1,4E+06 | 4,3E+05 |
|  | 9,5E+05 | 7,8E+04 | 1,9E+06 | 2,2E+05 | 7,9E+06 | 1,6E+04 | 1,5E+06 | 4,1E+05 |
|  | 9,1E+05 | 7,2E+04 | 2,4E+06 | 2,2E+05 | 8,5E+06 | 1,4E+04 | 9,8E+05 | 4,3E+05 |
|  | 7,5E+05 | 1,1E+05 | 1,9E+06 | 1,7E+05 | 4,3E+06 | 7,3E+03 | 1,5E+06 | 3,9E+05 |
|  | 8,9E+05 | 1,8E+05 | 2,2E+06 | 1,9E+05 | 7,9E+06 | 1,1E+04 | 1,5E+06 | 5,5E+05 |
|  | 5,5E+05 | 8,2E+04 | 1,4E+06 | 1,9E+05 | 6,5E+06 | 2,1E+04 | 1,6E+06 | 2,9E+05 |
|  | 8,5E+05 | 9,4E+04 | 1,5E+06 | 2,0E+05 | 6,3E+06 | 9,4E+03 | 1,3E+06 | 2,8E+05 |
|  | 6,6E+05 | 5,0E+04 | 1,8E+06 | 1,3E+05 |  |  |  |  |
|  | 5,5E+05 |  | 1,5E+06 | 1,3E+05 |  |  |  |  |

**Supplemental Table 13**: Source data from **Supplemental Figure 7b**. Frequencies of YFP^+^ Macrophages (CD11b^+^ F4/80^+^) in the bone marrow and thymus of adult mice.

| Tissue | Sample | YFP^+^ Macrophages (%) | YFP^-^ Macrophages  (%) |
| --- | --- | --- | --- |
| Bone marrow | #1_c608_(4) Rag1-Cre tg/+ YFP tg/tg | 1,56 | 98,44 |
|  | #1_c896_(2) Rag1-Cre tg/+ YFP tg/tg | 1,96 | 98,04 |
|  | #2_c896_(3) Rag1-Cre tg/+ YFP tg/tg | 1,88 | 98,12 |
|  | #3_c892_(1) Rag1-Cre tg/+ YFP tg/tg | 1,44 | 98,56 |
|  | #1_c890_(3) Rag1-Cre tg/+ YFP tg/tg | 2,04 | 97,96 |
|  | #2_c890_(2) Rag1-Cre tg/+ YFP tg/tg | 3,79 | 96,21 |
|  | #3_c890_(1) Rag1-Cre tg/+ YFP tg/tg | 2,5 | 97,5 |
|  | #4_c890_(4) Rag1-Cre tg/+ YFP tg/tg | 1,62 | 98,38 |
|  | #7_c888_(6) Rag1-Cre tg/+ YFP tg/tg | 3,49 | 96,51 |
|  | #1_c146_(1) Rag1-Cre tg/+ YFP tg/tg | 2,22 | 97,78 |
|  | #4_c101_(5) Rag1-Cre tg/+ YFP tg/tg | 7,03 | 92,97 |
| Thymus | #1_c608_(4) Rag1-Cre tg/+ YFP tg/tg | 2,92 | 97,08 |
|  | #1_c896_(2) Rag1-Cre tg/+ YFP tg/tg | 2,63 | 97,37 |
|  | #2_c896_(3) Rag1-Cre tg/+ YFP tg/tg | 2,02 | 97,98 |
|  | #3_c892_(1) Rag1-Cre tg/+ YFP tg/tg | 3,33 | 96,67 |
|  | #1_c890_(3) Rag1-Cre tg/+ YFP tg/tg | 5,38 | 94,62 |
|  | #2_c890_(2) Rag1-Cre tg/+ YFP tg/tg | 1,32 | 98,68 |
|  | #3_c890_(1) Rag1-Cre tg/+ YFP tg/tg | 3,57 | 96,43 |
|  | #4_c890_(4) Rag1-Cre tg/+ YFP tg/tg | 15,2 | 84,8 |
|  | #5_c888_(4) Rag1-Cre tg/+ YFP tg/tg | 0,65 | 99,35 |
|  | #7_c888_(6) Rag1-Cre tg/+ YFP tg/tg | 5,04 | 94,96 |
|  | #1_c146_(1) Rag1-Cre tg/+ YFP tg/tg | 5,94 | 94,06 |
|  | #4_c101_(5) Rag1-Cre tg/+ YFP tg/tg | 8,32 | 91,68 |
|  | #2_c287(2)_Rag1-Cre +/tg YFP tg/tg | 4,01 | 95,99 |
|  | #4_c300(2)_Rag1-Cre +/tg YFP +/tg | 4,26 | 95,74 |
|  | #5_c300(3)_Rag1-Cre +/tg YFP +/tg | 4,79 | 95,21 |
|  | #7_c289(1)_Rag1-Cre +/tg YFP tg/tg | 3,48 | 96,52 |
|  | #11_c349(1)_Rag1-Cre +/tg YFP +/tg | 5,11 | 94,89 |
|  | #12_c349(3)_Rag1-Cre +/tg YFP +/tg | 4,85 | 95,15 |
|  | #13_c349(2)_Rag1-Cre +/tg YFP +/tg | 20,2 | 79,8 |

**Supplemental Table 14**: Source data from **Supplemental Figure 7f**. Counts of thymus neutrophils (defined a Ly6G^+^ CD11b^+^) and GMPs (defined a Lin- CD44^+^ c-Kit^+^ Sca-1^-^ CD34^+^ CD16/32^+^) in Rag2^-/-^ OT-I^tg/+^ adult mice.

|  | Neutrophils | GMPs |
| --- | --- | --- |
| Rag2^-/-^ OT-I ^tg/+^ | 12398,0 | 759,1 |
|  | 26755,4 | 929,0 |
|  | 22031,5 | 754,5 |
|  | 6304,3 | 326,1 |
|  | 1857,4 | 84,4 |
| Wild-types | 24699,0 | 3248,3 |
|  | 21515,7 | 2904,6 |
|  | 10057,3 | 365,7 |
|  | 8632,5 | 75,7 |
|  | 8866,0 | 0,0 |

**Supplemental Table 15:** Source data from **Supplemental Figure 8b.** Percentage of the NET forming events of NTOC- and peritoneal-derived neutrophils over 5h (300 min) incubating with either DMSO, Ionomycin, or LPS. NETotic events were defined by size exclusion (>4 µM) and sytox green signal and area.

| Time | NTOC + DMSO | | | Peritoenum + DMSO | | | NTOC + Ionomycin | | | Peritoneum + Ionomycin | | | NTOC + LPS | | | Peritoneum + LPS | | |
| --- | --- | --- | --- | --- | --- | --- | --- | --- | --- | --- | --- | --- | --- | --- | --- | --- | --- | --- |
| 0 | 4,7 | 4,3 | 1,4 | 2,8 | 2,0 | 4,1 | 3,9 | 6,5 | 3,2 | 3,0 | 2,0 | 4,1 | 3,8 | 8,4 | 9,3 | 1,8 | 1,5 | 5,8 |
| 10 | 4,7 | 5,2 | 1,4 | 4,2 | 2,7 | 5,5 | 4,4 | 6,5 | 5,3 | 3,8 | 3,0 | 6,8 | 4,3 | 7,3 | 9,3 | 2,2 | 2,7 | 7,1 |
| 20 | 4,7 | 5,2 | 1,8 | 4,2 | 3,4 | 5,9 | 4,4 | 8,1 | 6,3 | 3,8 | 4,0 | 8,8 | 5,9 | 8,4 | 10,9 | 3,1 | 2,4 | 8,4 |
| 30 | 4,7 | 4,3 | 1,8 | 7,0 | 4,1 | 5,9 | 5,3 | 8,9 | 7,4 | 3,8 | 4,0 | 8,8 | 7,5 | 9,6 | 12,4 | 6,6 | 3,6 | 8,4 |
| 40 | 4,7 | 6,0 | 2,3 | 7,0 | 4,8 | 5,9 | 6,3 | 8,9 | 7,4 | 3,8 | 4,0 | 8,8 | 8,1 | 11,2 | 11,6 | 8,3 | 4,5 | 8,8 |
| 50 | 4,7 | 5,2 | 2,3 | 8,5 | 4,8 | 5,5 | 7,8 | 10,5 | 9,5 | 4,5 | 5,0 | 9,5 | 9,7 | 10,1 | 12,4 | 8,3 | 5,7 | 9,7 |
| 60 | 4,7 | 5,2 | 2,3 | 8,5 | 4,8 | 5,9 | 8,7 | 14,5 | 10,5 | 4,5 | 5,0 | 10,1 | 10,2 | 12,4 | 12,4 | 8,8 | 5,7 | 11,1 |
| 70 | 4,7 | 6,0 | 2,3 | 8,5 | 4,8 | 5,9 | 10,2 | 15,3 | 12,6 | 5,3 | 5,9 | 10,8 | 11,3 | 12,9 | 14,7 | 12,7 | 6,3 | 13,7 |
| 80 | 5,2 | 5,2 | 2,3 | 8,5 | 5,4 | 5,9 | 12,6 | 18,5 | 14,7 | 5,3 | 6,9 | 12,8 | 12,9 | 15,7 | 18,6 | 13,6 | 8,4 | 16,8 |
| 90 | 5,7 | 7,8 | 2,7 | 8,5 | 6,1 | 5,9 | 15,0 | 21,0 | 14,7 | 6,0 | 7,9 | 13,5 | 14,5 | 16,3 | 20,9 | 15,8 | 9,6 | 19,0 |
| 100 | 5,2 | 5,2 | 3,2 | 8,5 | 6,1 | 6,2 | 17,0 | 21,8 | 16,8 | 7,5 | 8,9 | 15,5 | 16,1 | 19,7 | 24,8 | 17,1 | 12,0 | 22,1 |
| 110 | 5,2 | 4,3 | 3,6 | 8,5 | 6,1 | 6,6 | 18,9 | 22,6 | 22,1 | 9,0 | 9,9 | 14,9 | 17,7 | 23,6 | 29,5 | 18,9 | 13,2 | 26,1 |
| 120 | 5,2 | 5,2 | 3,6 | 8,5 | 7,5 | 6,6 | 22,3 | 25,0 | 25,3 | 12,8 | 12,9 | 18,2 | 22,6 | 22,5 | 32,6 | 20,6 | 14,4 | 27,4 |
| 130 | 5,2 | 4,3 | 3,6 | 8,5 | 7,5 | 7,9 | 23,3 | 25,8 | 26,3 | 12,8 | 12,9 | 19,6 | 26,3 | 23,6 | 34,9 | 21,1 | 14,7 | 29,2 |
| 140 | 5,2 | 6,9 | 3,6 | 8,5 | 7,5 | 7,6 | 26,2 | 26,6 | 28,4 | 15,8 | 15,8 | 18,9 | 27,4 | 25,8 | 39,5 | 21,1 | 15,9 | 32,3 |
| 150 | 5,2 | 6,0 | 3,6 | 8,5 | 7,5 | 7,2 | 27,7 | 27,4 | 28,4 | 17,3 | 15,8 | 18,9 | 33,3 | 28,1 | 45,7 | 21,1 | 16,2 | 35,0 |
| 160 | 5,2 | 4,3 | 3,6 | 8,5 | 7,5 | 7,6 | 30,6 | 30,6 | 28,4 | 17,3 | 16,8 | 19,6 | 33,3 | 28,7 | 46,5 | 20,2 | 16,2 | 34,5 |
| 170 | 5,2 | 6,9 | 4,1 | 9,9 | 7,5 | 7,6 | 31,1 | 30,6 | 31,6 | 19,5 | 17,8 | 20,9 | 33,3 | 29,8 | 46,5 | 20,2 | 16,5 | 36,3 |
| 180 | 5,7 | 4,3 | 4,1 | 9,9 | 8,2 | 7,2 | 30,6 | 31,5 | 31,6 | 21,8 | 20,8 | 22,3 | 35,5 | 28,1 | 45,0 | 20,2 | 18,3 | 36,7 |
| 190 | 5,7 | 6,0 | 4,1 | 9,9 | 8,2 | 7,9 | 31,6 | 31,5 | 32,6 | 21,8 | 18,8 | 23,0 | 37,1 | 30,3 | 46,5 | 21,9 | 17,1 | 38,1 |
| 200 | 6,2 | 6,0 | 4,1 | 9,9 | 8,8 | 7,9 | 31,1 | 32,3 | 34,7 | 21,1 | 19,8 | 23,0 | 38,2 | 30,3 | 48,1 | 21,9 | 17,1 | 37,6 |
| 210 | 6,7 | 6,9 | 4,5 | 9,9 | 8,8 | 7,9 | 31,1 | 33,1 | 34,7 | 22,6 | 20,8 | 24,3 | 37,6 | 30,3 | 48,8 | 21,1 | 17,1 | 38,5 |
| 220 | 7,3 | 9,5 | 4,5 | 9,9 | 9,5 | 7,9 | 31,6 | 33,1 | 34,7 | 23,3 | 20,8 | 25,7 | 38,7 | 30,9 | 50,4 | 21,1 | 17,1 | 40,7 |
| 230 | 6,7 | 7,8 | 4,5 | 9,9 | 9,5 | 7,6 | 31,6 | 32,3 | 34,7 | 24,1 | 24,8 | 28,4 | 38,7 | 32,6 | 51,2 | 21,1 | 16,2 | 38,1 |
| 240 | 6,7 | 6,9 | 4,5 | 9,9 | 9,5 | 7,2 | 34,5 | 34,7 | 36,8 | 27,1 | 26,7 | 29,1 | 39,8 | 34,8 | 51,9 | 22,4 | 17,7 | 38,5 |
| 250 | 7,3 | 7,8 | 5,0 | 9,9 | 9,5 | 7,9 | 35,4 | 33,9 | 36,8 | 29,3 | 26,7 | 30,4 | 40,3 | 33,7 | 51,9 | 24,1 | 16,2 | 39,4 |
| 260 | 7,3 | 6,9 | 5,0 | 9,9 | 10,9 | 7,6 | 36,4 | 34,7 | 36,8 | 31,6 | 30,7 | 33,1 | 39,8 | 33,1 | 52,7 | 24,1 | 16,2 | 38,5 |
| 270 | 8,3 | 7,8 | 5,0 | 9,9 | 10,9 | 7,9 | 38,8 | 36,3 | 38,9 | 34,6 | 32,7 | 35,1 | 39,8 | 33,1 | 52,7 | 26,3 | 17,7 | 39,8 |
| 280 | 8,3 | 8,6 | 5,5 | 9,9 | 10,9 | 8,6 | 39,3 | 37,1 | 38,9 | 38,3 | 33,7 | 36,5 | 38,7 | 34,3 | 52,7 | 26,3 | 18,0 | 39,8 |
| 290 | 8,3 | 8,6 | 6,4 | 11,3 | 12,2 | 9,7 | 39,3 | 40,3 | 43,2 | 43,6 | 37,6 | 38,5 | 40,3 | 35,4 | 54,3 | 27,2 | 17,4 | 39,4 |
| 300 | 8,3 | 9,5 | 6,8 | 11,3 | 12,2 | 10,3 | 41,7 | 38,7 | 46,3 | 45,1 | 46,5 | 42,6 | 40,9 | 34,8 | 54,3 | 30,7 | 17,7 | 42,0 |

**Supplemental Table 16:** Source data from **Supplemental Figure 8c.** Quantification of the NET formation of NTOC- and peritoneal-derived neutrophils after 4h (240 min) incubating with either DMSO, Ionomycin, or LPS based on the area under the curve (AUC).

|  | NTOC | Peritoneum |
| --- | --- | --- |
| DMSO | 175,9 | 260,6 |
|  | 187,1 | 222,1 |
|  | 109,5 | 212,1 |
| Ionomycin | 705,8 | 515,8 |
|  | 755,6 | 494,6 |
|  | 755,3 | 609,1 |
| LPS | 781,5 | 533,3 |
|  | 714,3 | 382,9 |
|  | 1061 | 835 |

**Supplemental Table 17**: Source data from **Supplemental Figure 9a**. Multiplex cytokines measurements in the NTOC supernatant after 6 days of treatment with different cytokine combinations.

|  | IL-22 (pg/ml) | | | |
| --- | --- | --- | --- | --- |
|  | Vehicle | TL1A | IL-18 | TL1A+IL-18 |
| Rep 1 | 10,39 | 20,19 | 85,99 | 716,68 |
| Rep 2 | 13,15 | 54,38 | 206,79 | 1061,11 |
| Rep 3 | 4,23 | 59,51 | 80,53 | 498,29 |
| Rep 4 | 5,57 | 30,34 | 114,47 | 404,44 |
| Rep 5 | 53,05 | 55,53 | 798,27 | 407,69 |
| Rep 6 | 45,14 | 51,72 | 301,78 | 591,87 |
|  | IL-5 (pg/ml) | | | |
|  | Vehicle | TL1A | IL-18 | TL1A+IL-18 |
| Rep 1 | 34,36 | 82,76 | 72,13 | 718,61 |
| Rep 2 | 36,75 | 330,03 | 116,02 | 776,88 |
| Rep 3 | 22,92 | 114,21 | 45,94 | 502,48 |
| Rep 4 | 31,92 | 117,83 | 62,91 | 499,60 |
| Rep 5 | 47,38 | 125,25 | 115,32 | 507,04 |
| Rep 6 | 0,07 | 91,43 | 61,53 | 713,16 |
|  | TNFα (pg/ml) | | | |
|  | Vehicle | TL1A | IL-18 | TL1A+IL-18 |
| Rep 1 | 180,67 | 311,95 | 744,52 | 1056,08 |
| Rep 2 | 321,03 | 618,83 | 637,71 | 1038,59 |
| Rep 3 | 214,71 | 481,90 | 364,94 | 657,80 |
| Rep 4 | 143,93 | 421,57 | 563,67 | 689,60 |
| Rep 5 | 378,55 | 327,28 | 761,86 | 770,69 |
| Rep 6 | 409,17 | 356,97 | 728,22 | 873,42 |
|  | IL-10 (pg/ml) | | | |
|  | Vehicle | TL1A | IL-18 | TL1A+IL-18 |
| Rep 1 | 61,59 | 45,47 | 178,53 | 51,39 |
| Rep 2 | 110,57 | 55,32 | 134,89 | 56,89 |
| Rep 3 | 132,62 | 59,24 | 77,53 | 39,92 |
| Rep 4 | 107,52 | 54,54 | 115,15 | 37,53 |
| Rep 5 | 136,78 | 52,96 | 156,78 | 69,77 |
| Rep 6 |  | 75,21 | 106,37 | 82,17 |
|  | IL-6 (pg/ml) | | | |
|  | Vehicle | TL1A | IL-18 | TL1A+IL-18 |
| Rep 1 | 4337,47 | 3145,73 | 4072,70 | 12422,96 |
| Rep 2 | 4433,00 | 8046,62 | 8309,95 | 19022,41 |
| Rep 3 | 2456,83 | 2750,55 | 3616,63 | 6443,72 |
| Rep 4 | 1939,53 | 1947,78 | 5445,84 | 4870,25 |
| Rep 5 | 8624,22 | 5755,92 | 28593,20 | 11363,56 |
| Rep 6 | 10753,35 | 7814,17 | 21104,94 | 12914,36 |

**Supplemental Table 18**: Source data from **Supplemental Figure 12b**. Neutrophil counts in the NTOCs lobes or C57BL/6J treated with anti-GM-CSFR antibody.

| Samples and treatment | Neutrophils counts [verse] (CD11b^+^ Ly-6G^+^) |
| --- | --- |
| NTOC Lobes day 6_Vehicle_rep1.fcs | 599689,0625 |
| NTOC Lobes day 6_Vehicle_rep2.fcs | 485030 |
| NTOC Lobes day 6_Vehicle_rep3.fcs | 471301,6129 |
| NTOC Lobes day 6_Vehicle_rep4.fcs | 684973,3333 |
| NTOC Lobes day 6_TL1A+IL-18_rep1.fcs | 172464,5161 |
| NTOC Lobes day 6_TL1A+IL-18_rep2.fcs | 246448,4375 |
| NTOC Lobes day 6_TL1A+IL-18_rep3.fcs | 220540,3226 |
| NTOC Lobes day 6_TL1A+IL-18_rep4.fcs | 218093,5484 |
| NTOC Lobes day 6_TL1A+IL-18+ 200ng,2f,mL anti-GM-CSF_rep1.fcs | 244479,0323 |
| NTOC Lobes day 6_TL1A+IL-18+ 200ng,2f,mL anti-GM-CSF_rep2.fcs | 326743,5484 |
| NTOC Lobes day 6_TL1A+IL-18+ 200ng,2f,mL anti-GM-CSF_rep3.fcs | 231265,625 |
| NTOC Lobes day 6_TL1A+IL-18+ 500ng,2f,mL anti-GM-CSF_rep1.fcs | 235621,875 |
| NTOC Lobes day 6_TL1A+IL-18+ 500ng,2f,mL anti-GM-CSF_rep2.fcs | 146767,1875 |
| NTOC Lobes day 6_TL1A+IL-18+ 500ng,2f,mL anti-GM-CSF_rep3.fcs | 227550 |
| NTOC Lobes day 6_TL1A+IL-18+ 1ug,2f,mL anti-GM-CSF_rep1.fcs | 175210,9375 |
| NTOC Lobes day 6_TL1A+IL-18+ 1ug,2f,mL anti-GM-CSF_rep2.fcs | 165345,3125 |
| NTOC Lobes day 6_TL1A+IL-18+ 1ug,2f,mL anti-GM-CSF_rep3.fcs | 186934,375 |
| NTOC Lobes day 6_TL1A+IL-18+ 2ug,2f,mL anti-GM-CSF_rep1.fcs | 106732,2581 |
| NTOC Lobes day 6_TL1A+IL-18+ 2ug,2f,mL anti-GM-CSF_rep2.fcs | 120222,5806 |
| NTOC Lobes day 6_TL1A+IL-18+ 2ug,2f,mL anti-GM-CSF_rep3.fcs | 59770,3125 |

**Supplemental Table 19**: Source data from **Supplemental Figure 12c**. Neutrophil counts in NTOCs performed with either TCRd^-/-^ thymic lobes or C57BL/6J (control). We depicted the neutrophils numbers inside lobes and in the supernatant following 6 days of treatment with either vehicle (control) or TL1A+IL-18.

|  | TCRd^-/-^ | | C57BL/6J | |
| --- | --- | --- | --- | --- |
|  | Vehicle | TL1A+IL-18 | Vehicle | TL1A+IL-18 |
| NTOC Lobes day 6 | 921,77 | 21662,86 | 517,82 | 8180,96 |
|  | 838,76 | 9591,02 | 1191,97 | 4472,45 |
|  | 374,24 | 17615,88 | 707,00 | 4363,68 |
|  | N/A | 9117,56 | 511,46 | 10555,00 |
|  | N/A | N/A | 1184,01 | 11394,12 |
| NTOC supernatant day 6 | 1364,05 | 223720,65 | 492,35 | 99912,69 |
|  | 1611,81 | 157685,90 | 467,95 | 127319,22 |
|  | 563,94 | 375416,69 | 359,50 | 117636,83 |
|  | N/A | 470124,59 | 1310,16 | 287626,11 |
|  | N/A | N/A | 814,06 | 105949,67 |

**Supplemental Table 20**: Source data from **Supplemental Figure 13a**. Total protein levels in the thymus of adult mice either mock-, or PVM-infected after 5, 10 or 14 days post-infection.

|  | Day 5 | Day 10 | Day 14 |
| --- | --- | --- | --- |
| Control | 1595,3 | 2514,2 | 3148,2 |
|  | 2588,0 | 2055,6 | 2218,5 |
|  | 2011,1 | 2092,6 | 2506,8 |
|  | 1222,5 | 2971,5 | 2919,9 |
|  | 1602,7 | 2669,2 | 3177,6 |
|  |  | 3118,8 | 2425,5 |
| PVM | 1543,2 | 493,2 | 1356,9 |
|  | 1773,7 | 719,9 | 485,6 |
|  | 1639,9 | 599,2 | 1057,9 |
|  | 2196,3 | 892,9 |  |
|  | 1438,9 | 553,8 |  |
|  |  | 636,9 |  |

**Supplemental Table 21**: Source data from **Supplemental Figure 13b**. Total protein levels in the thymus of adult mice either mock-, or MCMV-infected after 2, 5 or 8 days post-infection.

|  | Day 2 | Day 5 | Day 8 |
| --- | --- | --- | --- |
| Control | 1862,8 | 2151,8 | 2418,1 |
|  | 2033,3 | 1192,6 | 2055,6 |
|  | 2558,5 | 1951,8 | 1453,8 |
|  | 3148,2 | 2248,1 | 2351,6 |
|  | 3074,6 | 1988,9 | 2314,6 |
|  | 2602,8 | 2462,5 | 1654,8 |
| MCMV | 3008,3 | 500,7 | 1461,2 |
|  | 2314,6 | 636,9 | 825,3 |
|  | 2536,4 | 599,2 | 2270,3 |
|  | 2669,2 | 295,3 | 2573,3 |
|  | 3037,8 | 379,2 | 2100,0 |
|  | 2565,9 | 2225,9 | 750,0 |

**Supplemental Table 22**: Source data from **Supplemental Figure 13c**. Quantification of the proportion of neutrophils out of CD45^+^ cells in the thymuses of mock- (grey), or PVM- (purple) infected mice at day 5, 10, and 14 of infection. Data representative of one of two experiments.

|  | Day 5 | Day 10 | Day 14 |
| --- | --- | --- | --- |
| Control | 0,008 | 0,009 | 0,014 |
|  | 0,017 | 0,012 | 0,007 |
|  | 0,015 | 0,027 | 0,011 |
|  | 0,016 | 0,013 | 0,010 |
|  | 0,009 | 0,020 | 0,007 |
|  | 0,008 | 0,022 | 0,015 |
| PVM | 0,019 | 0,180 | 0,080 |
|  | 0,015 | 0,180 | 0,013 |
|  | 0,007 | 0,130 | 0,045 |
|  | 0,024 | 0,043 |  |
|  | 0,019 | 0,190 |  |
|  | 0,015 | 0,140 |  |

**Supplemental Table 23**: Source data from **Supplemental Figure 13d**. Quantification of the neutrophil numbers in the thymuses of mock- (grey), or PVM- (purple) infected mice at day 5, 10, and 14 of infection. Data representative of one of two experiments.

|  | Day 5 | Day 10 | Day 14 |
| --- | --- | --- | --- |
| Control | 13516,6 | 11284,9 | 20788,1 |
|  | 30182,1 | 32018,2 | 17991,7 |
|  | 27821,5 | 19712,2 | 17778,8 |
|  | 23884,6 | 23917,4 | 14500,8 |
|  | 13161,7 | 22434,7 | 10701,3 |
|  | 16011,3 | 38755,8 | 20855,4 |
| PVM | 31511,8 | 6432,9 | 5496,8 |
|  | 22606,7 | 39459,1 | 2192,6 |
|  | 10637,4 | 11615,7 | 1362,3 |
|  | 29876,6 | 12795,9 |  |
|  | 20096,6 | 14636,6 |  |
|  | 16552,4 | 13957,7 |  |

**Supplemental Table 24**: Source data from **Supplemental Figure 13e**. Quantification of the proportion of neutrophils out of CD45^+^ cells in the thymuses of mock- (grey), or MCMV- (blue) infected mice at day 5, 10, and 14 of infection.

|  | Day 2 | Day 5 | Day 8 |
| --- | --- | --- | --- |
| Control | 0,024 | 0,021 | 0,017 |
|  | 0,038 | 0,028 | 0,022 |
|  | 0,029 | 0,039 | 0,012 |
|  | 0,064 | 0,034 | 0,018 |
|  | 0,036 | 0,042 | 0,046 |
|  |  | 0,031 | 0,011 |
| MCMV | 0,069 | 0,730 | 0,230 |
|  | 0,040 | 0,350 | 0,350 |
|  | 0,018 | 0,430 | 0,022 |
|  | 0,088 | 0,510 | 0,010 |
|  | 0,037 | 1,250 | 0,010 |
|  |  | 0,022 | 0,140 |

**Supplemental Table 25**: Source data from **Supplemental Figure 13f**. Quantification of the neutrophil numbers in the thymuses of mock- (grey), or MCMV- (blue) infected mice at day 2, 5, and 8 of infection. Data representative of one of two experiments.

|  | Day 2 | Day 5 | Day 8 |
| --- | --- | --- | --- |
| Control | 18382,2 | 34802,1 | 15808,7 |
|  | 25273,7 | 39963,0 | 17773,0 |
|  | 9387,9 | 31859,0 | 3527,8 |
|  | 25942,0 | 26204,0 | 16208,5 |
|  | 16211,1 | 68406,6 | 41345,1 |
|  |  | 44039,2 | 10515,2 |
| MCMV | 26415,1 | 10394,7 | 17661,2 |
|  | 13530,7 | 10794,9 | 13500,2 |
|  | 11231,6 | 9130,6 | 17288,8 |
|  | 29551,6 | 21894,7 | 10218,2 |
|  | 9797,1 | 12714,8 | 8871,6 |
|  |  | 15975,2 | 5496,7 |

**Supplemental Table 26**: Source data from **Supplemental Figure 13g**. Quantification of the proportion of neutrophils out of CD45^+^ cells in the blood of mock- (grey), or PVM- (purple) infected mice at day 2, 5, and 8 of infection. Data representative of one of two experiments.

|  | Day 5 | Day 10 | Day 14 |
| --- | --- | --- | --- |
| Control | 6,1 | 4,4 | 12,1 |
|  | 14,3 | 11,6 | 4,6 |
|  | 4,6 | 13,9 | 6,1 |
|  | 4,8 | 14,2 | 11,9 |
|  | 6,6 | 6,9 | 11,2 |
|  | 0,6 | 11,0 | 7,3 |
| PVM | 10,2 | 30,2 | 8,4 |
|  | 2,9 | 15,2 | 7,1 |
|  | 4,5 | 28,8 | 9,0 |
|  | 2,6 | 39,4 |  |
|  | 0,7 | 33,1 |  |
|  | 1,0 | 13,0 |  |

**Supplemental Table 27**: Source data from **Supplemental Figure 13h**. Quantification of the neutrophils numbers cells in the blood of mock- (grey), or PVM- (purple) infected mice at day 2, 5, and 8 of infection. Data representative of one of two experiments.

|  | Day 5 | Day 10 | Day 14 |
| --- | --- | --- | --- |
| Control | 12703,4 | 5770,0 | 7030,3 |
|  | 29591,8 | 32812,0 | 1191,3 |
|  | 1144,0 | 34495,7 | 2125,2 |
|  | 8861,6 | 24637,2 | 22328,8 |
|  | 12588,2 | 44889,9 | 24941,8 |
|  | 722,5 | 28168,1 | 1991,0 |
| PVM | 12364,7 | 25220,6 | 61110,6 |
|  | 4182,4 | 38354,1 | 6513,8 |
|  | 8659,6 | 45922,8 | 54680,5 |
|  | 1826,8 | 60437,0 |  |
|  | 497,8 | 39591,0 |  |
|  | 423,6 | 46956,8 |  |

**Supplemental Table 28**: Source data from **Supplemental Figure 13g**. Quantification of the proportion of neutrophils out of CD45^+^ cells in the blood of mock- (grey), or MCMV- (blue) infected mice at day 2, 5, and 8 of infection. Data representative of one of two experiments.

|  | Day 2 | Day 5 | Day 8 |
| --- | --- | --- | --- |
| Control | 1,0 | 15,2 | 12,4 |
|  | 1,1 | 13,5 | 19,9 |
|  | 1,0 | 6,8 | 14,1 |
|  | 2,2 | 6,8 | 12,0 |
|  | 0,8 | 10,1 | 16,7 |
|  | 1,2 | 10,9 | 6,1 |
| MCMV | 8,0 | 47,1 | 12,1 |
|  | 1,2 | 39,2 | 11,9 |
|  | 12,6 | 36,5 | 9,5 |
|  | 5,6 | 30,6 | 9,8 |
|  | 4,4 | 41,7 | 1,5 |
|  |  | 14,7 | 10,5 |

**Supplemental Table 29**: Source data from **Supplemental Figure 13j**. Quantification of the neutrophils counts in the blood of mock- (grey), or MCMV- (blue) infected mice at day 2, 5, and 8 of infection. Data representative of one of two experiments.

|  | Day 2 | Day 5 | Day 8 |
| --- | --- | --- | --- |
| Control | 463,1 | 36797,1 | 17486,1 |
|  | 188,1 | 28762,9 | 45605,3 |
|  | 328,0 | 15993,5 | 42786,1 |
|  | 453,6 | 12025,0 | 23108,4 |
|  | 143,2 | 19343,5 | 15151,2 |
|  | 222,6 | 17186,4 | 2196,8 |
| MCMV | 4095,1 | 110296,6 | 154134,5 |
|  | 444,0 | 399020,9 | 179193,0 |
|  | 27353,8 | 455417,3 | 38344,7 |
|  | 749,6 | 245853,5 | 25791,2 |
|  | 2898,4 | 647796,5 | 5862,5 |
|  |  | 38407,3 | 237197,0 |

**Supplementary Figures Legends**

**Supplemental Figure 1: Characterization of DR3 and IL-18R expression in neonatal and adult murine thymus**

**(a)** Flow cytometric characterization of DR3 expression on different thymocyte subsets in the thymus of adult (12 weeks old) and neonatal mice (P0.5). (n = 5). The histograms display fluorescence intensity of DR3 (X axis) across different thymocyte subsets set in modal (Y axis). Data is representative of 5 independent experiments.

**(b)** Flow cytometric characterization of IL-18Rα expression on different thymocyte subsets in the thymus of adult (12 weeks old) and neonatal mice (P0.5). (n = 5). The histograms display fluorescence intensity of IL-18Rα (X axis) across different thymocyte subsets set in modal (Y axis). Data is representative of 5 independent experiments.

**(c)** Gating strategy used to define the different thymocyte subsets in both adult and neonatal thymus from A&B. This gating has been used consistently across different experiments.

**(d)** Differences in the proportion of γδT cells (CD3ε^+^TCRγδ^+^) between adult and neonatal thymus by flow cytometry. (n = 5). Data representative of one of at least three independent experiments.

**(e)** Differences in the proportion of ILC1s (CD3ε^-^TCRγδ^-^CD62L^-^CD49α^+^CD122^+^) between adult and neonatal thymus by flow cytometry. (n = 5). Data representative of one of at least three independent experiments.

**Supplemental Figure 2: TEM shows TL1A+IL-18-induced thymic atrophy**

**(a)** TEM Images acquired from NTOC lobes treated during 6 days with either Vehicle (PBS, left panel) or TL1A+IL-18 (right panel) acquired as described above in supplemental methods.

**Supplemental Figure 3: CITE-seq ADT data**

**(a)** Feature plots of genes (*Cd4*, *Cd86*, *H2-Ab1* and *Itgam*) and corresponding antibody-derived tags (ADT, protein level) expression (CD4, CD86, MHC-II and CD11b) on the thymic egressing cells from the organ culture (**Figure 1f**) by CITE-seq.

**(b)** Heatmap of the protein expression (ADT) of all the antibodies used in the library for our CITE-seq.

**Supplemental Figure 4: Effect of TL1A and IL-18 administration in neonatal and adult mice**

**(a)** Images of the sizes of neonatal mice injected with the different cytokine treatments the last day of the injection model (P7) (**Figure 2q**). (n = 10 for the Vehicle (PBS) and TL1A+IL-18 groups, n = 4 for TL1A-injected group and lastly, n = 3 for IL-18-injected pups). Data representative of one of at least 3 independent experiments.

**(b)** Quantification of the body weight of neonatal mice injected with the different cytokine treatments.

**(c)** Corresponding quantification of the thymocytes counts in the thymus of neonatal pups (P5) injected with the different cytokine treatments. Thymocytes counts were calculated based of e123 counting beads (Thermofisher, #01-1234-42).

**(d)** Pictures of the spleen of pups (P7) injected with either PBS (vehicle) or TL1A+IL-18 combo.

**(e)** Corresponding quantification of the neonates spleen weight (shown in **figure S2d**).

**(f)** Pictures of the spleen of adults (D5) injected with either PBS (vehicle) or TL1A+IL-18 combo.

**(g)** Corresponding quantification of the adults spleen weight (shown in figure S2f).

**(h)** Representation of the numbers of the different pre-T cell subsets across the T cell developmental pathway (From DN1 to DP) in neonates injected with either PBS (vehicle) or TL1A+IL-18 combo. The counts of the different subsets were calculated based on BD FACSVerse™ Cell Analyzer. DN4 cells were the main subset affected by the treatment. (n = 10). Data representative of one out of 5 independent experiments.

**(i)** Quantification of the counts of the different pre-T cell subsets across the T cell developmental pathway (From DN1 to DP) in adults injected with either PBS (Sham) or TL1A+IL-18 combo. The counts of the different subsets were calculated based on BD FACSVerse™ Cell Analyzer. DP cells were the main subset affected by the treatment. (n = 10). Data representative of one out of 5 independent experiments.

**Statistics:** (a and c) One-way ANOVA, (e, g, h and i) Unpaired T-test with welch correction. * p < 0.05, ** p < 0.01, *** p < 0.001, **** p < 0.0001.

**Supplemental Figure 5: *In vivo* administration of TL1A and IL-18 leads to systemic neutrophilia in both neonatal and adult mice**

**(a-d)** Quantification of the neutrophil numbers in the bone marrow, blood, spleen and lungs of neonatal mice (P7) either injected with PBS (grey) or TL1A+IL-18 (red). Neutrophils were defined as Lin^-^CD11b^+^Ly-6G^+^. Data representative of one of at least five independent experiments (n = 8).

**(e-h)** Quantification of the neutrophil numbers in the bone marrow, blood, spleen and lungs of adult mice (D5) either injected with PBS (grey) or TL1A+IL-18 (red). Neutrophils were defined as Lin^-^CD11b^+^Ly-6G^+^. Data representative of one of at least five independent experiments (n = 10).

**Supplemental Figure 6: scRNAseq identifies transcriptional changes between different NTOC-derived neutrophils at different developmental stages and between treatments**

**(a)** Vulcano plots of differential expressed genes between the three different neutrophil clusters displayed in **figure 1f** (“pre-neutrophils”, “immature neutrophils” and “mature neutrophils”) defined by single-cell RNAseq.

**(b)** Vulcano plots of the differential expressed genes of the three different neutrophil clusters displayed **figure 1f** (“pre-neutrophils”, “immature neutrophils” and “mature neutrophils”) between treatments by single-cell RNAseq.

**(c)** Pseudotime analysis (“Slingshot” package) of genes and transcription factors involved in thymic neutrophil development, similarly as shown in **figure 3c**.

**(d)** Violin plots representing the gene expression level of transcription factors controlling T- or Myeloid development respectively for the scRNAseq dataset displayed in figure **1f-h**.

**Supplemental Figure 7: Additional information about the *Rag1*-Cre Rosa26YFP and *Ms4a3*-Cre Rosa26Tdtomato fate-mapping models**

**(a)** Genetic construction of the *Rag1*-Cre genetic mouse model. *Rag1*-Cre was generated through targeted gene knock-in, where the Cre recombinase was inserted into the Rag1 exon. To achieve this, a *Rag1* targeting vector was constructed using a SmaI-NdeI fragment encompassing the *Rag1* coding exon. Additionally, a BamHI site was introduced into exon 1 of *Rag1* (denoted as B*), and the Cre cDNA was seamlessly integrated into the *Rag1* coding sequence. The simian virus 40 (SV40) polyadenylation site was positioned downstream of Cre, while the neomycin resistance cassette MC1-neo-pA (Neo) was inserted further downstream. This construct was employed to target one endogenous *Rag1* allele through homologous recombination, resulting in the generation of embryonic stem cells possessing a single targeted *Rag1* allele. Subsequently, mice were derived from these embryonic stem cells, and they exhibited consistent hematopoiesis without any observable variations^9^.

**(b)** Flow cytometric characterization of the history expression of *Rag1* in macrophages (defined as CD11b^+^ F4/80^+^) from the bone marrow and thymus of adult mice (8 weeks old).

**(c)** Flow cytometric characterization of the different progenitors populations found in the adult bone marrow. Thymuses from (8-weeks old) *Rag1*-Cre+/tg Rosa26YFP+/tg mice were isolated and subjected to negative bead depletion with CD4-Biotin (1:50) (Thymuses) or CD19-Biotin (1:100), CD11b-Biotin (1:100), Ter-119-Biotin (1:200) and CD3-Biotin (1:200). All the antibodies are listed in **Supplemental table 1**. We defined progenitors in the LSK gate (Lin^-^ Sca-1^+^ and c-Kit^+^, in brown) and Lin^-^c-kit^+^Sca-1^-^ progenitors. LSK cells were divided into common-lymphoid progenitors (**CLPs),** defined as Lin^-^CD44^+^Sca-1^+^c-Kit^+^IL-7Rα^+^CD34^-^ (in green) and Lympho-myeloid primed progenitors **(LMPPs),** defined as Lin^-^CD44^+^Sca-1^+^c-Kit^+^IL-7Rα^-^CD34^+^ (in orange). Granulocyte-monocyte progenitors **(GMPs),** were defined as Lin^-^Sca-1^-^c-Kit^+^CD16/32^+^CD34^+^ (in red).

**(d)** ATAC-seq data from **Ferreira *et al.* 2021**^5^, showing an open cremating region in the Rag1 locus (Chromosome 2, 101 470 448 position) coinciding with the transcription start site (TSS) according to the transcription start database refTSS4 (<https://reftss.riken.jp/>), version 38 of the mouse genome.

**(e)** Characterization of the history expression of *Ms4a3* in the adult thymus (8 weeks old). (n = 8). Data representative of one out of two independent experiments.

**(f)** Quantification of the neutrophil (CD11b^+^Ly-6G^+^) and GMPs (Lin^-^Sca-1^-^c-Kit^+^CD16/32^+^CD34^+^) numbers in the thymus of *Rag2*^-/-^ OT-I^tg/+^ mice compared to aged and gender matched wild-type mice. (n = 5)

**Statistics:** (b,f) One-way ANOVA, * p < 0.05, ** p < 0.01, *** p < 0.001, **** p < 0.0001.

**Supplemental Figure 8: Phagocytosis images, NETs quantification, MMPs expression and Gene ontology (GO) analysis and transcriptional profile of the three different subsets of NTOC-derived neutrophils**

**(a)** Pictures of the phagocytic events of NTOC- and peritoneal-derived neutrophils after 10, 20, and 30 min incubating with PE-pHrodo particles from *S.Aurueus* corresponding with the data displayed in **figure 5d**.

**(b)** Kinetics of the NET forming events of NTOC- and peritoneal-derived neutrophils over 5h (300 min) incubating with either DMSO, Ionomycin, or LPS. NETotic events were defined by size exclusion (>4 µM) and sytox green signal and area. Data representative of one of three experiments. Error bars represent SEM.

**(c)** Quantification of the NET formation of NTOC- and peritoneal-derived neutrophils after 4h (240 min) incubating with either DMSO, Ionomycin, or LPS based on the area under the curve (AUC). Data representative of one of three experiments. Error bars represent SEM.

**(d)** Dot plot representing the gene expression of metalloproteases family in thymic myeloid cells for the scRNAseq dataset displayed in **figure 1f-h**.

**(e)** Violin plot with the average expression of *Mmp8* and *Mmp9* normalized per cell counts in neutrophils across the different treatments for the scRNAseq dataset represented in **figure 1f**.

**(f)** Violin plot that displays the transcriptional profile of thymic neutrophils subsets defined in **figure 1f**, **1g** and **3b**.

**(g)** Gene ontology (GO) analysis of the three neutrophil clusters defined by scRNAseq in **figure 1F**. Differentially expressed genes in the proliferative Neutrophils (pNeu), immature neutrophils (iNeu) and mature neutrophils (mNeu) clusters were compared to each other using gProfiler2. Pathway enrichment is expressed as the –log[p.value] adjusted for multiple comparisons.

**Statistics:** (c) Unpaired T-test with welch correction. * p < 0.05, ** p < 0.01, *** p < 0.001, **** p < 0.0001.

**Supplemental Figure 9: Further information about the mechanism of thymic neutrophil expansion in response to TL1A+IL-18 treatment**

**(a)** Multiplex cytokine measurement in the NTOC supernatant at day 6 of culture treated with: (1) Vehicle (green), (2) TL1A (blue), (3) IL-18 (yellow), and (4) TL1A+IL-18 (red). From left to right, we measured IL-22, IL-5, TNFα, IL-10 and IL-6. (n = 6). Results shown pooled from three independent experiments.

**(b)** Deconvoluted UMAPs of the 5 different samples that compose the aggregate shown in (G**)**. We defined 19 clusters were grouped in four metaclusters: T cells (green), Monocytes/Macrophages (blue), Neutrophils (orange/red), ILCs (purple), pDCs (yellow) and epithelial cells (brown). (1) Convoluted UMAP of all samples in the aggregate, (2) Neonatal thymuses (P0.5), (3) NTOC vehicle - day 1.5, (4) NTOC TL1A+IL-18 - day 1.5, (5) NTOC vehicle - day 3, and (6) NTOC TL1A+IL-18 - day 3.

**Supplemental Figure 10: Cluster annotation of the scRNAseq dataset shown in Figure 6c by manually curated DE genes**

**(a)** Heatmap of the manually curated genes that define the cellular identity of the 19 clusters shown in **Figure 6c**.

**Supplemental Figure 11: NicheNet analysis of the top predicted ligand-receptors interaction between ILCs/γδT cells (senders) and neutrophils (receivers)**

**(a)** NicheNet analysis of the predicted ligand-receptor pair interactions between γδ T cells and ILCs (senders) and Neutrophils (receivers) divided by samples: (1) Neonatal thymuses (P0.5), (2) NTOC vehicle - day 1.5, (3) NTOC TL1A+IL-18 - day 1.5, (4) NTOC vehicle - day 3, and (5) NTOC TL1A+IL-18 - day 3.

**Supplemental Figure 12: Extended data about the thymic source of GM-CSF in response to combined treatment with TL1A and IL-18.**

**(a)** Flow cytometry histograms of the GM-CSF production of the different thymic subsets inside the lobes at day 3 of the NTOC treated with either vehicle or TL1A+IL-18.

**(b)** Flow cytometry counts of neutrophils in the NTOC lobes at day 6 of the culture when treated with anti-GM-CSFR antibodies.

**(c)** Comparison of the neutrophil counts in the NTOC in response to the treatment with either vehicle (control) or TL1A+IL-18 in C57BL/6N mice (control) versus TCRd^-/-^ mice to determine whether γδ T cells are an essential source of GM-CSF driving the expansion of thymic neutrophils in response to TL1A+IL-18.

**Statistics:** (a and f) One-way ANOVA, (G) 2-way ANOVA. * p < 0.05, ** p < 0.01, *** p < 0.001, **** p < 0.0001.

**Supplemental Figure 13: Extended data about the PVM, and MCMV infection models in the thymus and blood.**

**(a)** Quantification of the protein content in the thymuses of mock- (grey), or PVM- (purple) infected mice at day 5, 10, and 14 of infection.

**(b)** Quantification of the protein content in the thymuses of mock- (grey), or MCMV- (blue) infected mice at day 2, 5, and 8 of infection.

**(c)** Quantification of the neutrophil numbers in the thymuses of mock- (grey), or PVM- (purple) infected mice at day 5, 10, and 14 of infection.

**(d)** Quantification of the proportion of neutrophils out of CD45^+^ cells in the thymuses of mock- (grey), or PVM- (purple) infected mice at day 5, 10, and 14 of infection.

**(e)** Quantification of the neutrophil numbers in the thymuses of mock- (grey), or MCMV- (blue) infected mice at day 2, 5, and 8 of infection.

**(f)** Quantification of the proportion of neutrophils out of CD45^+^ cells in the thymuses of mock- (grey), or MCMV- (blue) infected mice at day 2, 5, and 8 of infection.

**(g)** Quantification of the neutrophil numbers in the blood of mock- (grey), or PVM- (purple) infected mice at day 5, 10, and 14 of infection.

**(h)** Quantification of the proportion of neutrophils out of CD45^+^ cells in the blood of mock- (grey), or PVM- (purple) infected mice at day 5, 10, and 14 of infection.

**(i)** Quantification of the neutrophil numbers in the blood of mock- (grey), or MCMV- (blue) infected mice at day 2, 5, and 8 of infection.

**(j)** Quantification of the proportion of neutrophils out of CD45^+^ cells in the blood of mock- (grey), or MCMV- (blue) infected mice at day 2, 5, and 8 of infection.

**Statistics:** (a-j) Two-way ANOVA, (G) 2-way ANOVA. * p < 0.05, ** p < 0.01, *** p < 0.001, **** p < 0.0001. Data representative of one of two experiments. Error bars represent SEM.

**Supplemental References**

1. Hao Y, Hao S, Andersen-Nissen E, et al. Integrated analysis of multimodal single-cell data. *Cell*. 2021;184(13):3573-3587.e29. doi:10.1016/j.cell.2021.04.048

2. Kelly Street, Davide Risso, Russell B. Fletcher, Diya Das, John Ngai, Nir Yosef EP and SD. Slingshot: cell lineage and pseudotime inference for single-cell transcriptomics. *BMC Genomics*. Published online 2010:1-16.

3. Van den Berge K, Roux de Bézieux H, Street K, et al. Trajectory-based differential expression analysis for single-cell sequencing data. *Nat Commun*. 2020;11(1):1-13. doi:10.1038/s41467-020-14766-3

4. Peterson H, Kolberg L, Raudvere U, Kuzmin I, Vilo J. gprofiler2 -- an R package for gene list functional enrichment analysis and namespace conversion toolset g: Profiler. *F1000Research*. 2020;9:1-27. doi:10.12688/f1000research.24956.2

5. Ferreira ACF, Szeto ACH, Heycock MWD, et al. RORα is a critical checkpoint for T cell and ILC2 commitment in the embryonic thymus. *Nat Immunol*. 2021;22(2):166-178. doi:10.1038/s41590-020-00833-w

6. Math E, Davis S. *Methods in Molecular Biology*. (Walker JM, ed.). Springer Nature; 2016.

7. Korsunsky I, Millard N, Fan J, et al. Fast, sensitive and accurate integration of single-cell data with Harmony. *Nat Methods*. 2019;16(12):1289-1296. doi:10.1038/s41592-019-0619-0

8. Browaeys R, Saelens W, Saeys Y. NicheNet: modeling intercellular communication by linking ligands to target genes. *Nat Methods*. 2020;17(2):159-162. doi:10.1038/s41592-019-0667-5

9. McCormack MP, Forster A, Drynan L, Pannell R, Rabbitts TH. The LMO2 T-Cell Oncogene Is Activated via Chromosomal Translocations or Retroviral Insertion during Gene Therapy but Has No Mandatory Role in Normal T-Cell Development . *Mol Cell Biol*. 2003;23(24):9003-9013. doi:10.1128/mcb.23.24.9003-9013.2003
