# Supplementary figures and images for: TL1A and IL-18 synergy promotes GM-CSF-dependent thymic granulopoiesis in mice
Source: Cell Mol Immunol. 2024 Jun 5;21(8):807–25. doi: 10.1038/s41423-024-01180-8 (PMC11291760; doi:10.1038/s41423-024-01180-8)

# Supplemental Figure 1

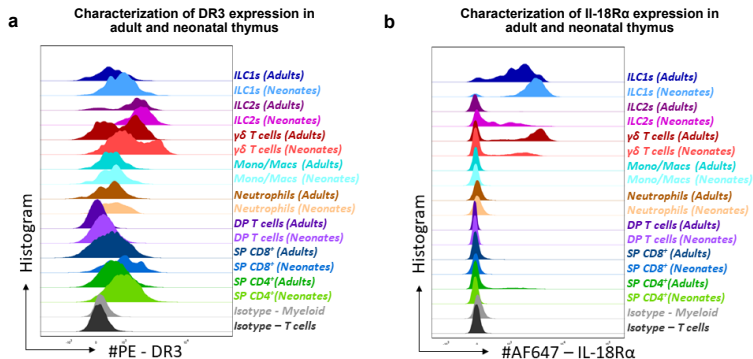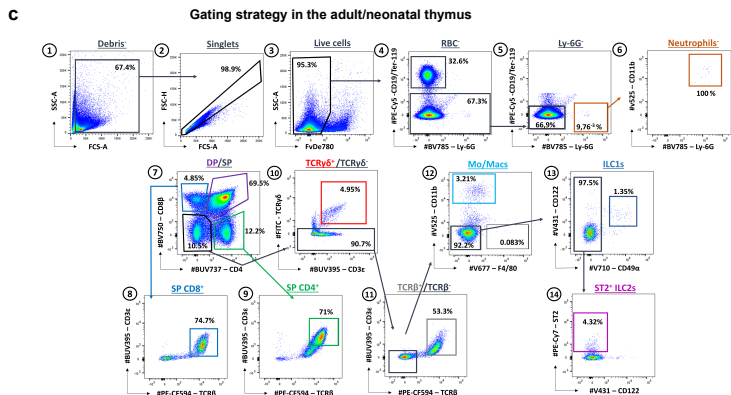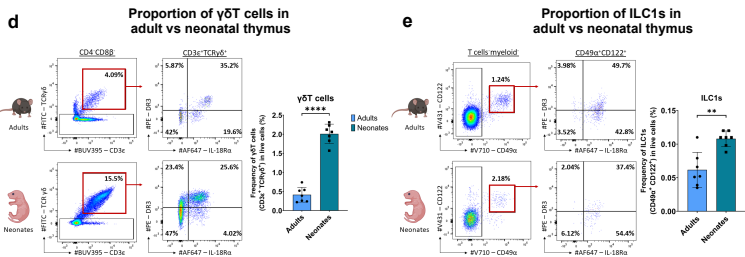

Supplement: Supplementary file 8 — Supplementary Figure 1 [file 41423_2024_1180_MOESM8_ESM.pdf]

# Supplementary Figure 2

a

TEM shows TL1A+IL-18 induced thymic atrophy

NTOC Lobes (Day 6)

PBS

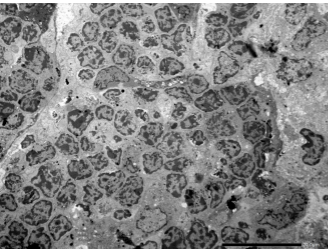

TL1A+IL-18

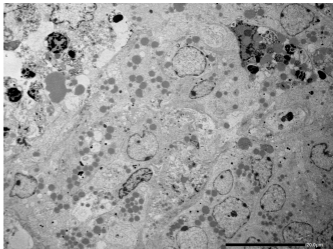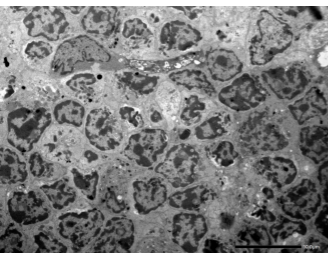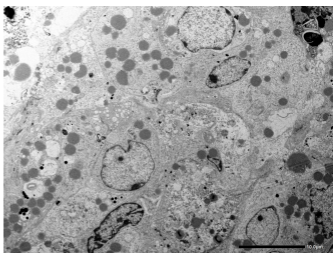

Supplement: Supplementary file 9 — Supplementary Figure 2 [file 41423_2024_1180_MOESM9_ESM.pdf]

# Supplementary Figure 4

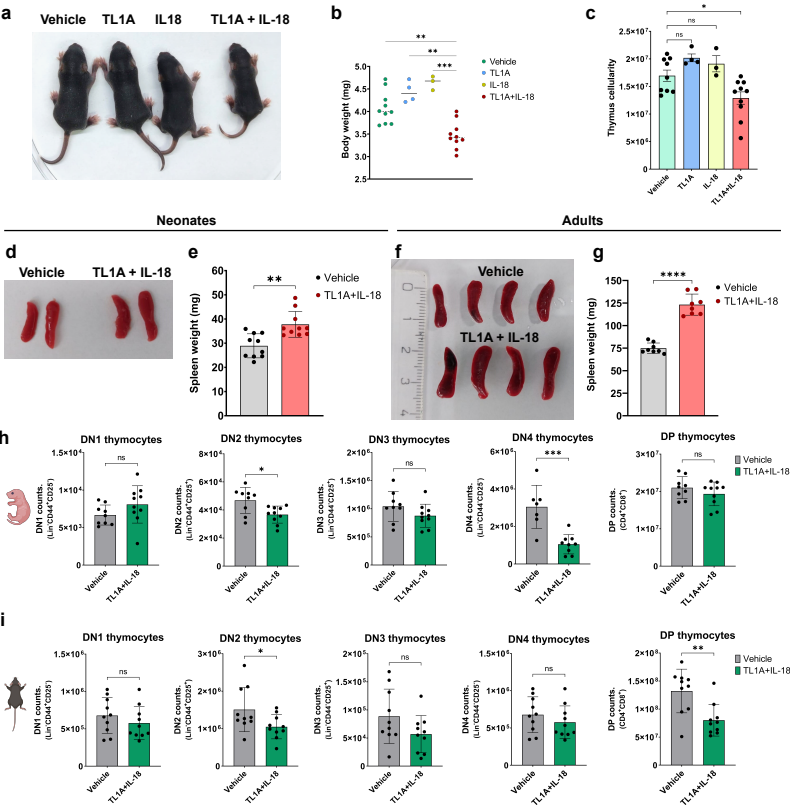

Supplement: Supplementary file 11 — Supplementary Figure 4 [file 41423_2024_1180_MOESM11_ESM.pdf]

# Supplementary Figure 5

PBS (Vehicle)

TL1A+IL-18

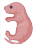  
**Neonate  
P7**

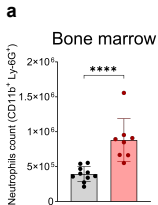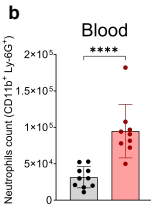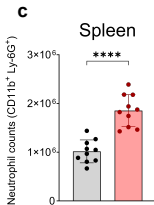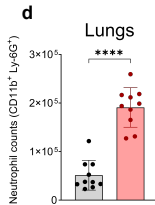

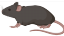  
**Adult  
8 w.o.**

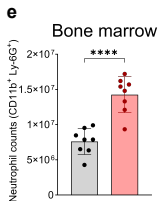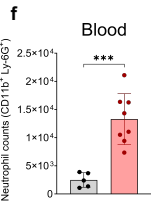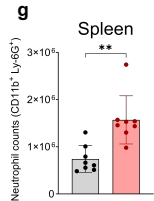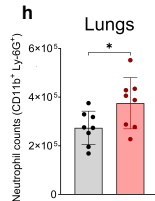

Supplement: Supplementary file 12 — Supplementary Figure 5 [file 41423_2024_1180_MOESM12_ESM.pdf]

# Supplementary Figure 7

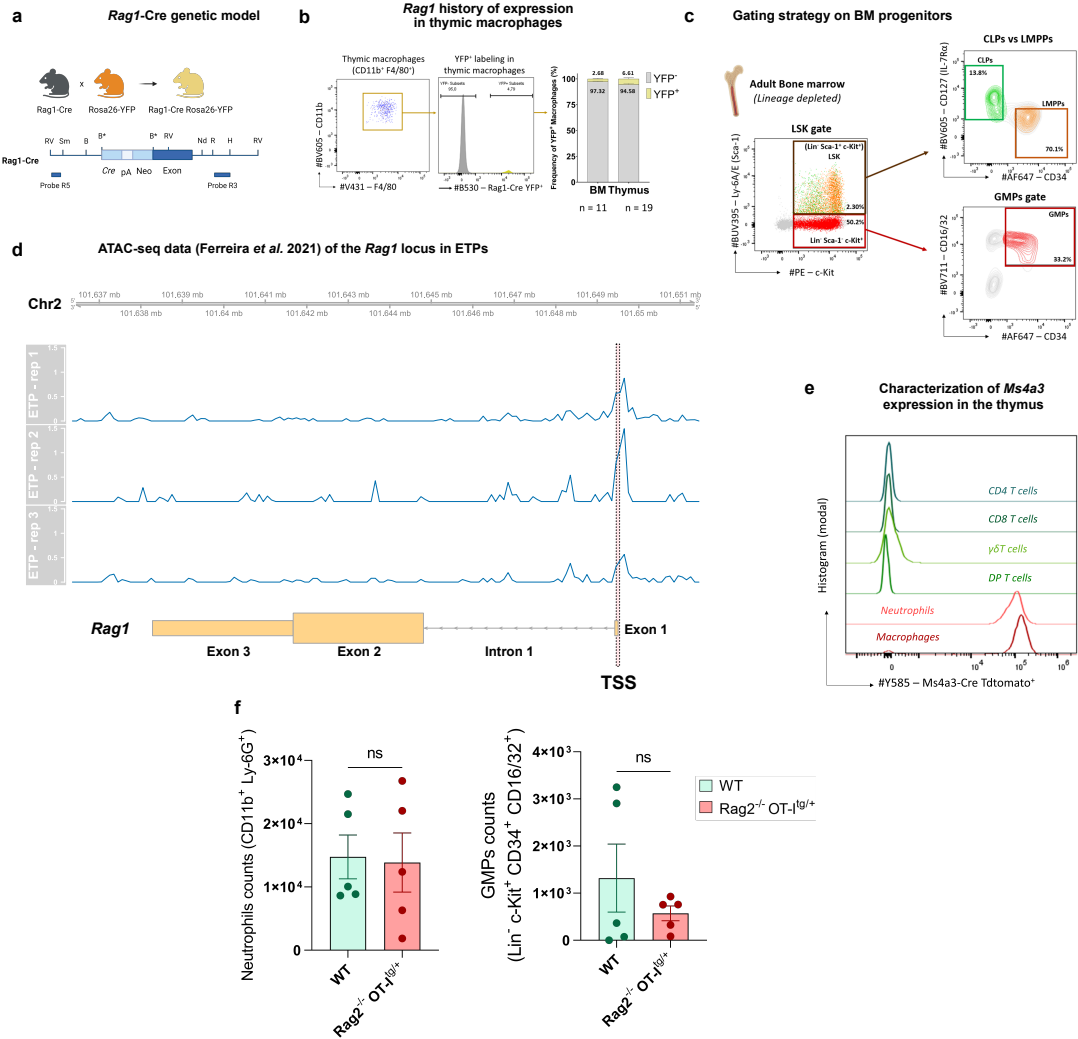

Supplement: Supplementary file 14 — Supplementary Figure 7 [file 41423_2024_1180_MOESM14_ESM.pdf]

# Supplementary Figure 8

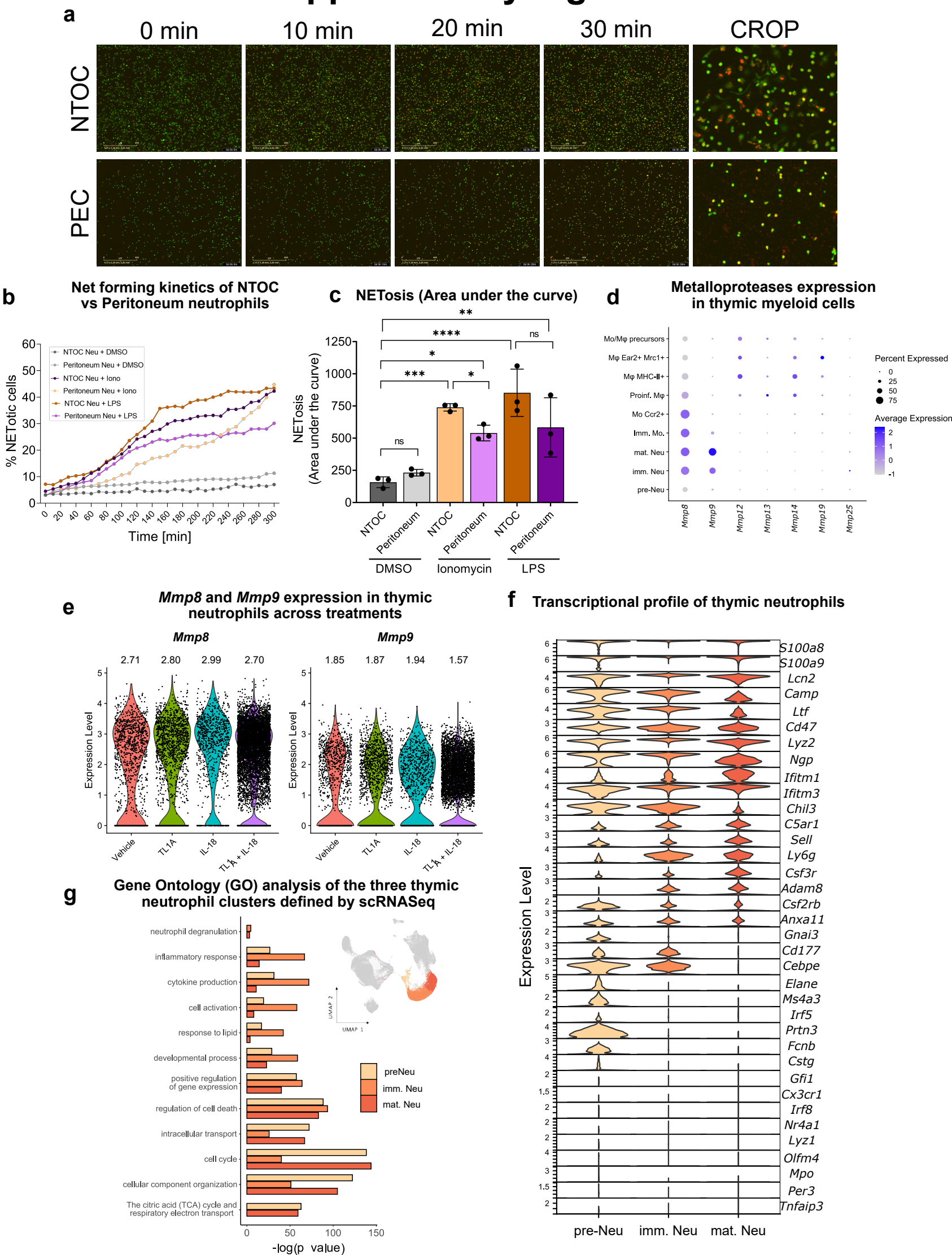

Supplement: Supplementary file 15 — Supplementary Figure 8 [file 41423_2024_1180_MOESM15_ESM.pdf]

# Supplementary Figure 10

a

Manually curated DE genes of 20 defined clusters

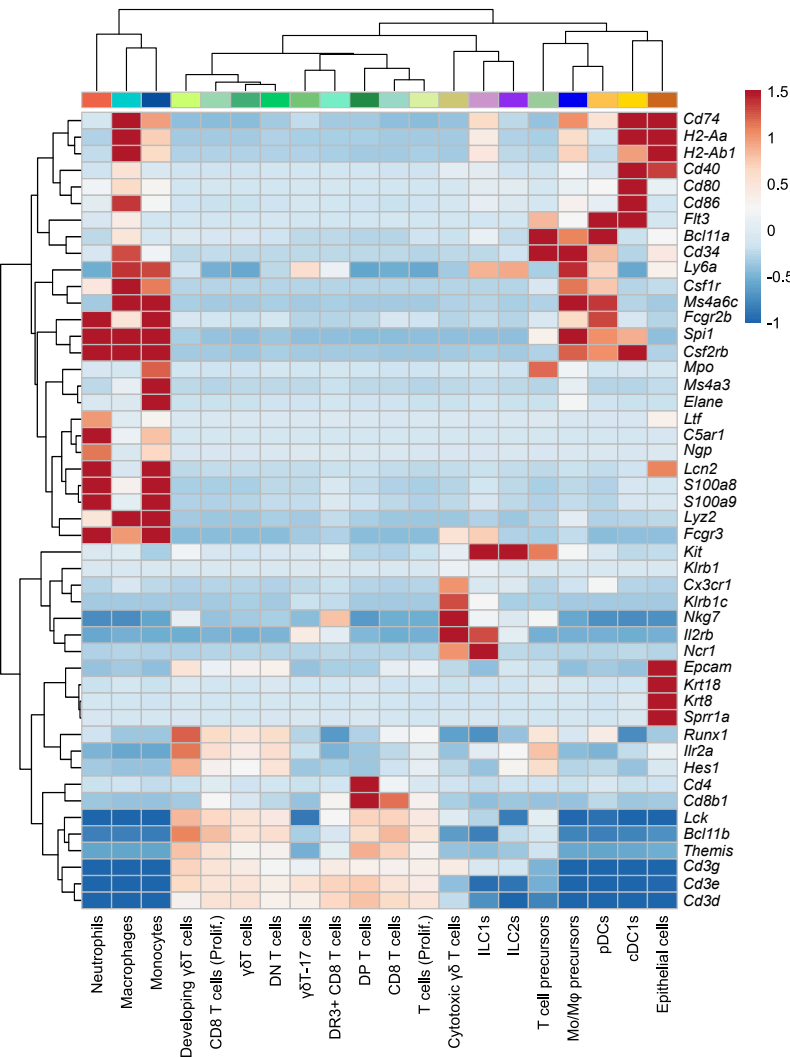

Supplement: Supplementary file 17 — Supplementary Figure 10 [file 41423_2024_1180_MOESM17_ESM.pdf]

# Supplementary Figure 13

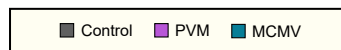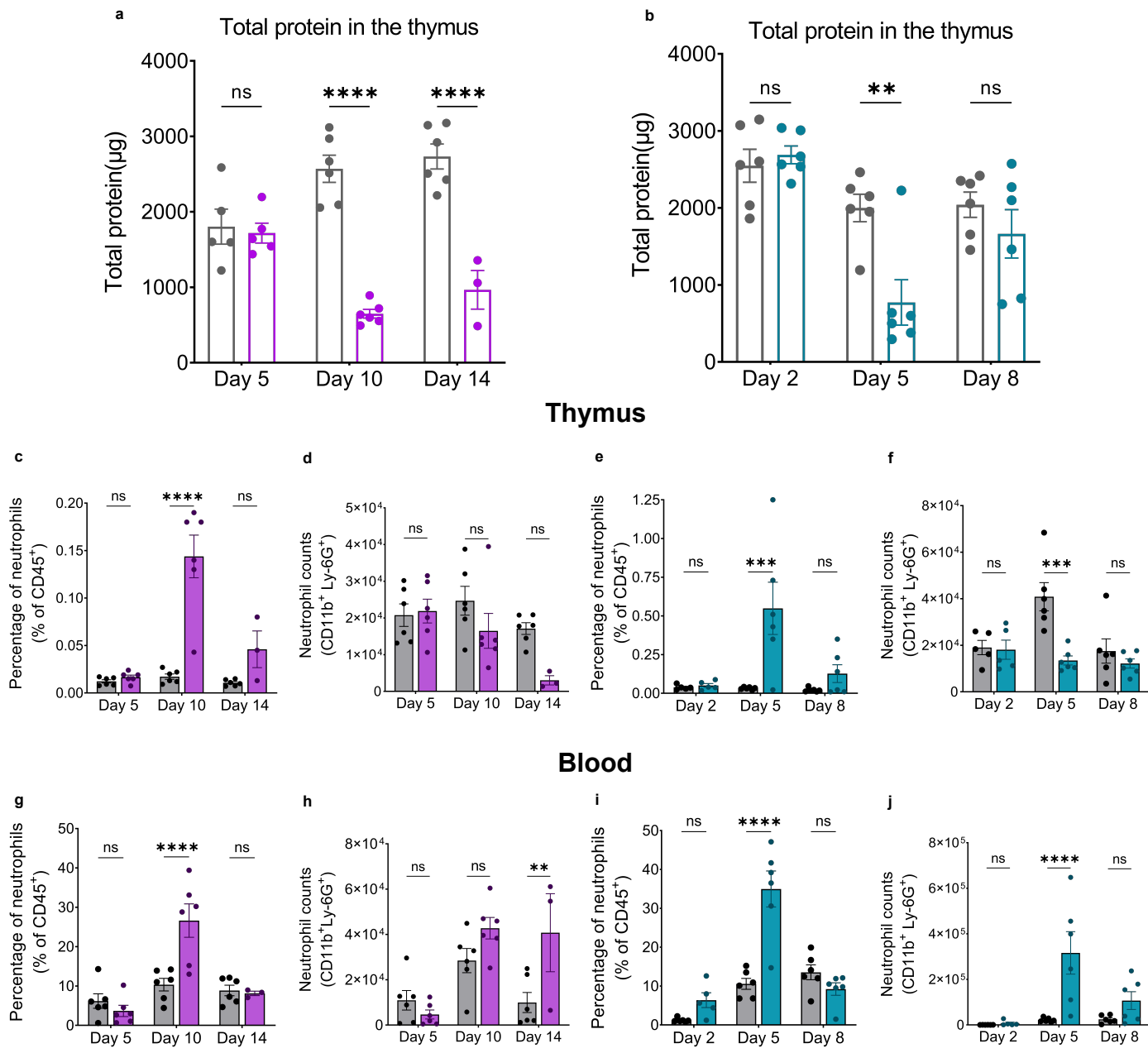

Supplement: Supplementary file 20 — Supplementary Figure 13 [file 41423_2024_1180_MOESM20_ESM.pdf]
