# Supplementary material for: TL1A and IL-18 synergy promotes GM-CSF-dependent thymic granulopoiesis in mice
Source: Cell Mol Immunol. 2024 Jun 5;21(8):807–25. doi: 10.1038/s41423-024-01180-8 (PMC11291760; doi:10.1038/s41423-024-01180-8)

Supplemental Figure 3

a CITE-Seq feature plots RNA vs ADT (proteins)

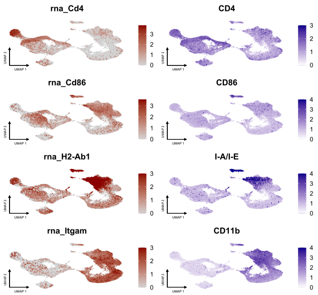

b Heatmap of CITE-Seq ADT (proteins)

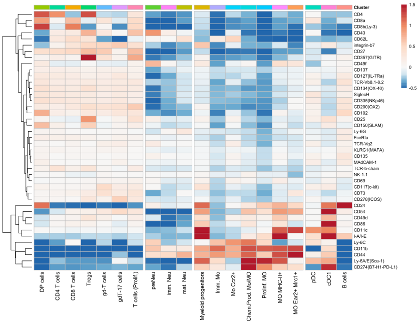

Supplement: Supplementary file 10 — Supplementary Figure 3 [file 41423_2024_1180_MOESM10_ESM.pdf]
