# Supplementary material for: TL1A and IL-18 synergy promotes GM-CSF-dependent thymic granulopoiesis in mice
Source: Cell Mol Immunol. 2024 Jun 5;21(8):807–25. doi: 10.1038/s41423-024-01180-8 (PMC11291760; doi:10.1038/s41423-024-01180-8)

Supplementary Figure 6

a Differentially expressed genes between developmental stages of thymic neutrophils

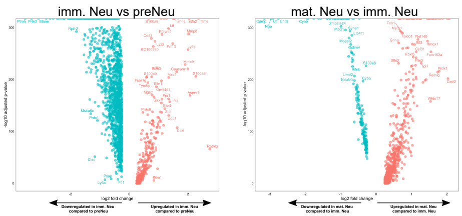

b Differentially expressed genes in thymic neutrophils between TL1A + IL-18 vs Vehicle

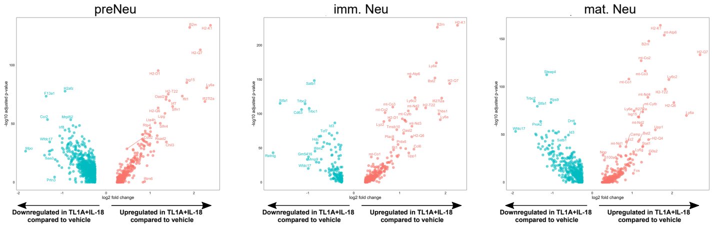

c Pseudotime analysis of genes involved in neutrophil development

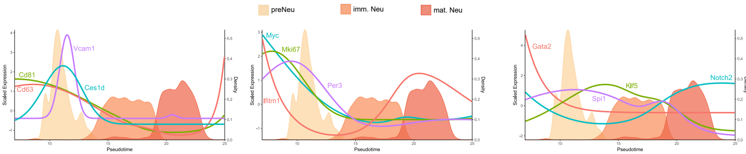

d TL1A + IL-18 treatment induces changes in transcription factors involved in T- and myeloid cell development

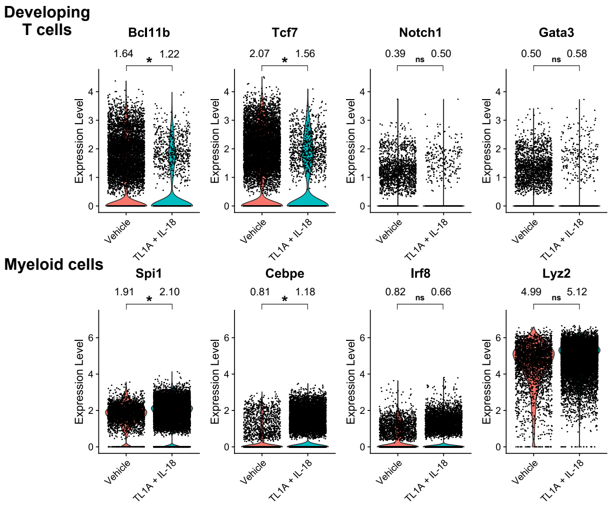

Supplement: Supplementary file 13 — Supplementary Figure 6 [file 41423_2024_1180_MOESM13_ESM.pdf]
