# Supplementary material for: TL1A and IL-18 synergy promotes GM-CSF-dependent thymic granulopoiesis in mice
Source: Cell Mol Immunol. 2024 Jun 5;21(8):807–25. doi: 10.1038/s41423-024-01180-8 (PMC11291760; doi:10.1038/s41423-024-01180-8)

Supplementary Figure 9

a Proinflammatory cytokines produced in response to synergistic effect of TL1A+IL-18

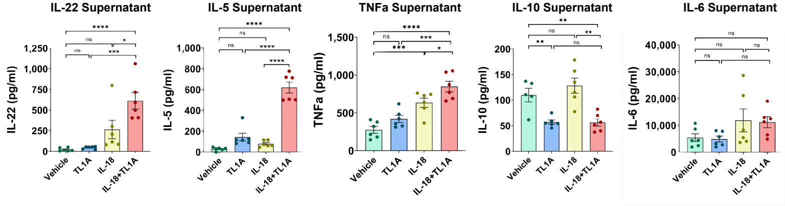

b Split UMAP clustering per condition

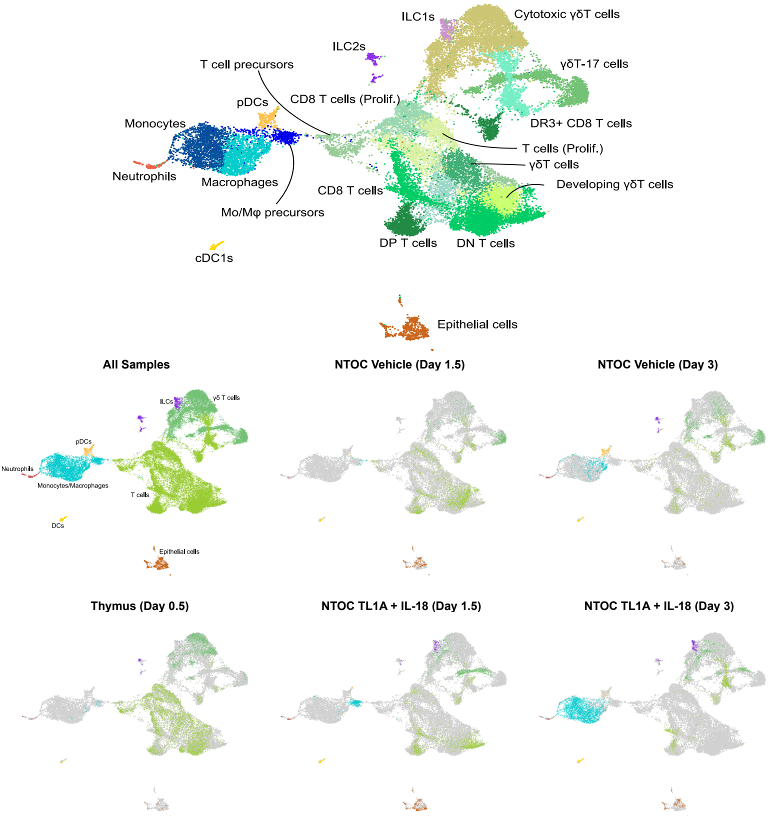

Supplement: Supplementary file 16 — Supplementary Figure 9 [file 41423_2024_1180_MOESM16_ESM.pdf]
