# Supplementary material for: TL1A and IL-18 synergy promotes GM-CSF-dependent thymic granulopoiesis in mice
Source: Cell Mol Immunol. 2024 Jun 5;21(8):807–25. doi: 10.1038/s41423-024-01180-8 (PMC11291760; doi:10.1038/s41423-024-01180-8)

Supplementary Figure 11

a

NicheNET analysis of ligand-pairs receptor interactions per condition/day

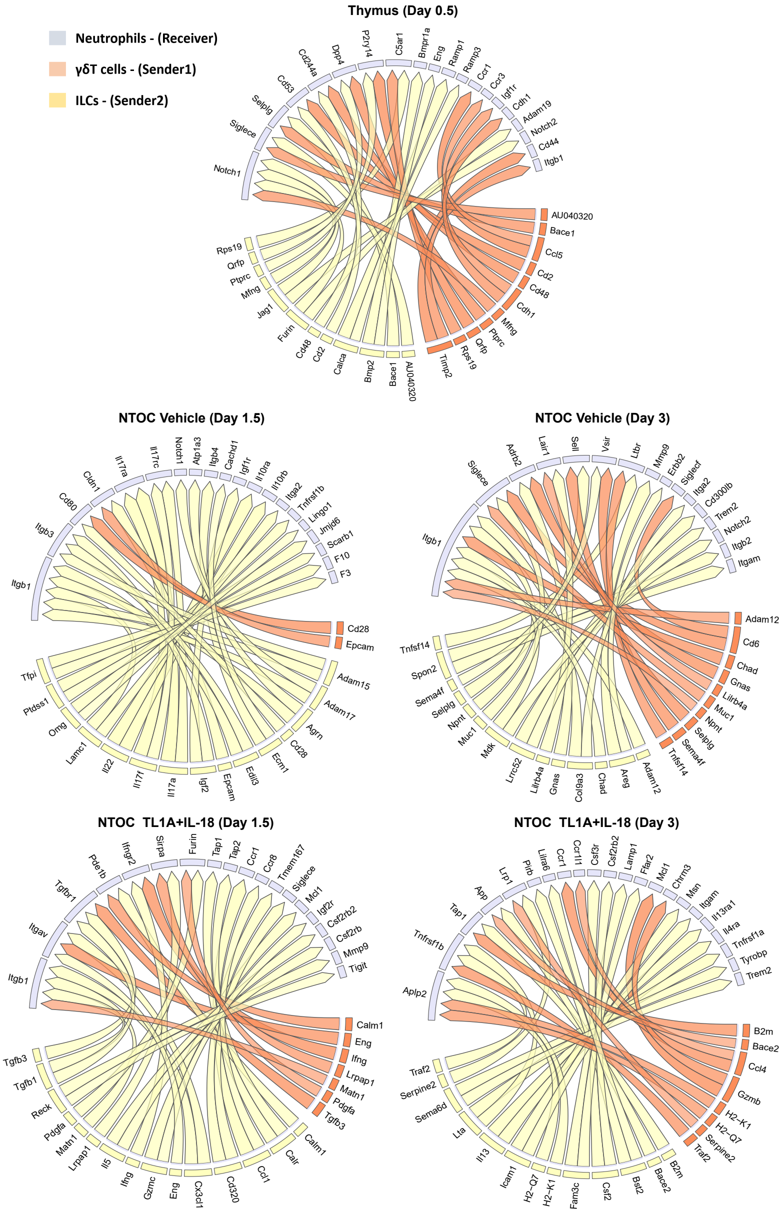

Supplement: Supplementary file 18 — Supplementary Figure 11 [file 41423_2024_1180_MOESM18_ESM.pdf]
